# Self-Reported Medication Use Across Racial and Rural or Urban Subgroups of People Who Are Pregnant in the United States: Decentralized App-Based Cohort Study

**DOI:** 10.2196/50867

**Published:** 2023-11-28

**Authors:** Toluwalase Ajayi, Jeff Pawelek, Hansa Bhargava, Arij Faksh, Jennifer Radin

**Affiliations:** 1 Scripps Research Translational Institute Scripps Research La Jolla, CA United States; 2 Faculty of Pediatrics and Medicine University of California, San Diego La Jolla, CA United States; 3 WebMD Atlanta Georgia; 4 Scripps Health La Jolla, CA United States

**Keywords:** prenatal care, maternal health, digital study, underrepresented in biomedical research, pregnant, pregnancy, medications, vaccinations, vitamins

## Abstract

**Background:**

Maternal health outcomes have been underresearched due to people who are pregnant being underrepresented or excluded from studies based on their status as a vulnerable study population. Based on the available evidence, Black people who are pregnant have dramatically higher maternal morbidity and mortality rates compared to other racial and ethnic groups. However, insights into prenatal care—including the use of medications, immunizations, and prenatal vitamins—are not well understood for pregnant populations, particularly those that are underrepresented in biomedical research. Medication use has been particularly understudied in people who are pregnant; even though it has been shown that up to 95% of people who are pregnant take at least 1 or more medications. Understanding gaps in use could help identify ways to reduce maternal disparities and optimize maternal health outcomes.

**Objective:**

We aimed to characterize and compare the use of prenatal vitamins, immunizations, and commonly used over-the-counter and prescription medications among people who are pregnant, those self-identifying as Black versus non-Black, and those living in rural versus urban regions in the United States.

**Methods:**

We conducted a prospective, decentralized study of 4130 pregnant study participants who answered survey questionnaires using a mobile research app that was only available on iOS (Apple Inc) devices. All people who were pregnant, living in the United States, and comfortable with reading and writing in English were eligible. The study was conducted in a decentralized fashion with the use of a research app to facilitate enrollment using an eConsent and self-reported data collection.

**Results:**

Within the study population, the use of prenatal vitamins, antiemetics, antidepressants, and pain medication varied significantly among different subpopulations underrepresented in biomedical research. Black participants reported significantly lower frequencies of prenatal vitamin use compared to non-Black participants (*P*<.001). The frequency of participants who were currently receiving treatment for anxiety and depression was also lower among Black and rural groups compared to their non-Black and urban counterparts, respectively. There was significantly lower use of antidepressants (*P*=.002) and antiemetics (*P*=.02) among Black compared to non-Black participants. While prenatal vitamin use was lower among participants in rural areas, the difference between rural and urban groups did not reach statistical significance (*P*=.08). There were no significant differences in vaccine uptake for influenza or tetanus-diphtheria-pertussis (TDaP) across race, ethnicity, rural, or urban status.

**Conclusions:**

A prospective, decentralized app-based study demonstrated significantly lower use of prenatal vitamins, antiemetics, and antidepressants among Black pregnant participants. Additionally, significantly fewer Black and rural participants reported receiving treatment for anxiety and depression during pregnancy. Future research dedicated to identifying the root mechanisms of these differences can help improve maternal health outcomes, specifically for diverse communities.

## Introduction

The maternal morbidity and mortality rate in the United States continues to exceed the rate in similar high-income countries, with rates drastically higher for Black people who are pregnant [1-3]. There are also differences in outcomes between urban and rural populations, in part due to a significant decline in rural hospital-based obstetrical services over the past 2 decades [4]. Separate reports have shown that maternal mortality rates are higher in rural areas and lower in urban and large metropolitan areas [5,6]. The shortage of maternity care providers puts Black people who are pregnant and those living in rural areas at risk of delaying or omitting 2 essential activities during the perinatal period: (1) seeking care at an early stage in pregnancy and (2) building trust with providers [7-10]. However, additional evidence that links the adoption of prenatal care with maternal health outcomes is needed [11]. Unfortunately, maternal health has been underresearched due to people who are pregnant being underrepresented or excluded from studies based on their status as a vulnerable population [12]. Medication use, safety, dosing, and efficacy have been particularly understudied in people who are pregnant, even though it has been shown that up to 95% of people who are pregnant take medications [13]. More specifically, the use of medications, immunizations, and prenatal vitamins across underrepresented pregnant populations is not well understood. There is significant evidence that vitamin supplementation can prevent a multitude of problems during pregnancy, yet there is no nationally recognized standard on the minimum dose of each vitamin and many prenatal vitamins do not include all of the generally recommended vitamins [14]. Understanding the underlying reasons behind why there are gaps in use could help identify ways to reduce maternal disparities between demographic groups and optimize pregnancy outcomes [15]. As the US health care system struggles with developing solutions to improve patient outcomes, several federally supported pregnancy registries have recently been launched—and at least 1 of these programs is aimed at answering questions about medication use during pregnancy [16]. The aim of this report is to characterize and compare the use of prenatal vitamins and commonly used over-the-counter and prescription medications among people who are pregnant, those self-identifying as Black versus non-Black, and those living in rural versus urban regions in the United States.

## Methods

### Overview

The results of this report come from The Healthy Pregnancy Study, an institutional review board (IRB)–approved prospective, observational, and decentralized study. The study was made available via a free iOS-based ResearchKit app, which was embedded in a WebMD pregnancy app, between March 16, 2017 and December 31, 2020. Instead of using physical study sites, the research app was used to enroll and gather self-reported data on all study participants. All participants were recruited from the WebMD pregnancy app [17]. All people who were pregnant, living in the United States, had an iPhone, and were comfortable with reading and writing in English were eligible. The study was conducted in a decentralized fashion with the use of a research app to facilitate enrollment using an eConsent and self-reported data collection. Upon enrollment, participants were asked to complete an initial intake questionnaire to collect demographic information (including race or ethnicity and zip code) and a health history survey to capture current health conditions (including anxiety or depression), vaccinations (including influenza or tetanus-diphtheria-pertussis [TDaP]), prescription medications (free-text response), over-the-counter medications (free-text response), and the use of prenatal vitamins (yes or no binary response; Multimedia Appendices 1 and 2). Of note, there was a concern if the survey questions were too detailed or technical, participants may be unable to report accurate and complete information (or worse, may discontinue their study participation over confusion or burden of such a survey). Therefore, we specifically chose general vitamin and medication categories to ensure a large cohort would be able to fully understand the questions being asked and respond with reliable information. Participants could select as many responses as they wished for the question “What is your race/ethnicity?” Those who selected Black or African American were categorized as Black, and everyone else who answered the race or ethnicity question was categorized as non-Black. Zip codes that did not have a metropolitan statistical area code were classified as rural. Participants could choose to skip any study survey question and could participate more than once for subsequent pregnancies. If a participant completed multiple surveys during their study participation, the first completed survey was included in the analysis of this report.

### Analysis

We used a chi-square test to compare the frequency of reported characteristics by race or ethnicity and rural or urban zip code to determine if there were any significant differences across different groups.

### Ethical Considerations

This study was approved by Scripps Health IRB (17-6924). All participants signed an IRB-approved electronic informed consent form. All results from this study have been completely anonymized. Study participants received no compensation for their participation in this study.

## Results

During the study period, 4130 individual participants filled out surveys on 4151 pregnancies. After excluding surveys from subsequent pregnancies for the same participant, there was a total of 4091 intake and 3645 health history surveys. Of the 4041 participants, 537 (13.3%) reported as being Black and 3504 (86.7%) reported as being non-Black. Of the 3855 participants who reported their zip code, 628 (16.3%) were from the rural areas and 3227 (83.7%) were from the urban areas. On average, the health history survey was completed during week 16 (SD 11) of pregnancy.

There were no significant differences in frequencies of vaccine uptake for influenza or TDaP across race, ethnicity, rural, or urban status (all *P*>.05). Black participants reported significantly lower frequencies of prenatal vitamin use compared to non-Black participants (*P*<.001). The frequency of participants who were currently receiving treatment for anxiety and depression also differed by both racial and rural or urban subgroups. There was significantly lower use of antidepressants (*P*=.002) and antiemetics (*P*=.02) among Black participants than non-Black participants (Table 1).

**Table 1:** Frequency of treatment for existing conditions and medication and vaccine use by race or ethnicity and zip code from the Short Intake Survey and Health History Questionnaire^a^ for singleton births from 4130 participants^b^.

Variable				Race or ethnicity									Zip code						
				Missing data, n		Black, n/N (%)		Non-Black, n/N (%)		*P* value		Missing data, n			Rural, n/N (%)		Urban, n/N (%)		*P* value
Participants				89		537/4041 (13.3)		3504/4041 (86.7)				275			628/3855 (16.3)		3227/3855 (83.7)		
**Vaccines, yes**																			
	Flu			567		86/483 (17.8)		604/3081 (19.6)		.36		718			99/550 (18)		570/2864 (19.9)		.29
	TDaP^c^			567		57/483 (11.8)		348/3079 (11.3)		.73		718			66/550 (12)		326/2859 (11.4)		.69
Prenatal vitamins, yes				574		422/482 (87.6)		2874/3073 (93.5)		<.001		721			502/551 (91.1)		2663/2857 (93.2)		.08
**Current treatment**				584						.003		731							.007
	Anxiety			N/A^d^		13/481 (2.7)		176/3087 (5.7)				N/A			18/545 (3.3)		164/2827 (5.8)		
	Depression			N/A		17/485 (3.5)		98/3062 (3.2)				N/A			64/551 (11.6)		317/2855 (11.1)		
	Both			N/A		39/481 (8.1)		358/3059 (11.7)				N/A			28/549 (5.1)		83/2862 (2.9)		
**Medications^e^**																			
	Antidepressants			N/A		17/531 (3.2)		268/3480 (7.7)		.002		N/A			43/623 (6.9)		233/3236 (7.2)		.76
	Anxiolytics			N/A		1/500 (0.2)		13/3250 (0.4)		.50		N/A			4/666 (0.6)		9/3000 (0.3)		.16
	**Pain medications**																		
		Opioids	N/A		0/500 (0)		19/3800 (0.5)		.09		N/A			5/625 (0.8)		13/3250 (0.4)		.19	
		Nonsteroidal anti-inflammatory drugs	N/A		2/500 (0.4)		25/3571 (0.7)		.37		N/A			5/625 (0.8)		21/3000 (0.7)		.69	
		Acetaminophen	N/A		28/538 (5.2)		175/3500 (5)		.83		N/A			27/627 (4.3)		169/3250 (5.2)		.33	
	Antiemetics			N/A		13/541 (2.4)		160/3478 (4.6)		.02		N/A			29/630 (4.6)		137/3186 (4.3)		.67
	Antibiotics			N/A		3/500 (0.6)		16/3200 (0.5)		.75		N/A			3/600 (0.5)		15/3000 (0.5)		.97

^a^Both surveys taken when a participant joins the study.

^b^Participants could choose to skip entire surveys or questions within the surveys resulting in different numbers of missing data throughout the table.

^c^TDaP: tetanus-diphtheria-pertussis

^d^N/A: not applicable.

^e^There was no survey response for “no medications,” so an absence of an answer was assumed to equal no medication but may result in underestimation.

## Discussion

This is one of the first prospective, decentralized studies to gather self-reported data on medication use by race or ethnicity and urban or rural zip code, providing insight into how medication use correlates with access to health care. There is growing evidence that social determinants of health impact pregnancy outcomes and overall maternal mortality in the United States. Social determinants of health and constructs of structural racism and discrimination, such as socioeconomic disadvantages, poor health literacy, transportation barriers, lack of access to adequate health care, food deserts, and psychosocial stressors, have cascading and downstream effects on perinatal outcomes [18-20]. Low-income, marginalized, and rural families are less likely to have health insurance, and these families spend an average of 19% to 30% of their annual income on pregnancy and childbirth-related medical expenses, not including additional costs related to nutrition and over-the-counter medications [15,21,22].

Leveraging the decentralized study model, which allows participants to self-enter data in real time, we enrolled 13.3% (537/4041) of Black participants and 16.3% (628/3855) of rural participants (compared to 14% and 19% of the US population, respectively) [23,24]. Over-the-counter medications, antiemetics, and prenatal vitamins are important for maintaining a healthy pregnancy and managing pain and other side effects. However, health-related costs and education may be a barrier for some populations, thus reducing access and uptake. Our results demonstrated lower use of prenatal vitamins among Black and rural participants, as well as significantly lower use of antiemetics among Black participants. There were also significant differences in the frequency of treatment for anxiety and depression across the populations including the use of antidepressants. Whether there is a connection between the lower use of antidepressants and the presence of untreated mental health disorders is unknown. Further studies to understand the prevalence of people who are pregnant with treated and untreated mental health conditions would help identify factors that contribute to medication use during pregnancy.

There are a few notable limitations of this study. One of the main limitations is that the cohort may not represent the general population with regard to digital health literacy and socioeconomic status since ownership of an iPhone and the use of a health app were required for study eligibility. Although we gathered information on other racial and ethnic groups, the number of participants in other subgroups was smaller and therefore were not included in this study. Additionally, the data provide a snapshot of vaccinations, vitamins, and medications taken at the time of enrollment, which on average occurred during the first trimester, but may not represent the use throughout the course of pregnancy. There is a range of causes for why these differences in medication use exist that were not identified by our surveys. Furthermore, the self-reported survey data were not verified with medical records and participants may not have accurately recalled their medication use at the time of the survey. Further studies to understand the causes of disparities in medication use are needed, including a combination of provider- and patient-reported data, to optimize the medical management and maternal health outcomes across all populations.

## Appendix

Multimedia Appendix 1 Participant-facing screenshots from the study app showing the onboarding and informed consent process.

Multimedia Appendix 2 Participant-facing screenshots from the study app showing the survey questions.

## Abbreviations

IRB: institutional review board
TDaP: tetanus-diphtheria-pertussis

## Data Availability

The data sets generated and analyzed in this study are available from the corresponding author on reasonable request.
