# Supplementary material for: Self-Reported Medication Use Across Racial and Rural or Urban Subgroups of People Who Are Pregnant in the United States: Decentralized App-Based Cohort Study
Source: JMIR Form Res. 2023 Nov 28;7:e50867. doi: 10.2196/50867 (PMC10716764; doi:10.2196/50867)
Supplement: Multimedia Appendix 1 [file formative_v7i1e50867_app1.pptx]

## Slide 1
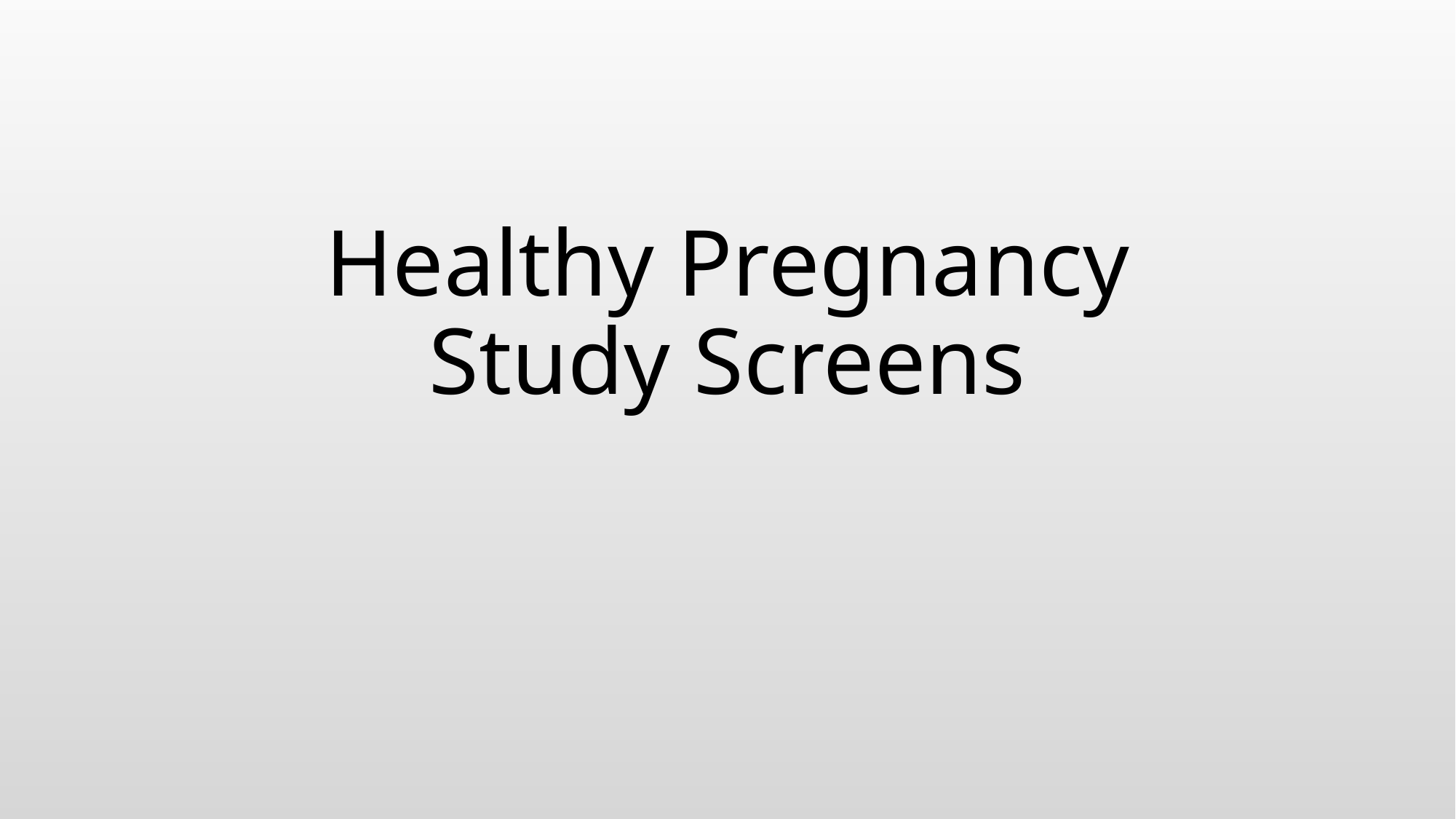

# Healthy Pregnancy Study Screens

## Slide 2
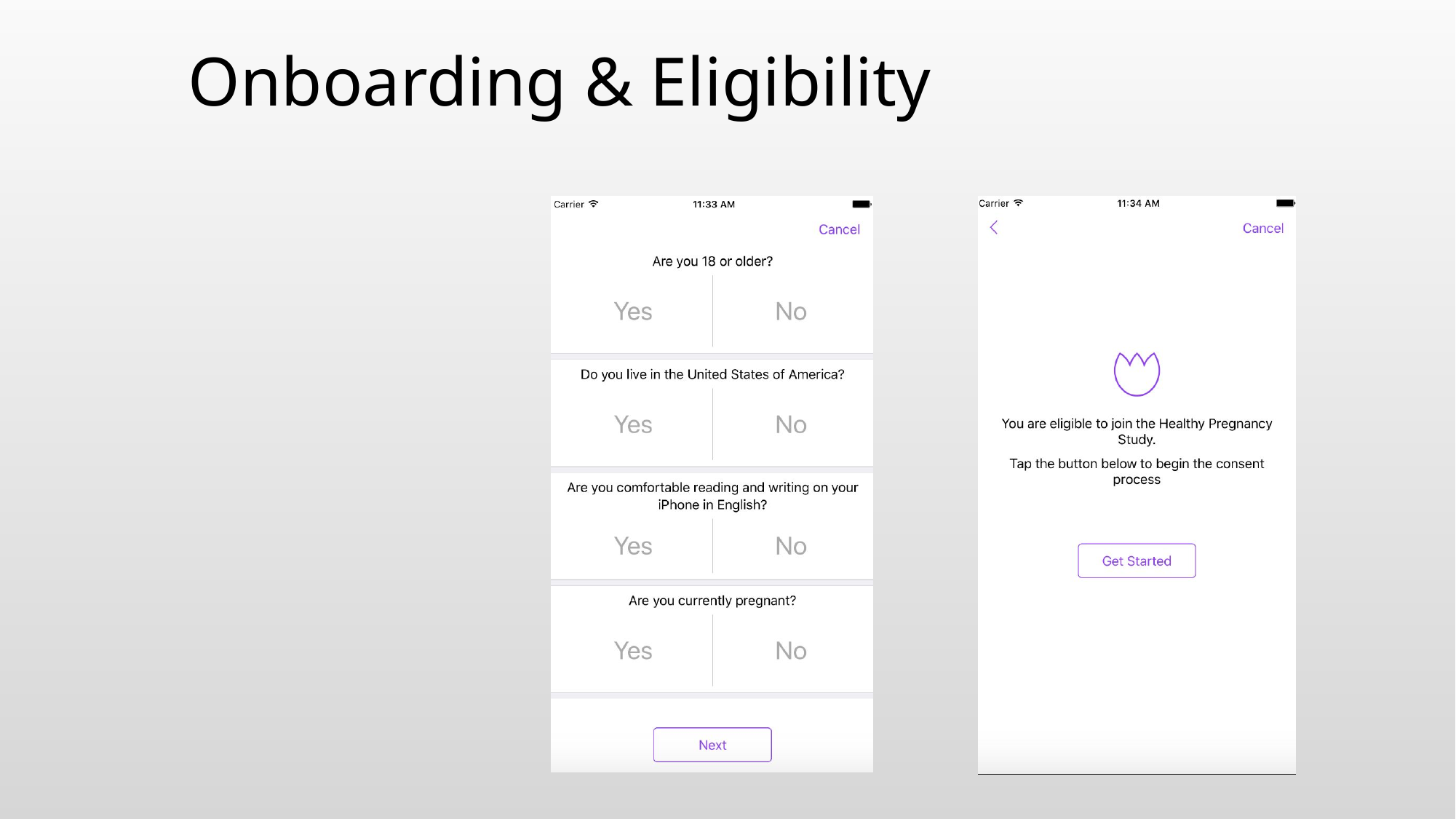

# Onboarding & Eligibility

## Slide 3
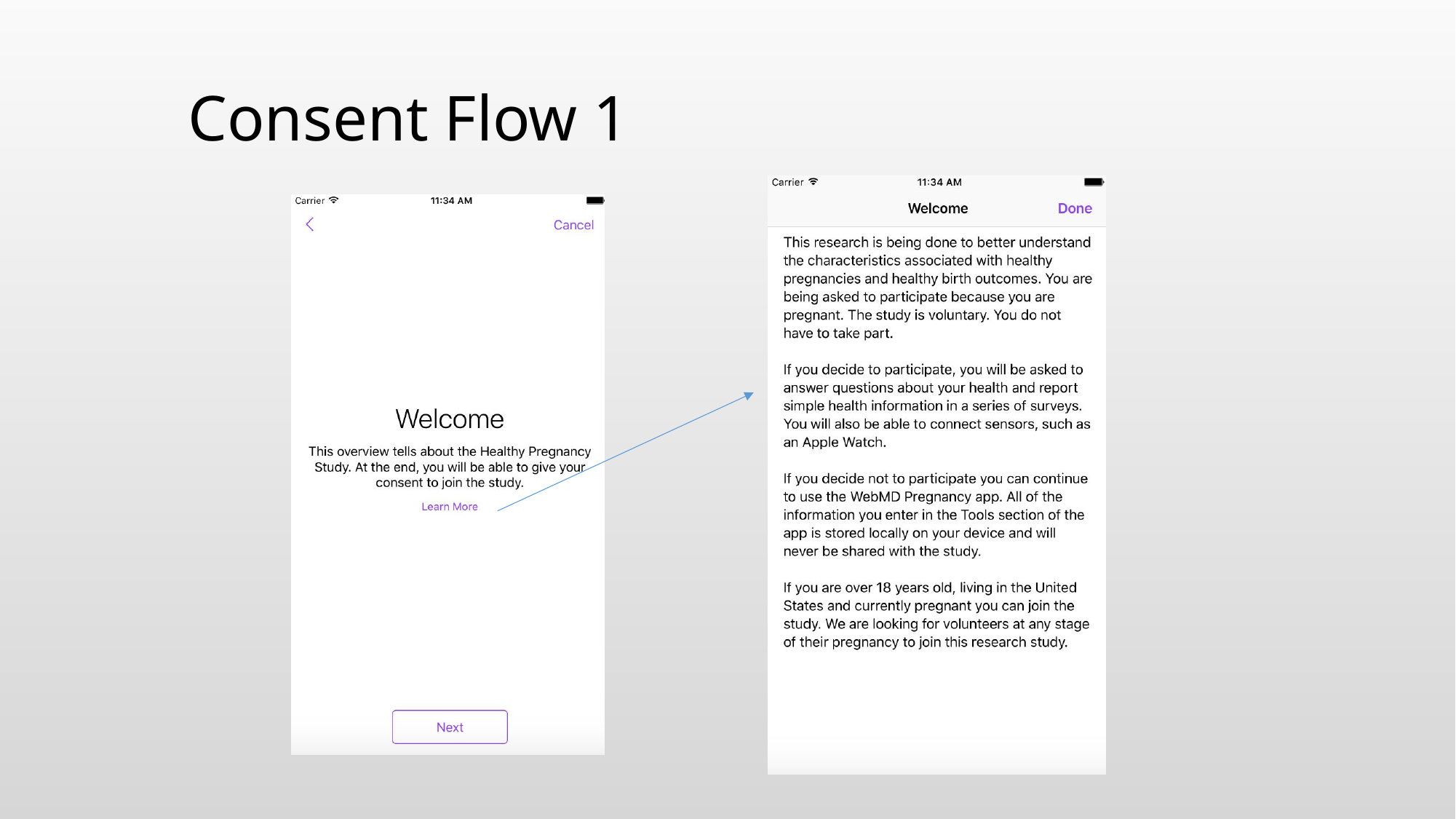

# Consent Flow 1

## Slide 4
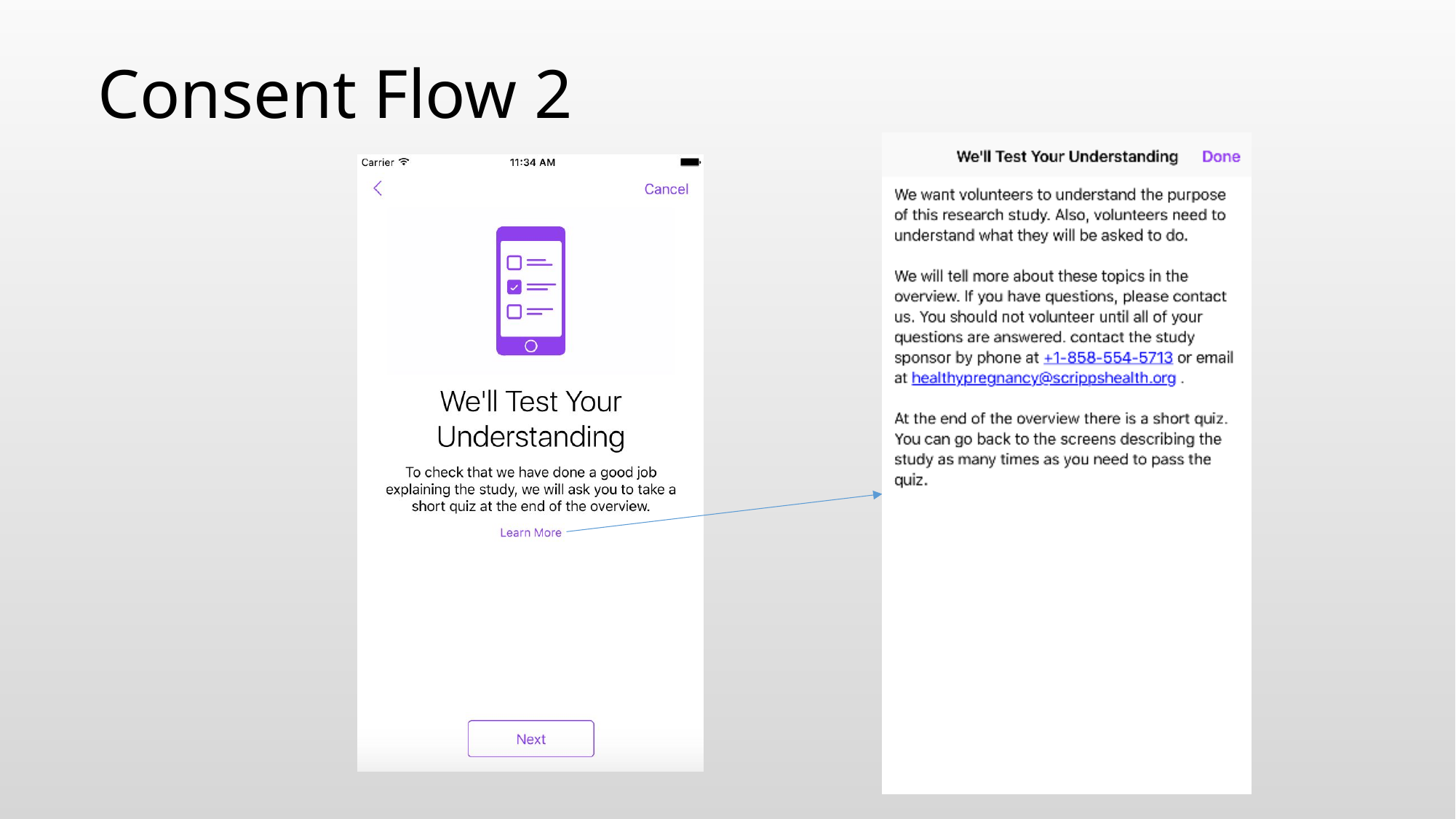

# Consent Flow 2

## Slide 5
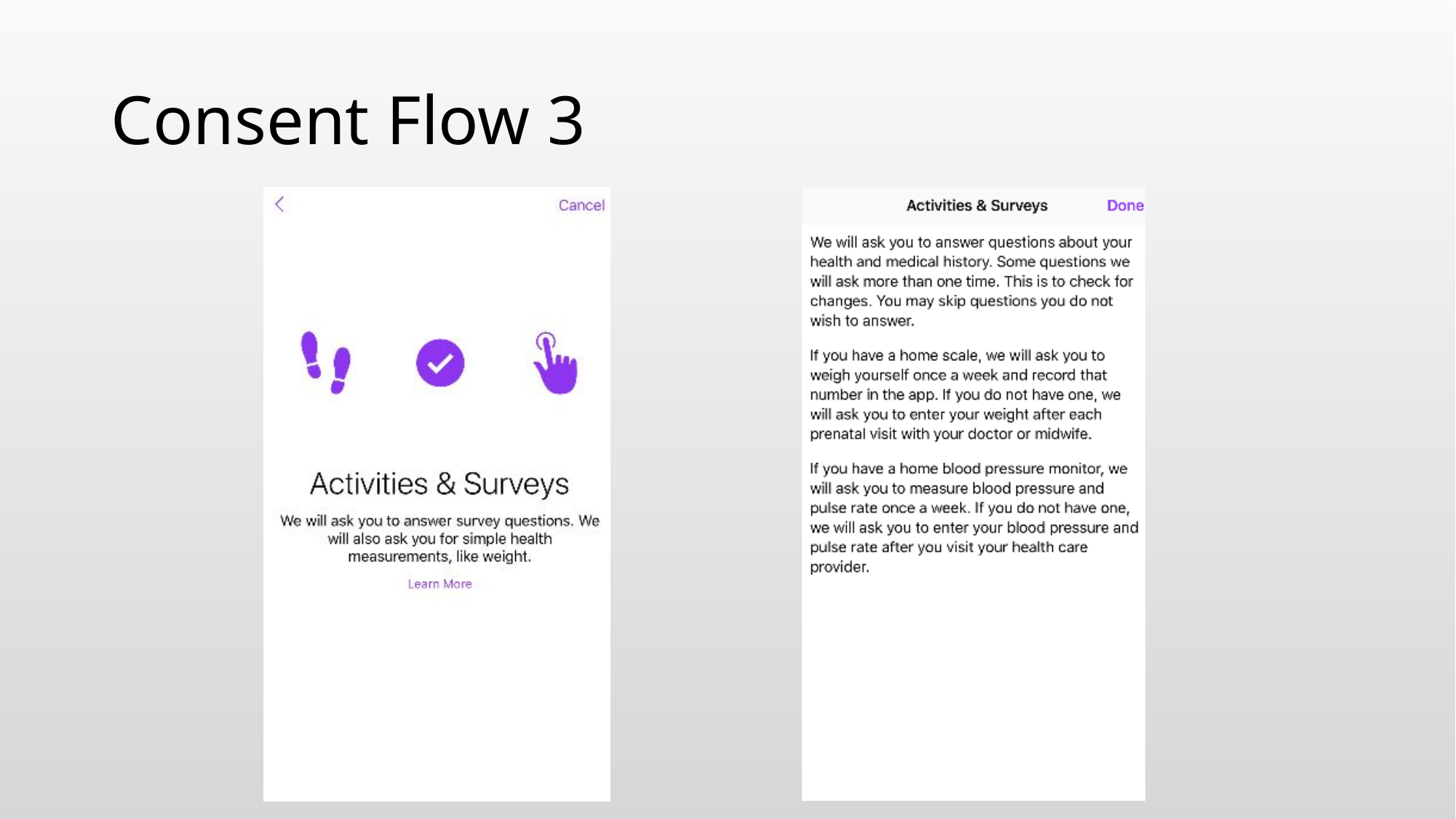

# Consent Flow 3

## Slide 6
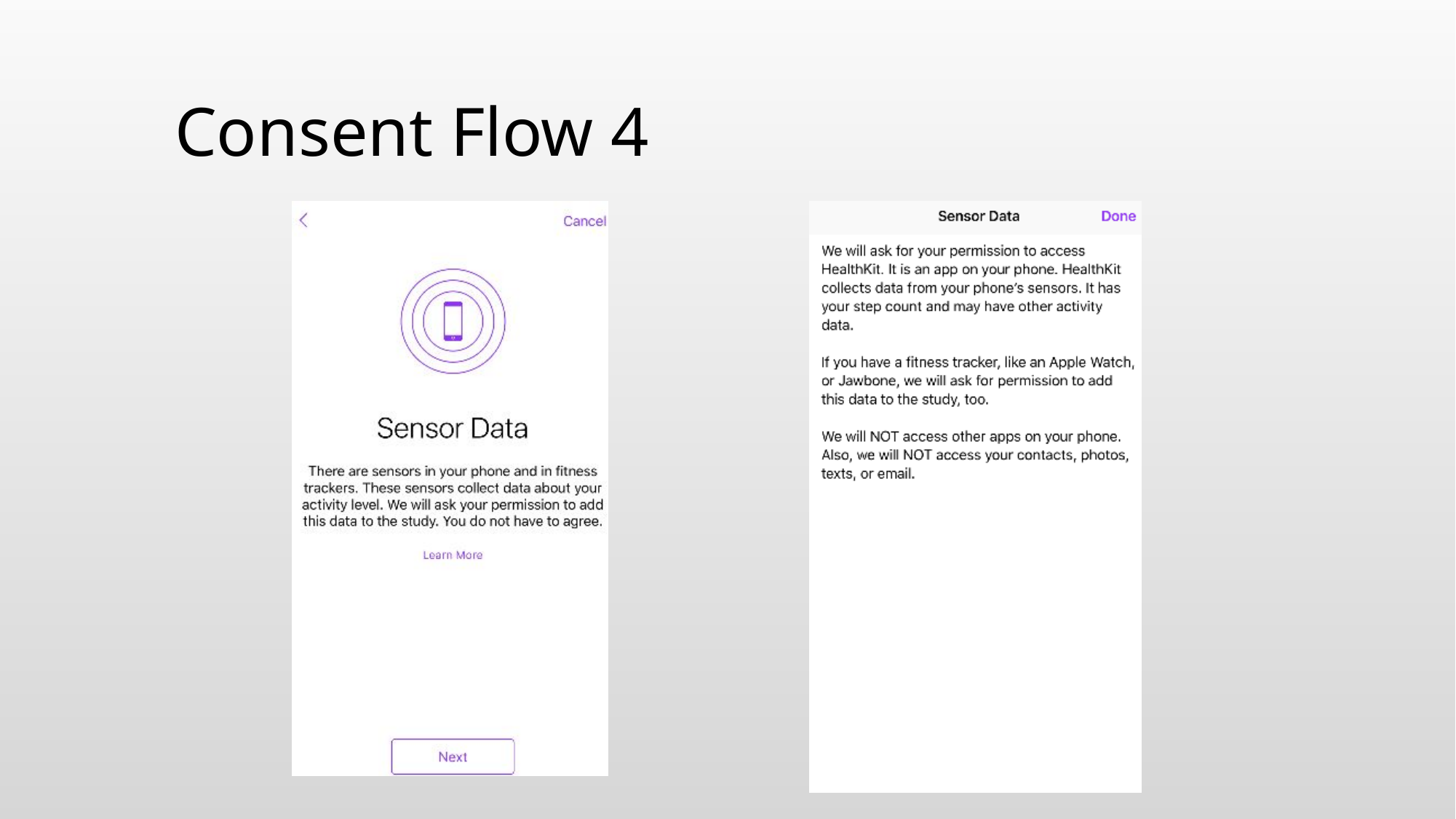

# Consent Flow 4

## Slide 7
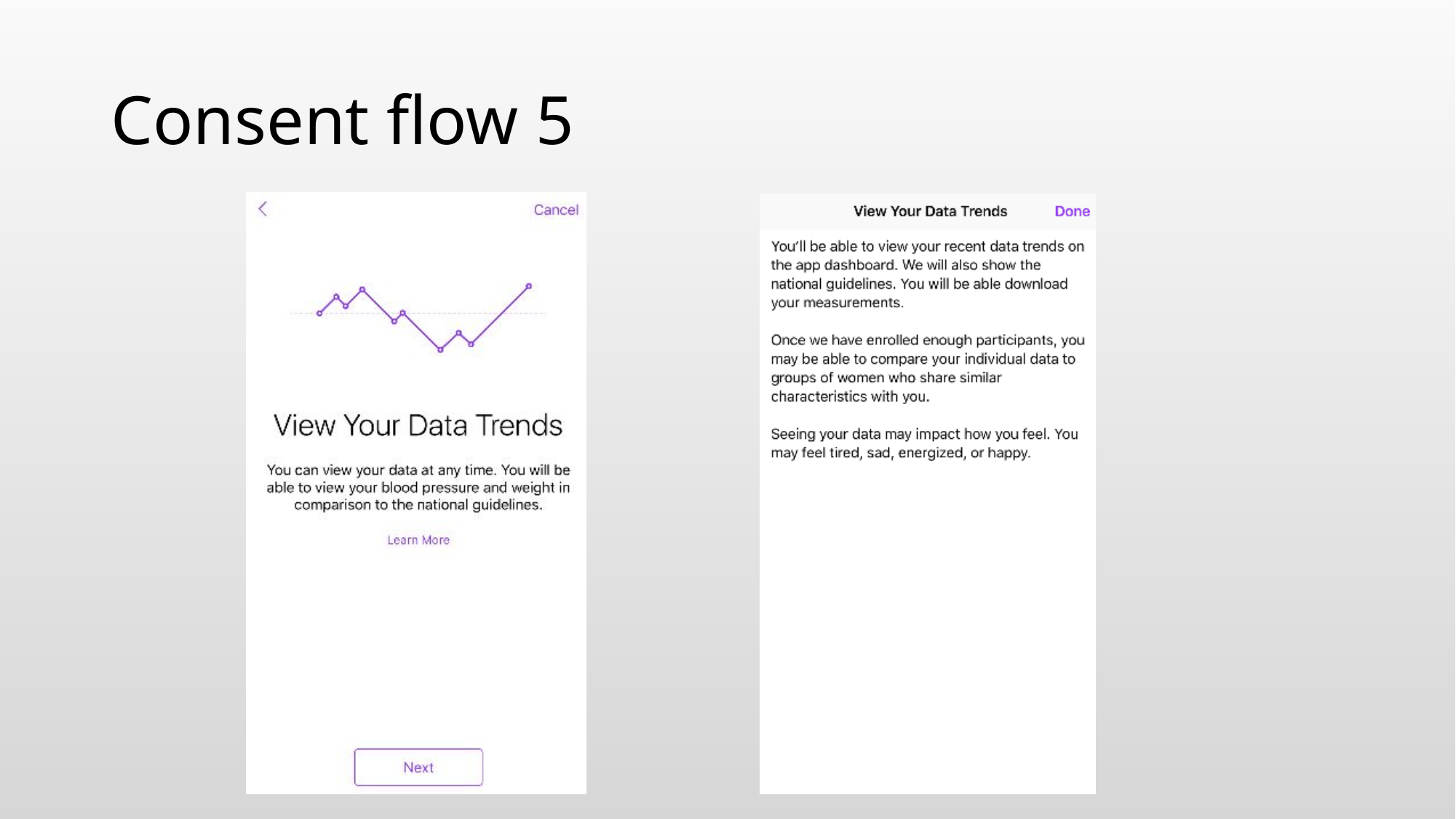

# Consent flow 5

## Slide 8
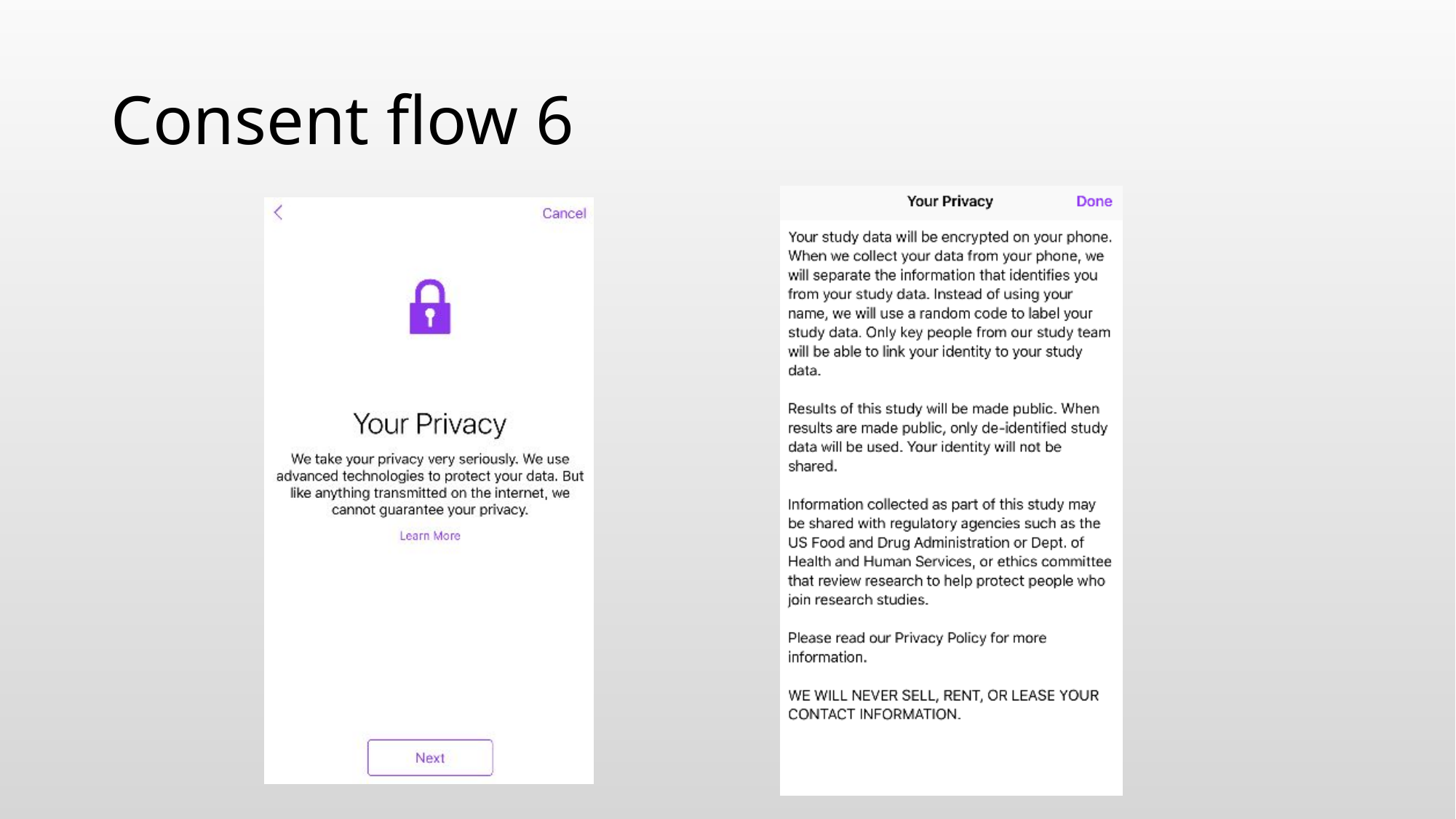

# Consent flow 6

## Slide 9
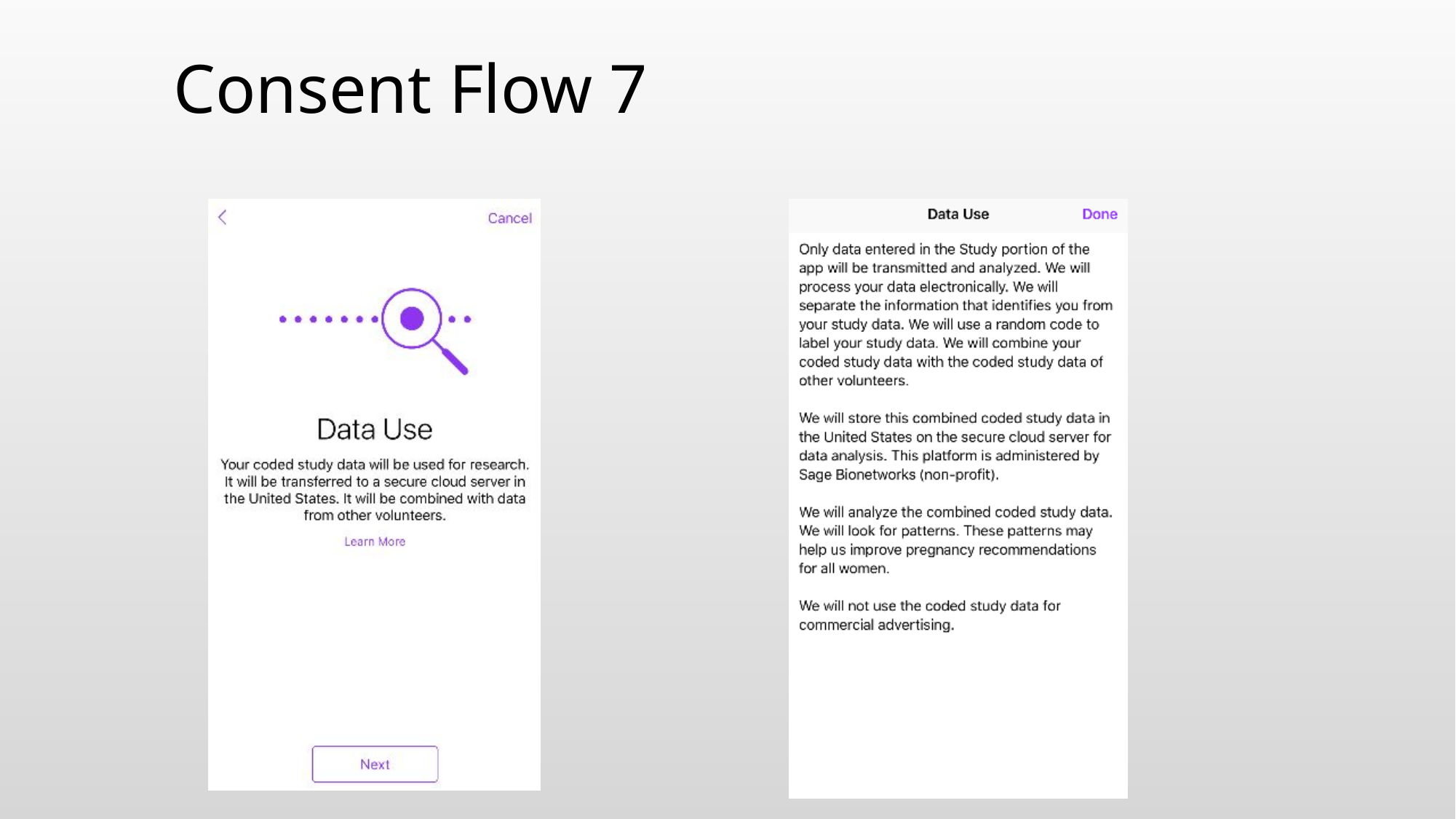

# Consent Flow 7

## Slide 10
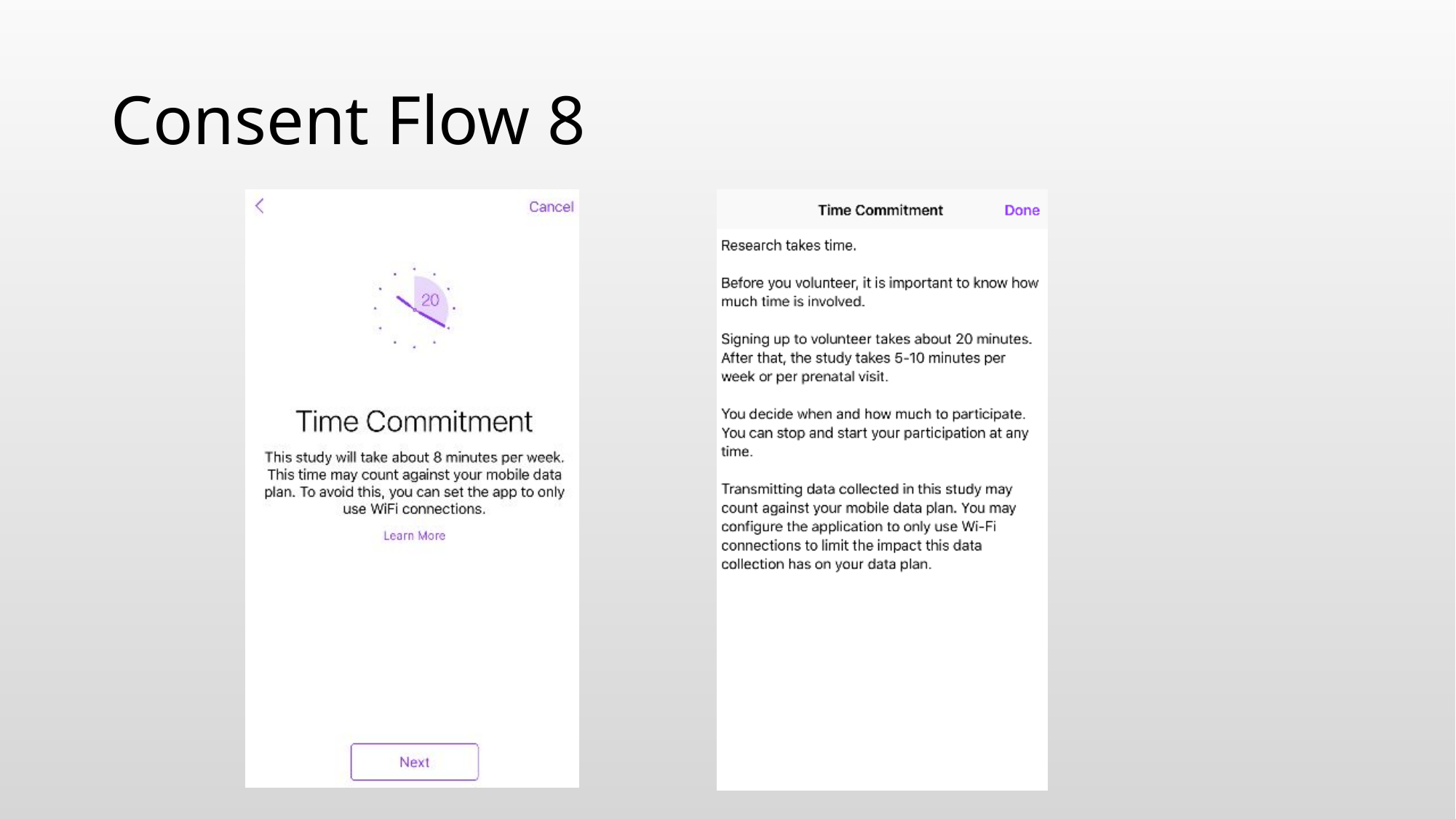

# Consent Flow 8

## Slide 11
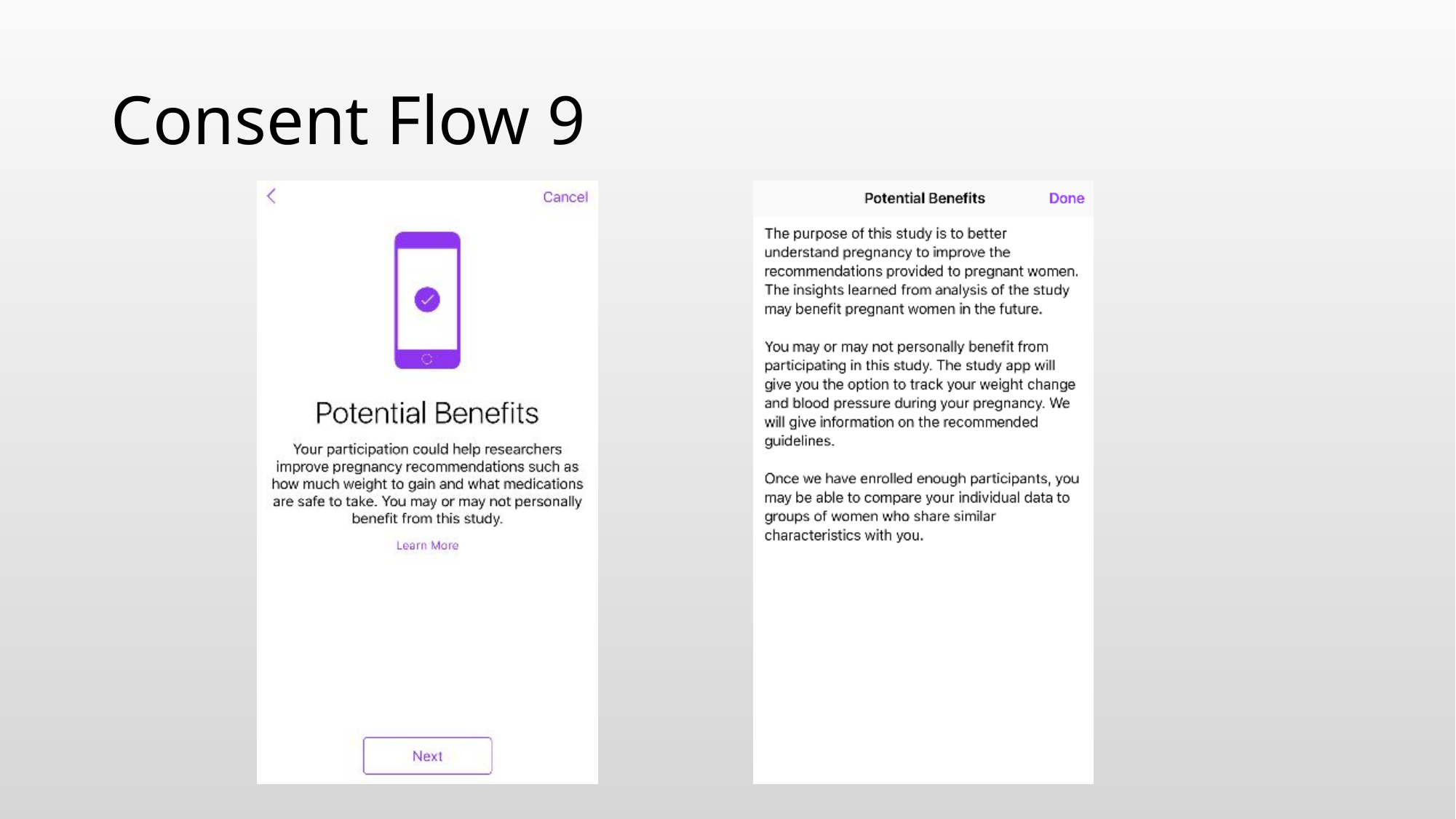

# Consent Flow 9

## Slide 12
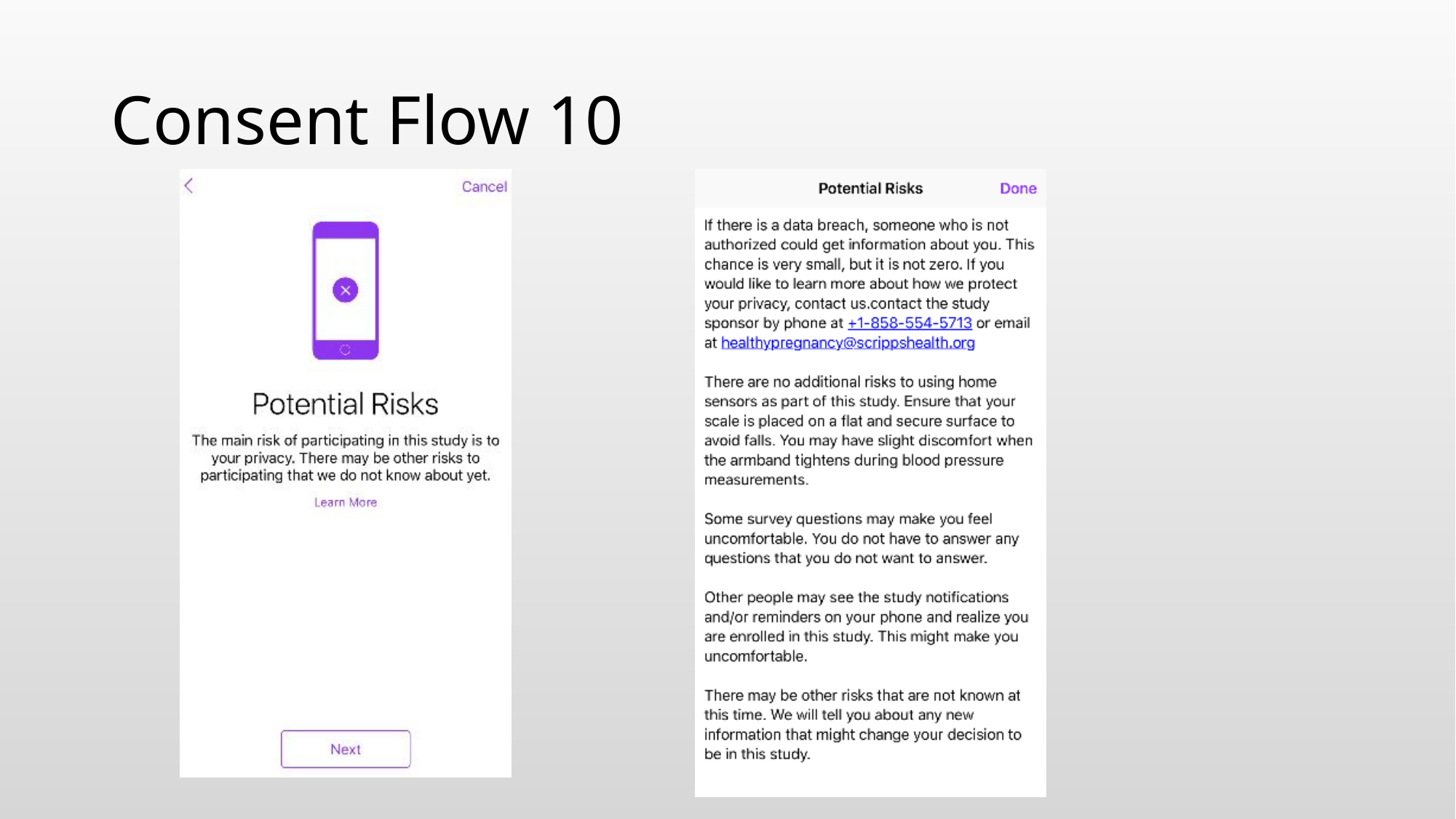

# Consent Flow 10

## Slide 13
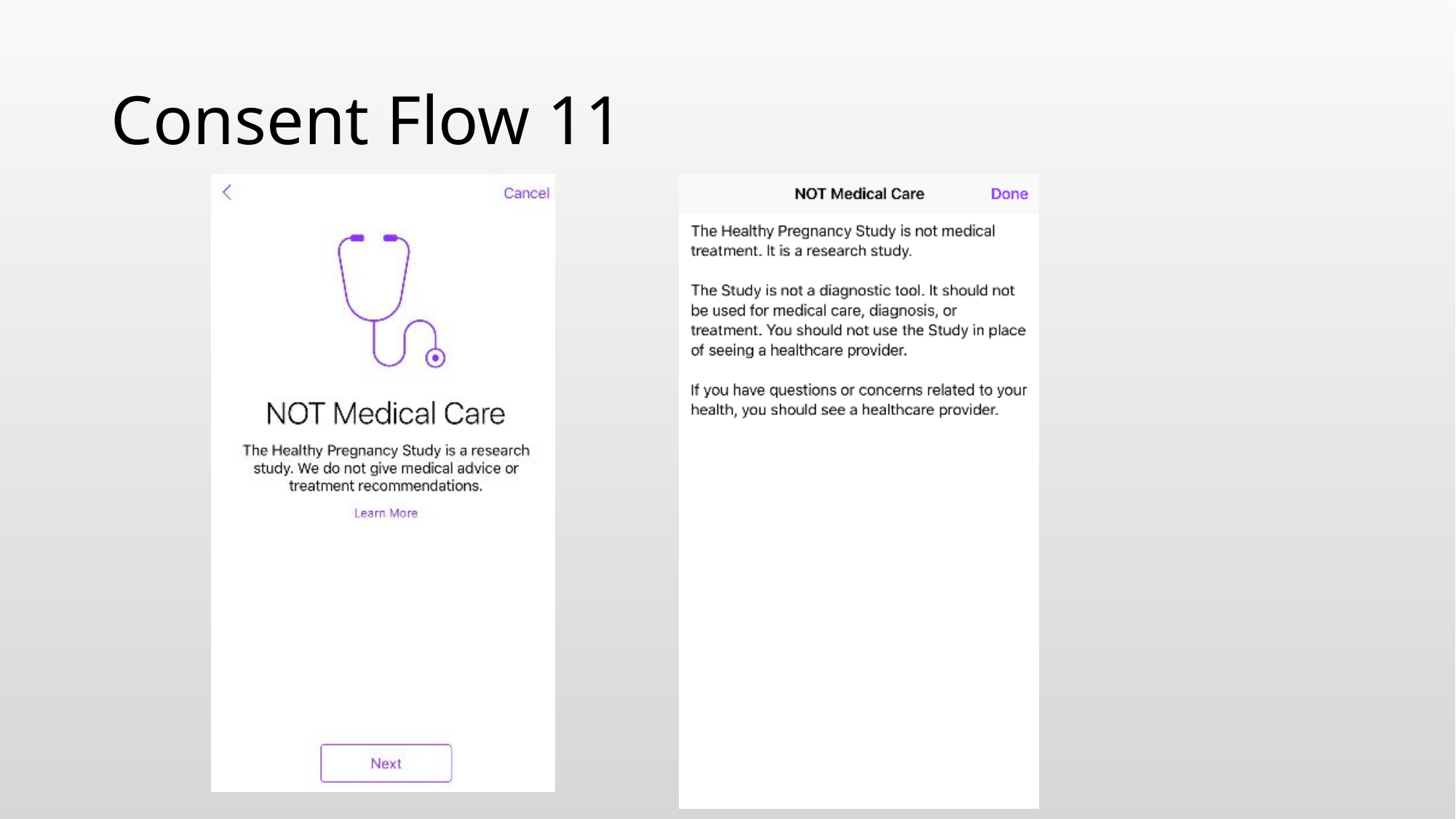

# Consent Flow 11

## Slide 14
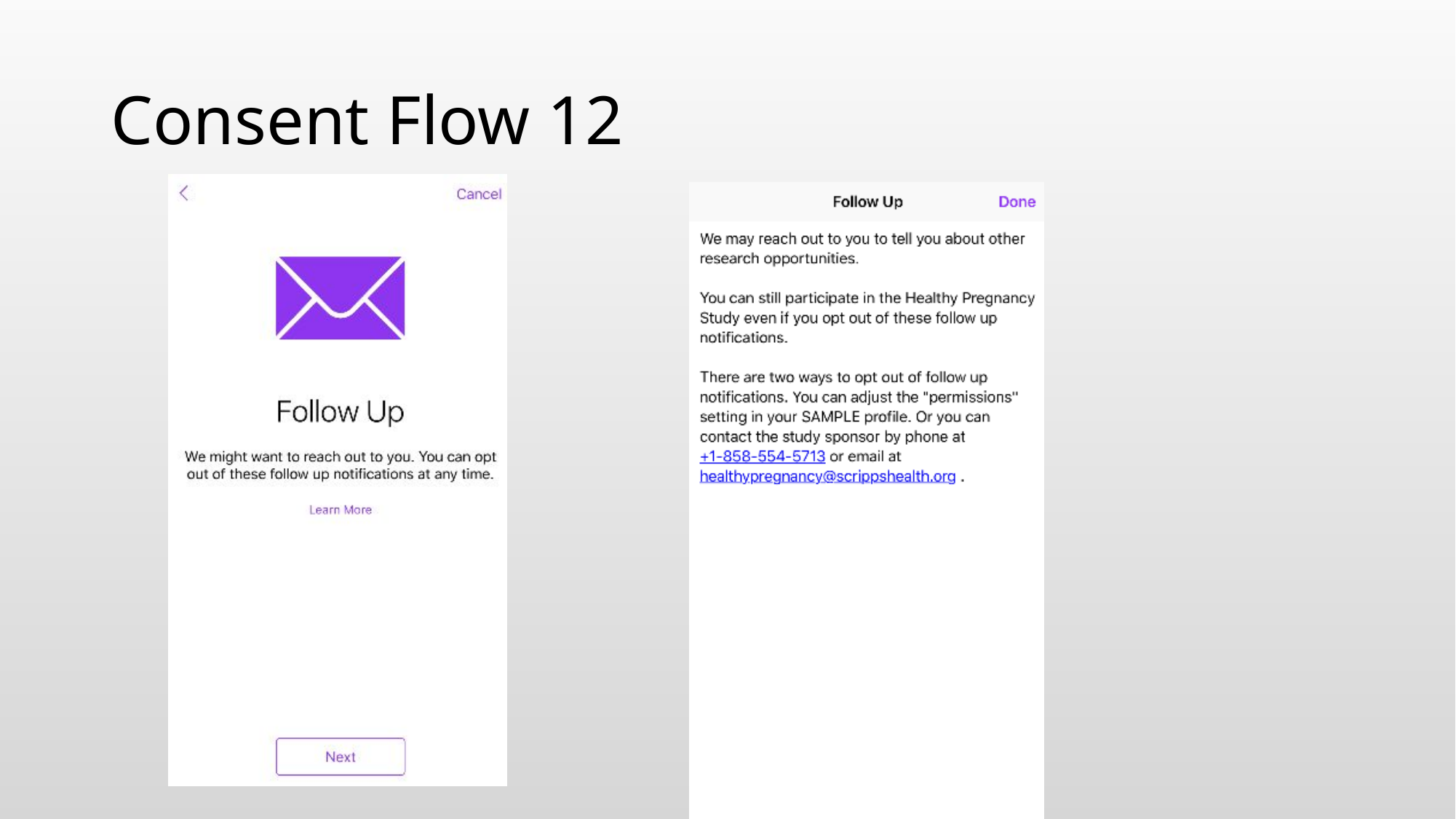

# Consent Flow 12

## Slide 15
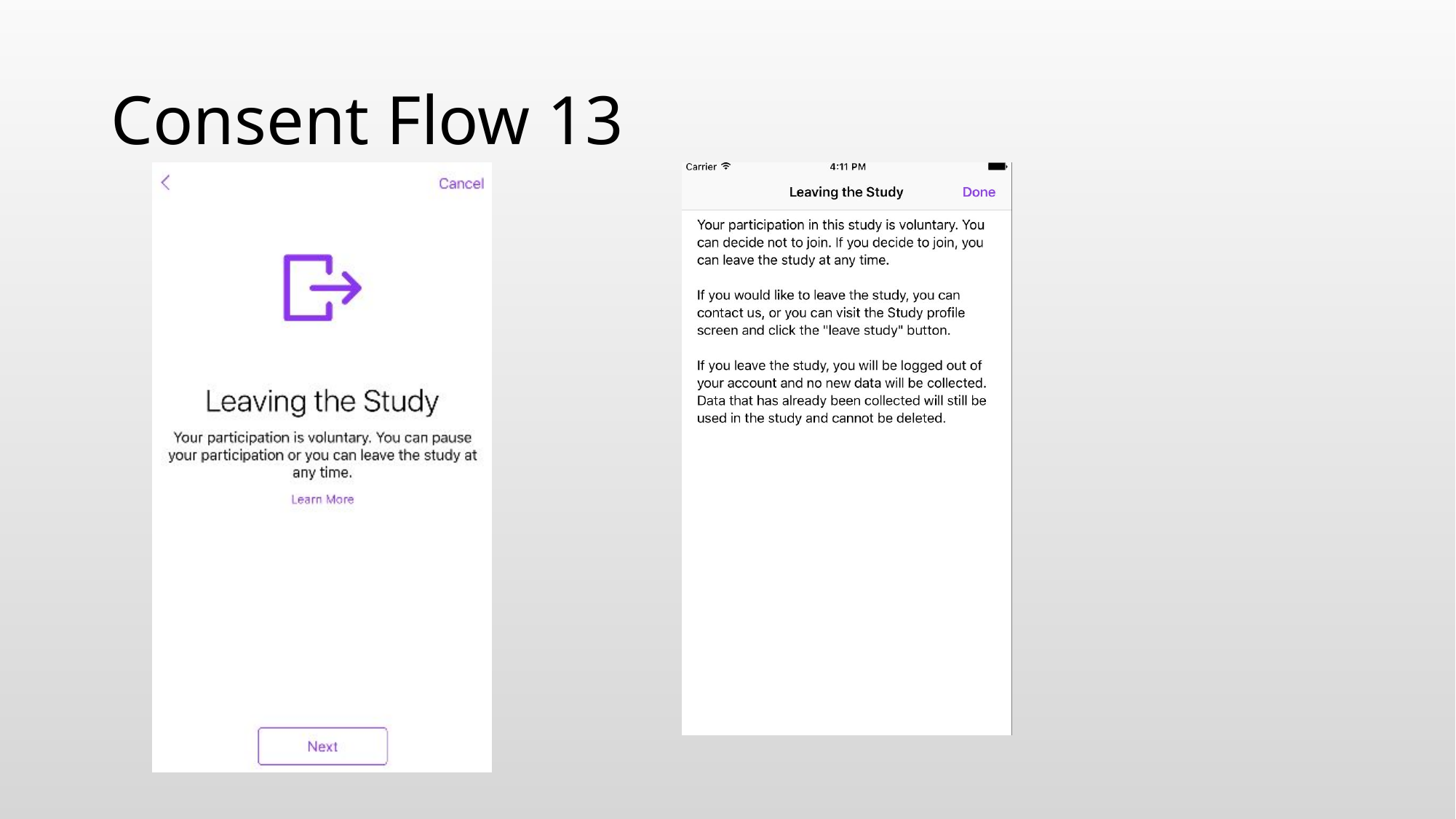

# Consent Flow 13

## Slide 16
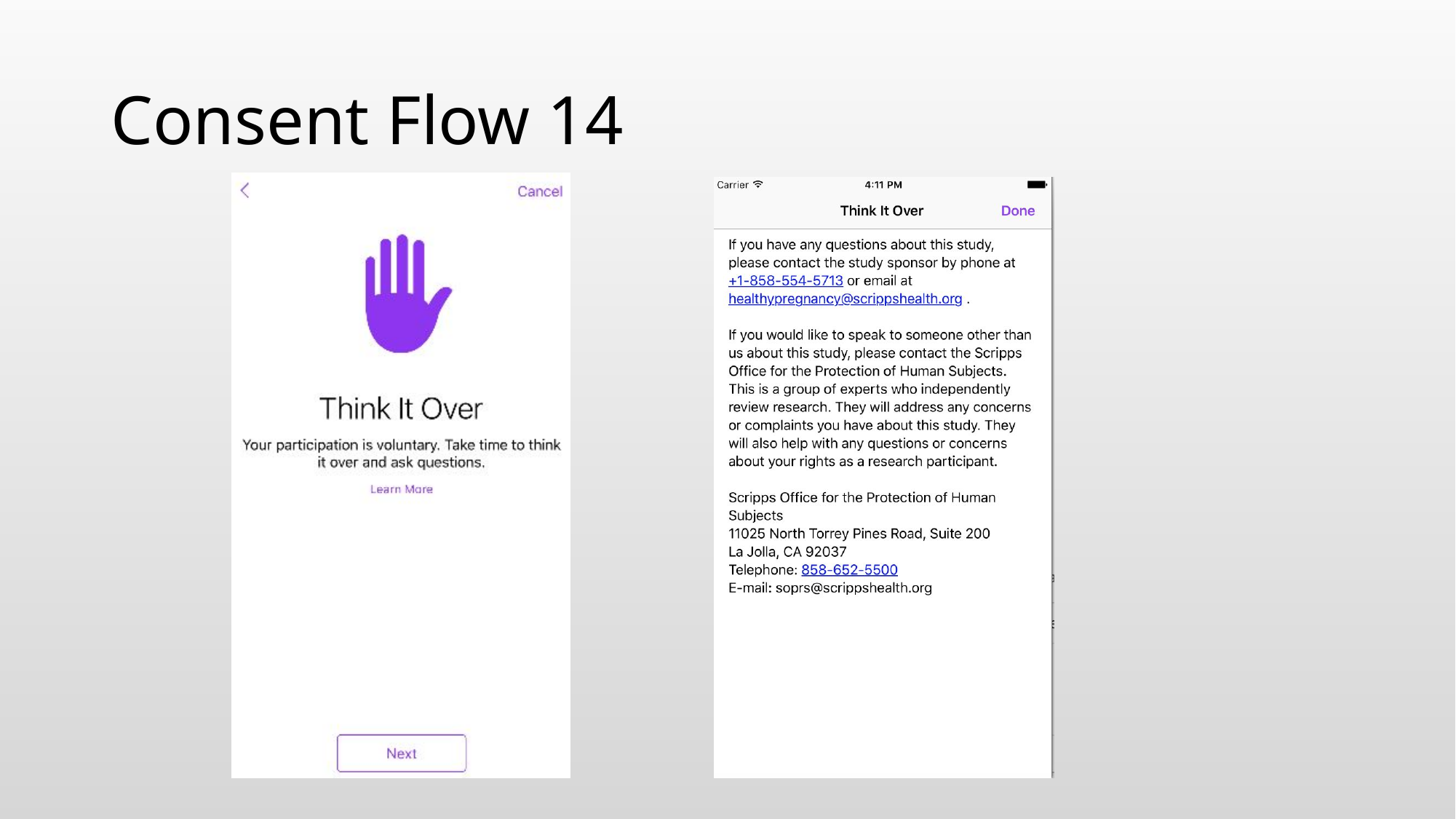

# Consent Flow 14

## Slide 17
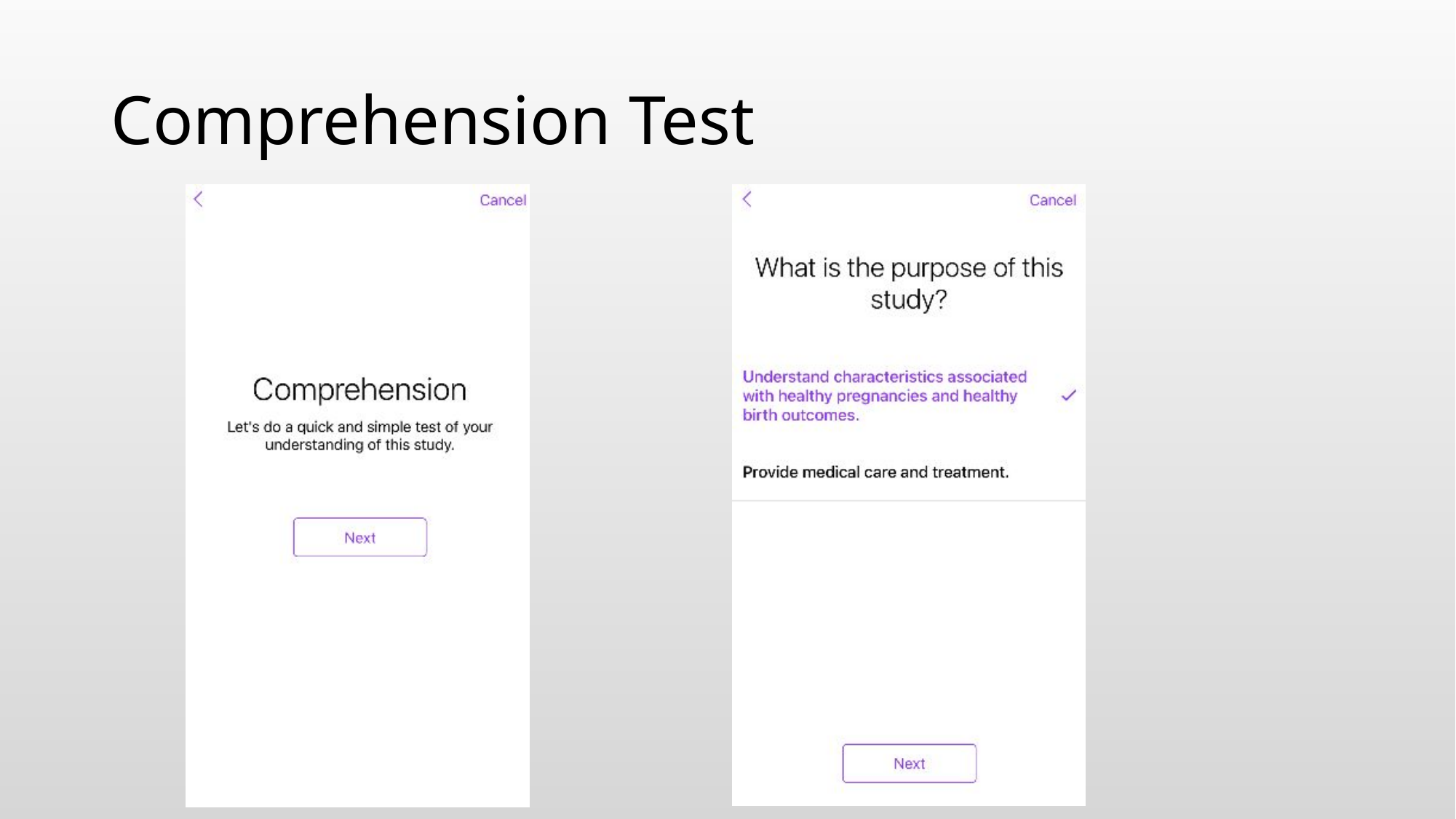

# Comprehension Test

## Slide 18
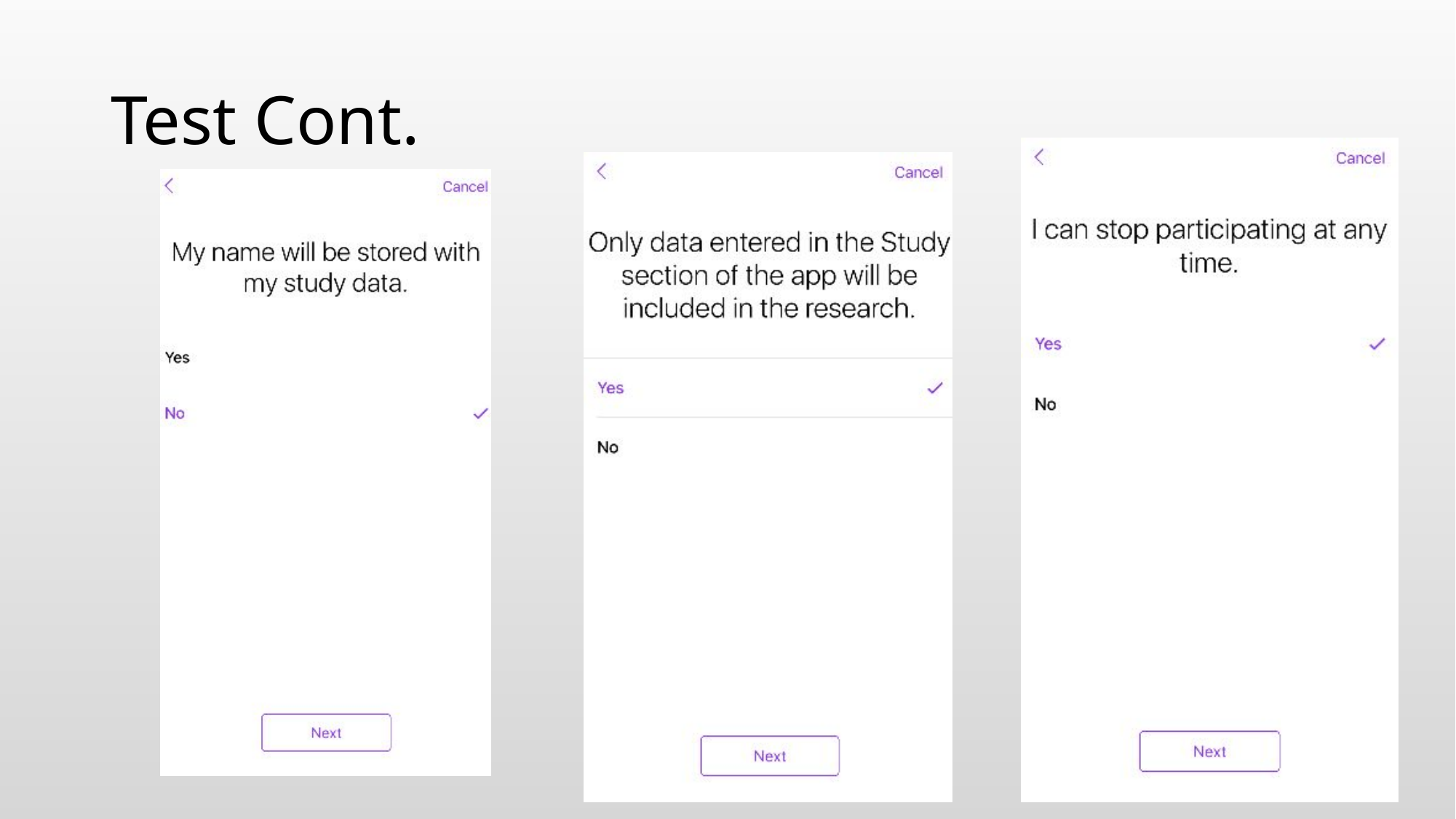

# Test Cont.

## Slide 19
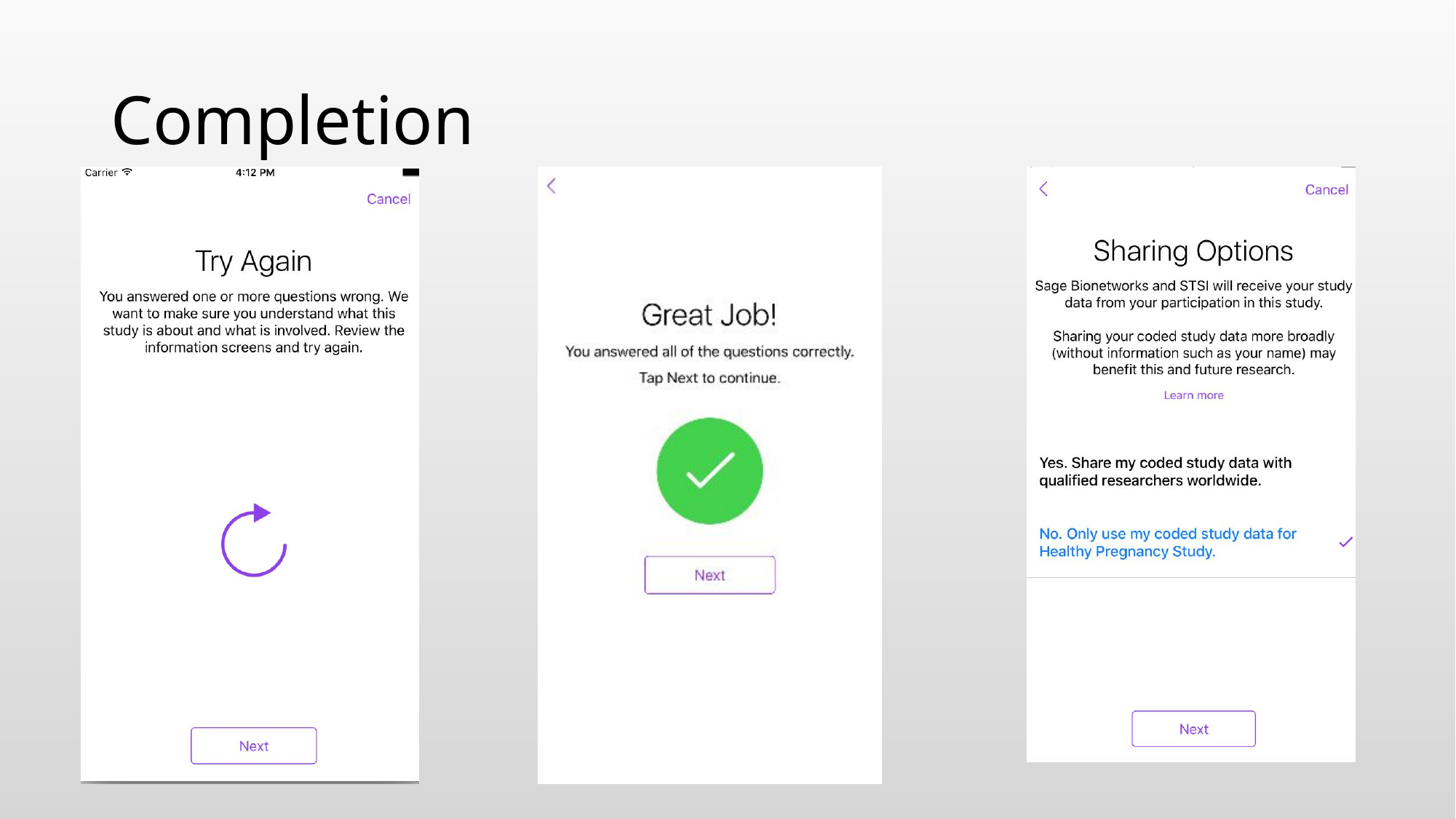

# Completion

## Slide 20
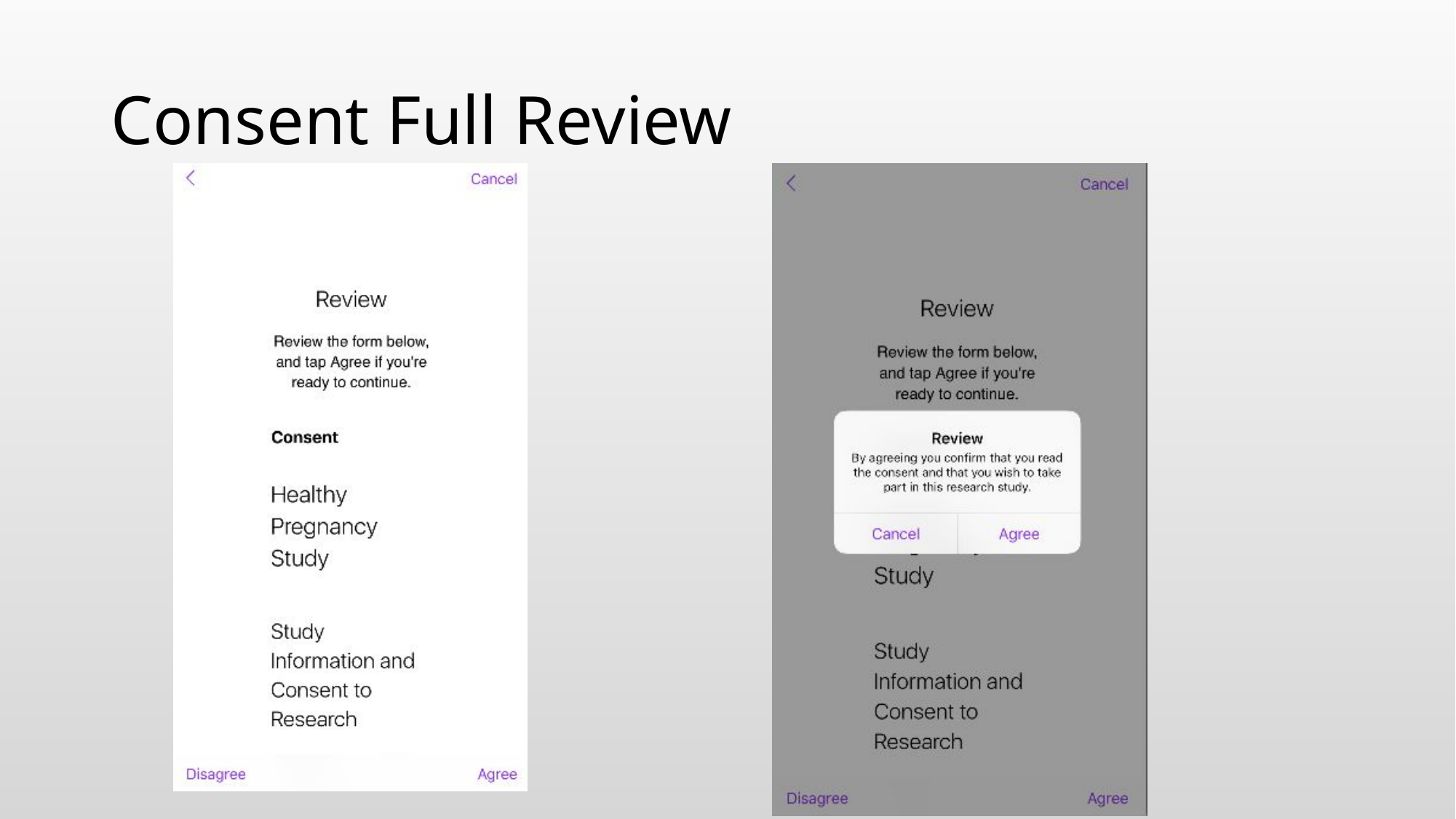

# Consent Full Review

## Slide 21
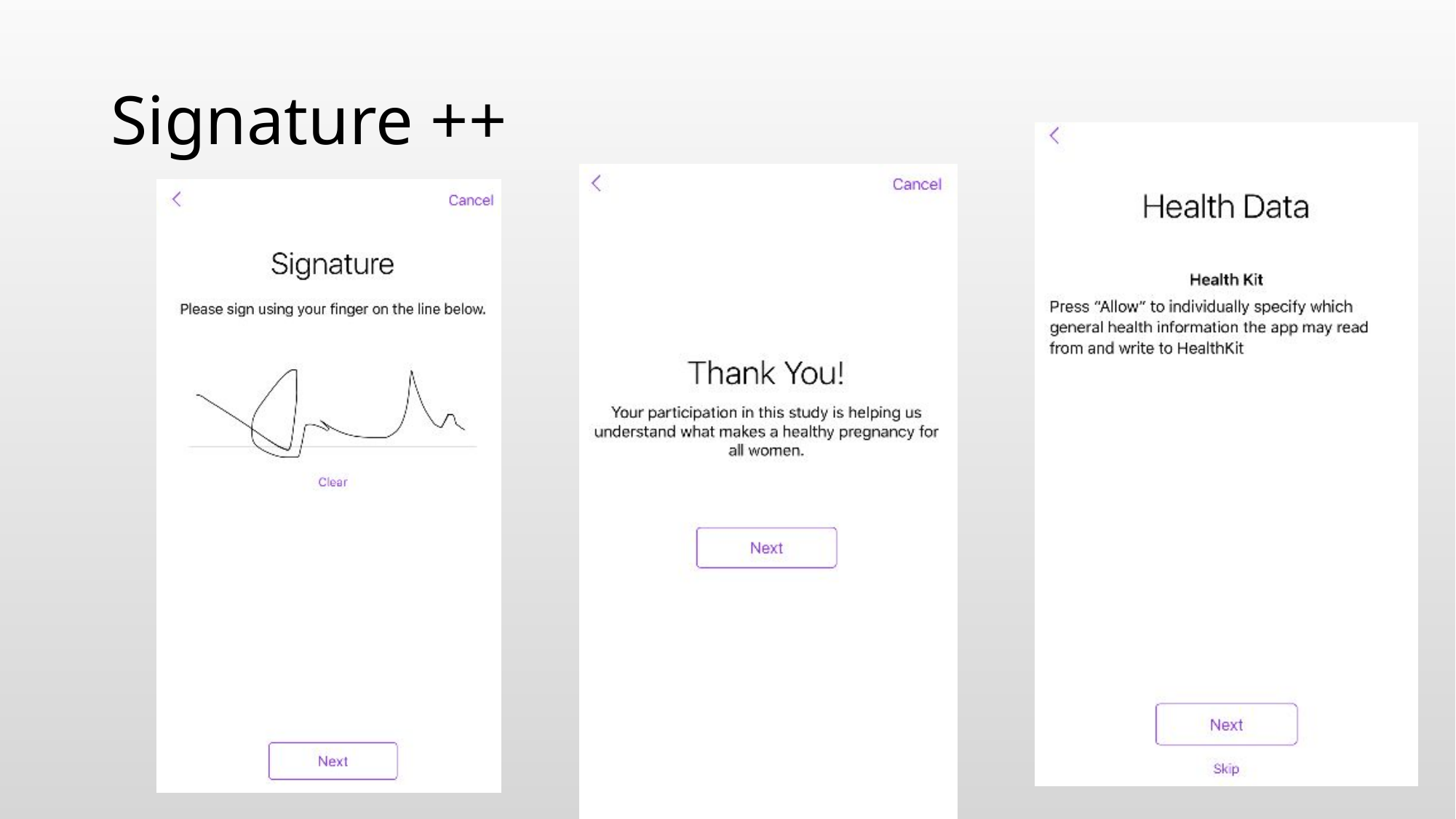

# Signature ++

## Slide 22
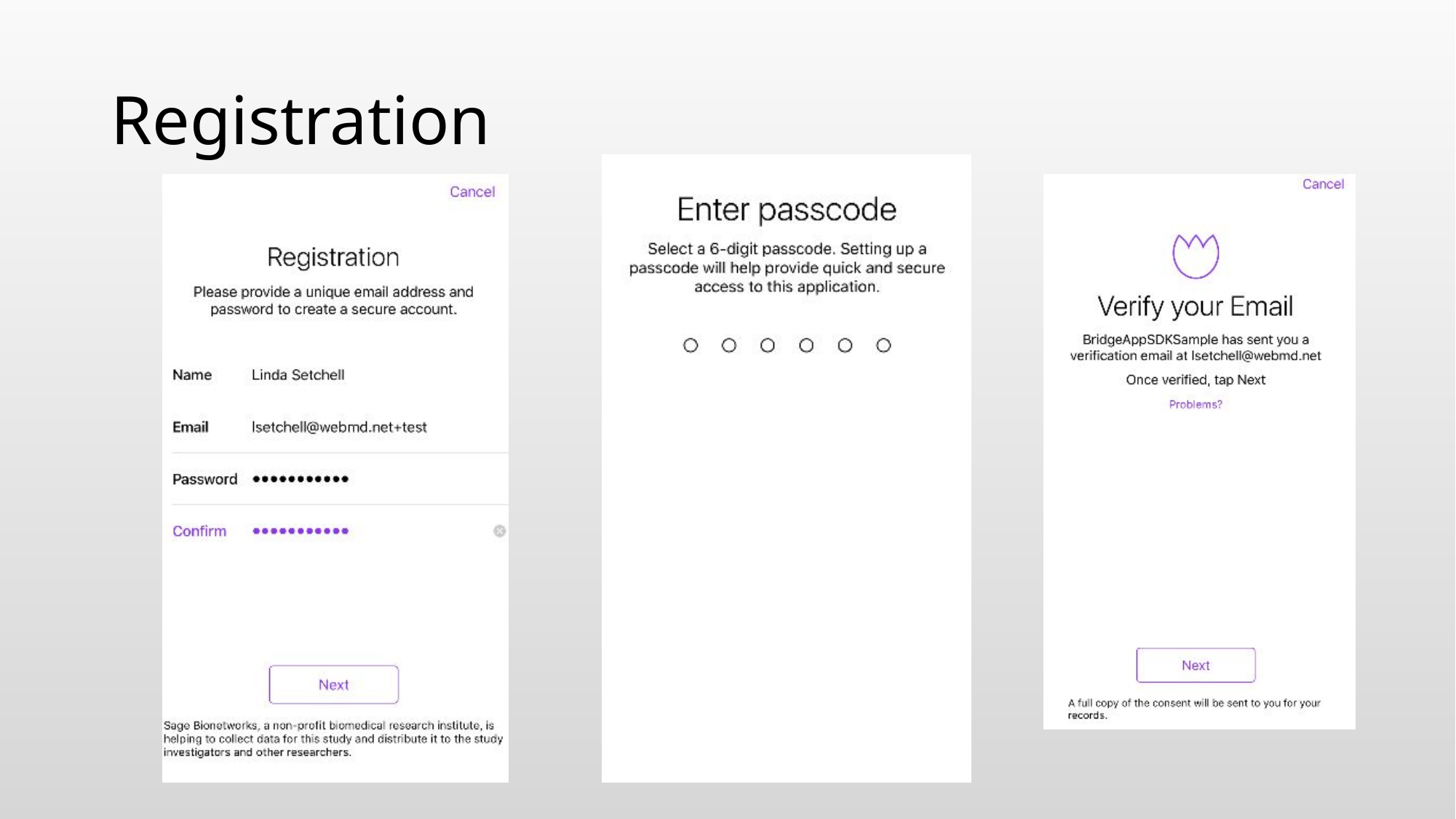

# Registration

## Slide 23
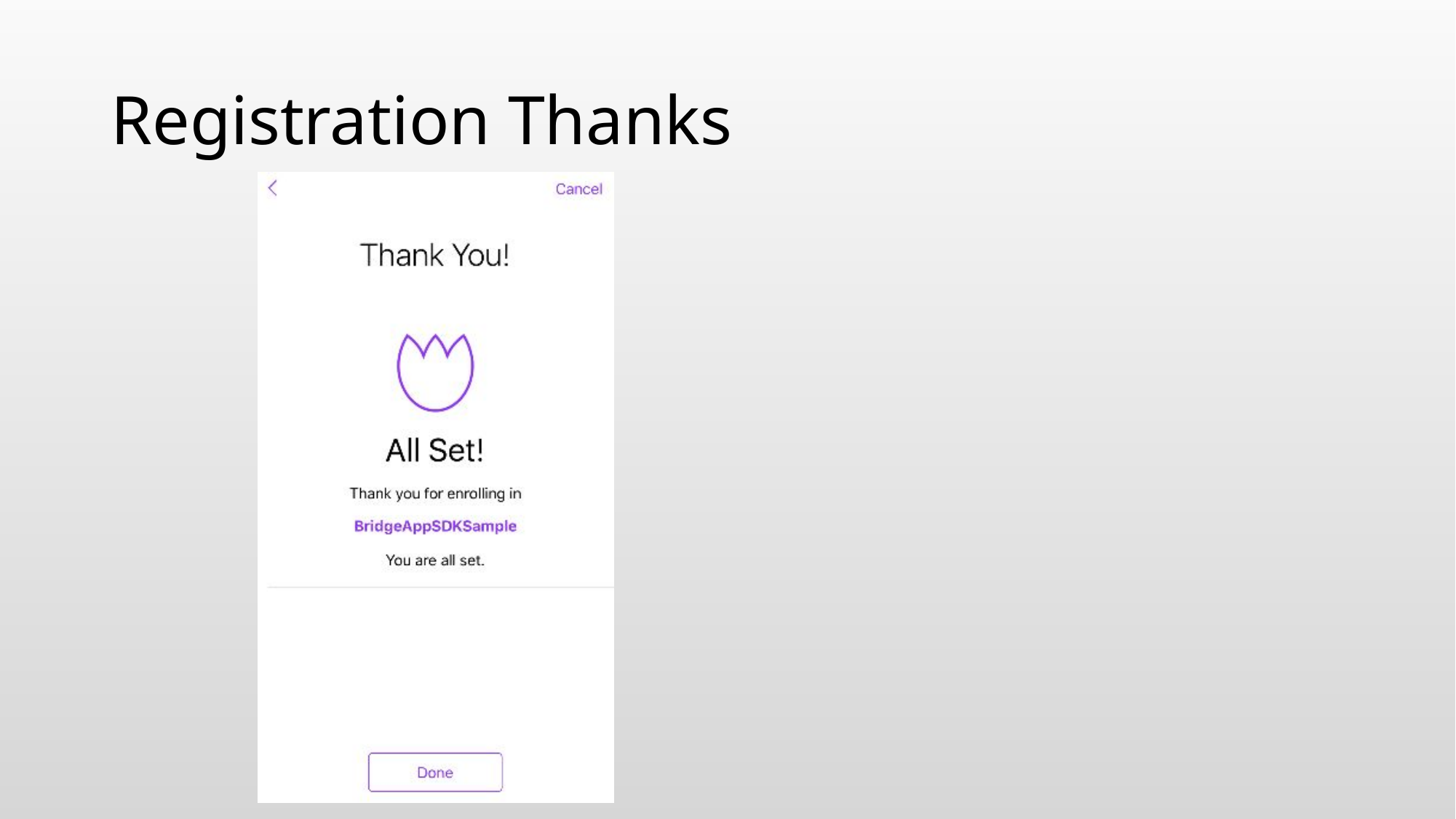

# Registration Thanks

## Slide 24
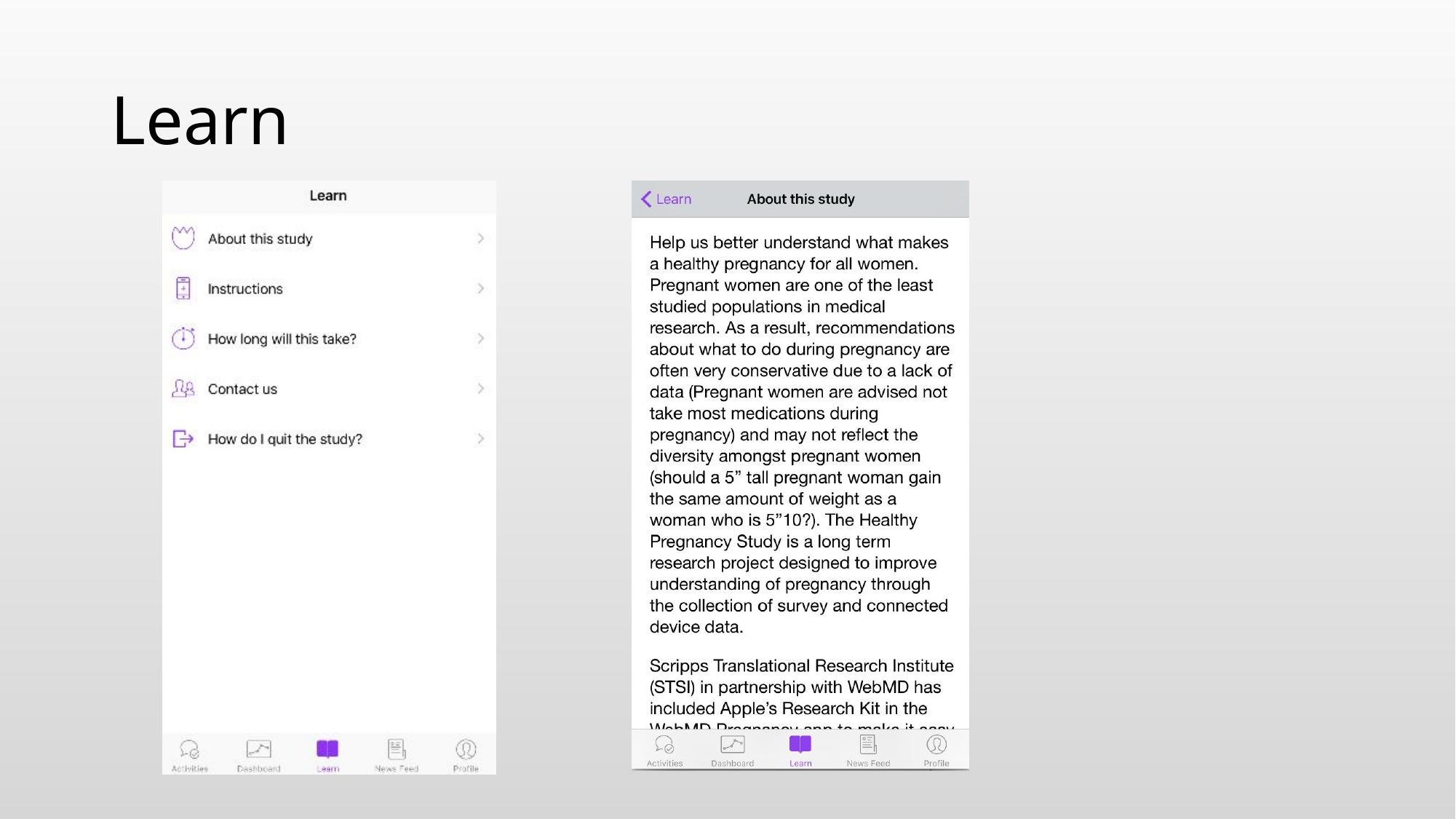

# Learn

## Slide 25
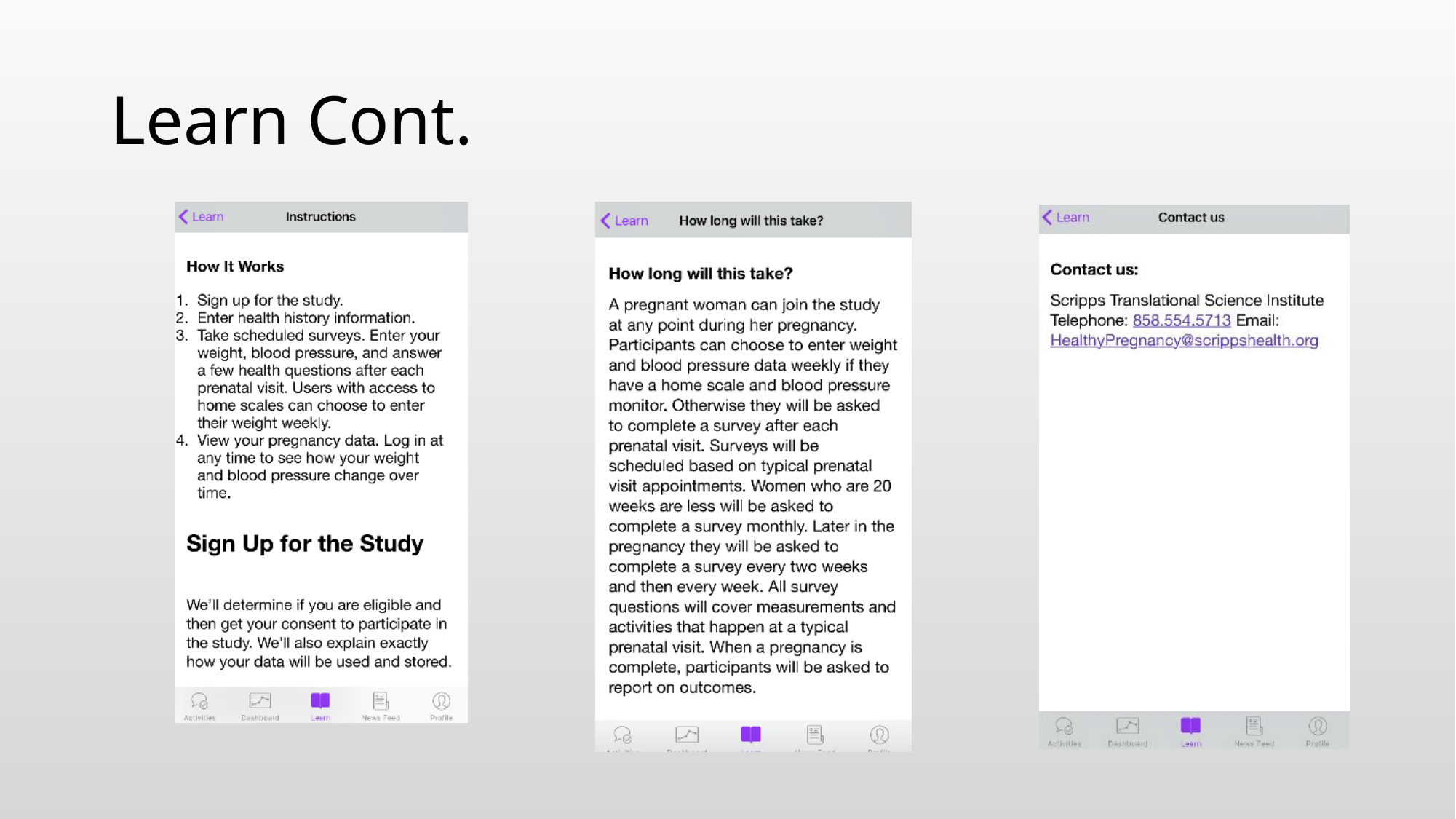

# Learn Cont.

## Slide 26
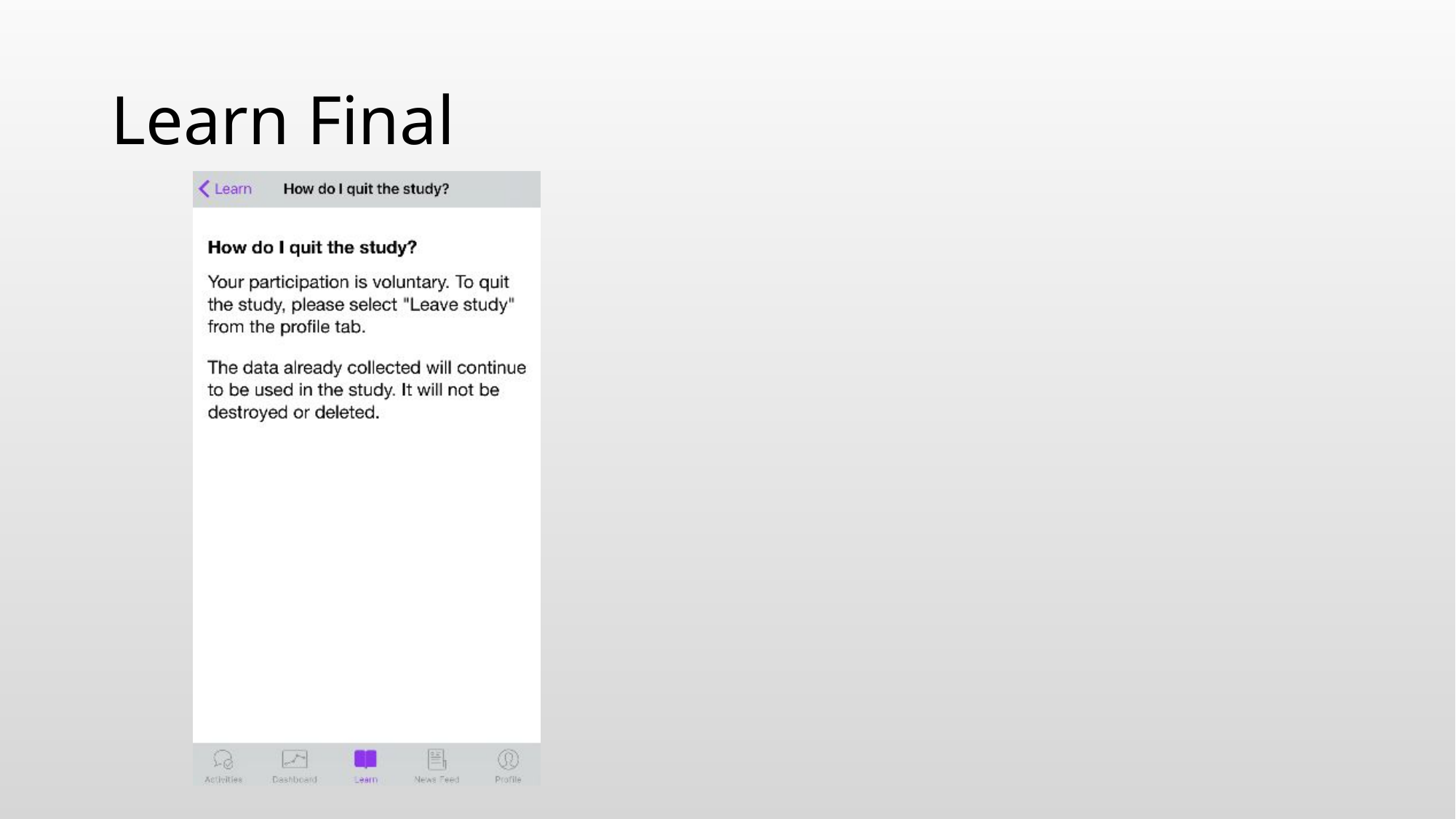

# Learn Final
