# Supplementary material for: Self-Reported Medication Use Across Racial and Rural or Urban Subgroups of People Who Are Pregnant in the United States: Decentralized App-Based Cohort Study
Source: JMIR Form Res. 2023 Nov 28;7:e50867. doi: 10.2196/50867 (PMC10716764; doi:10.2196/50867)
Supplement: Multimedia Appendix 2 [file formative_v7i1e50867_app2.pptx]

## Slide 1
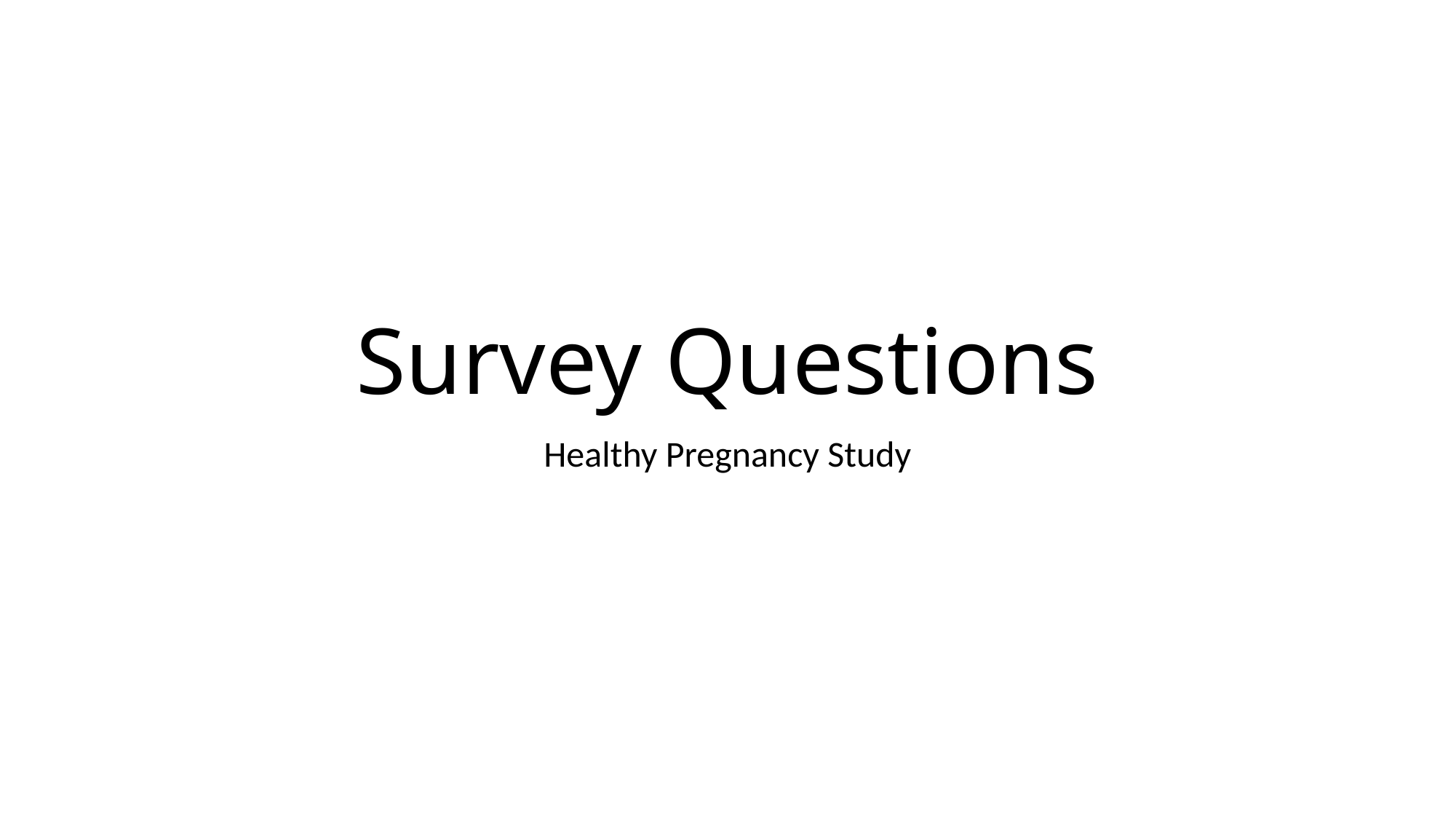

# Survey Questions
Healthy Pregnancy Study

## Slide 2
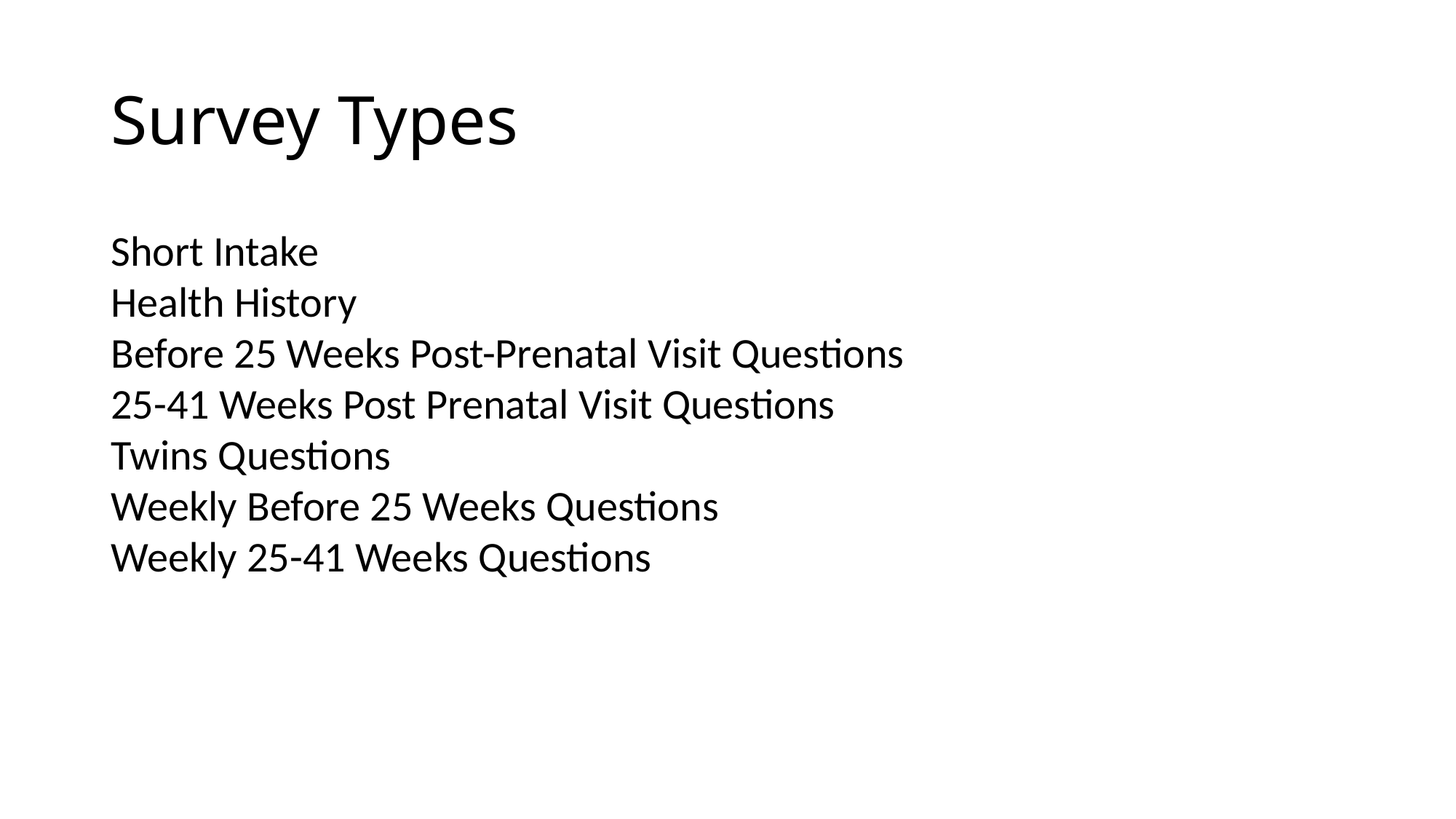

# Survey Types
Short Intake
Health History
Before 25 Weeks Post-Prenatal Visit Questions
25-41 Weeks Post Prenatal Visit Questions
Twins Questions
Weekly Before 25 Weeks Questions
Weekly 25-41 Weeks Questions

## Slide 3
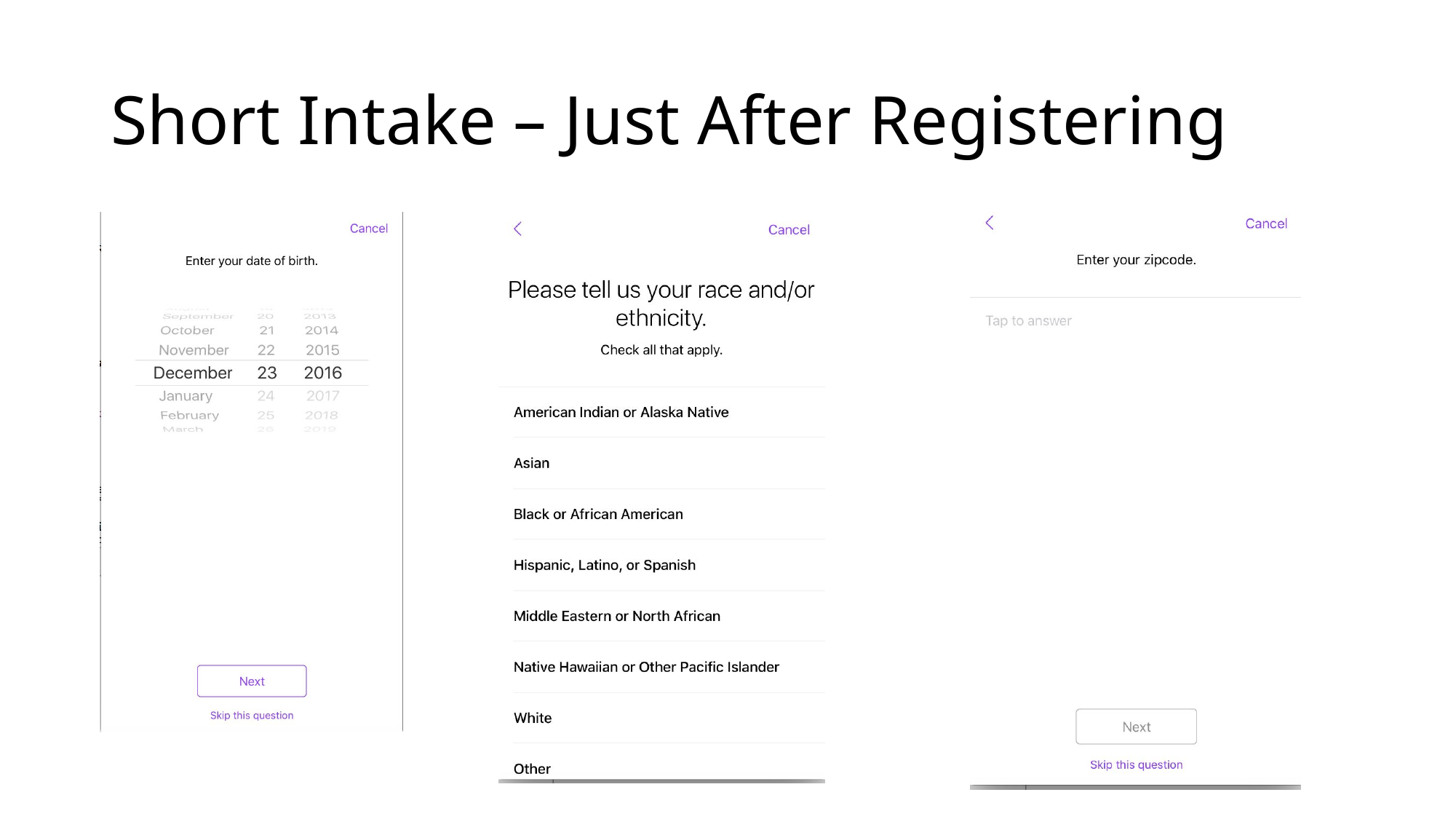

# Short Intake – Just After Registering

## Slide 4
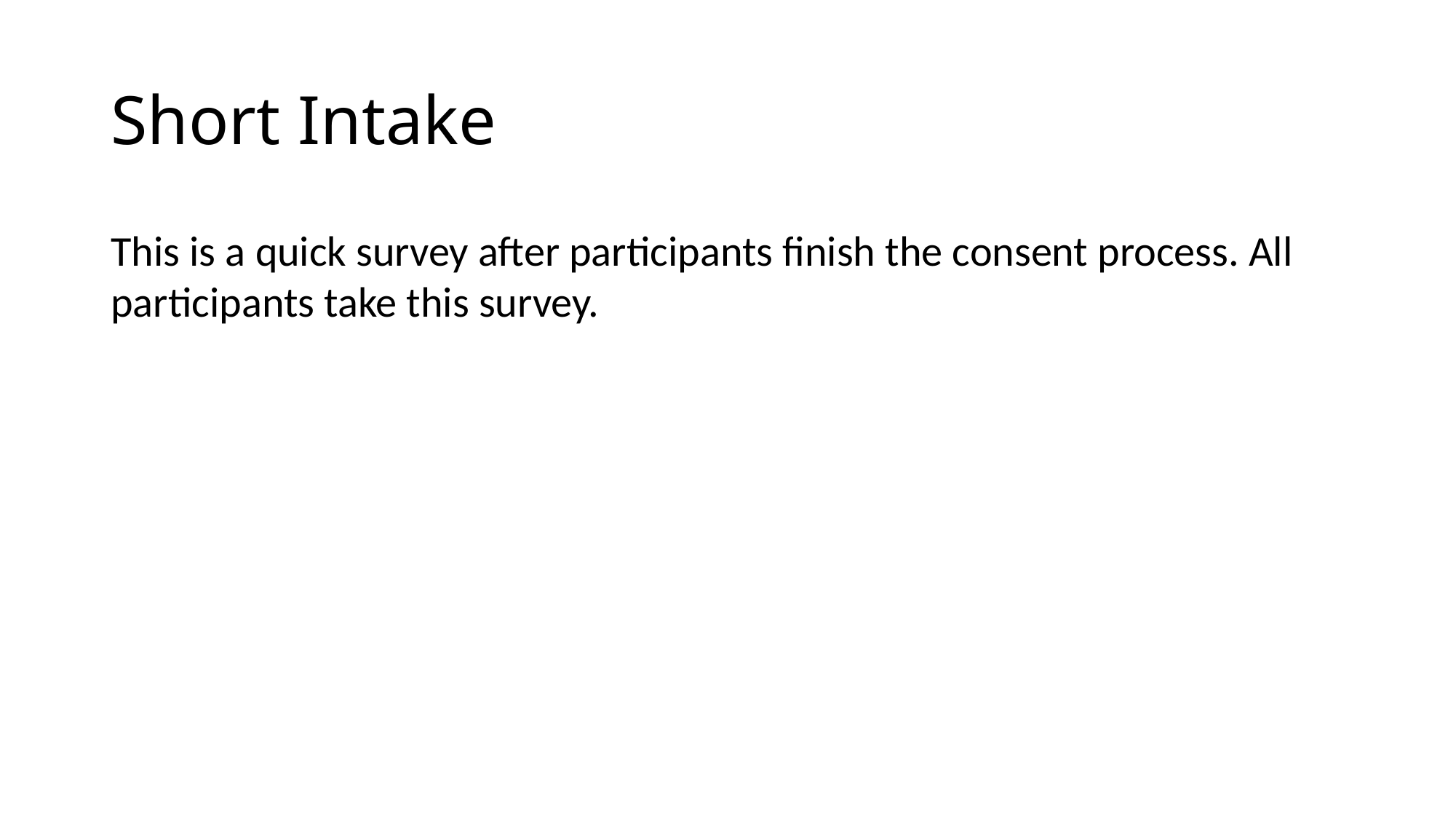

# Short Intake
This is a quick survey after participants finish the consent process. All participants take this survey.

## Slide 5
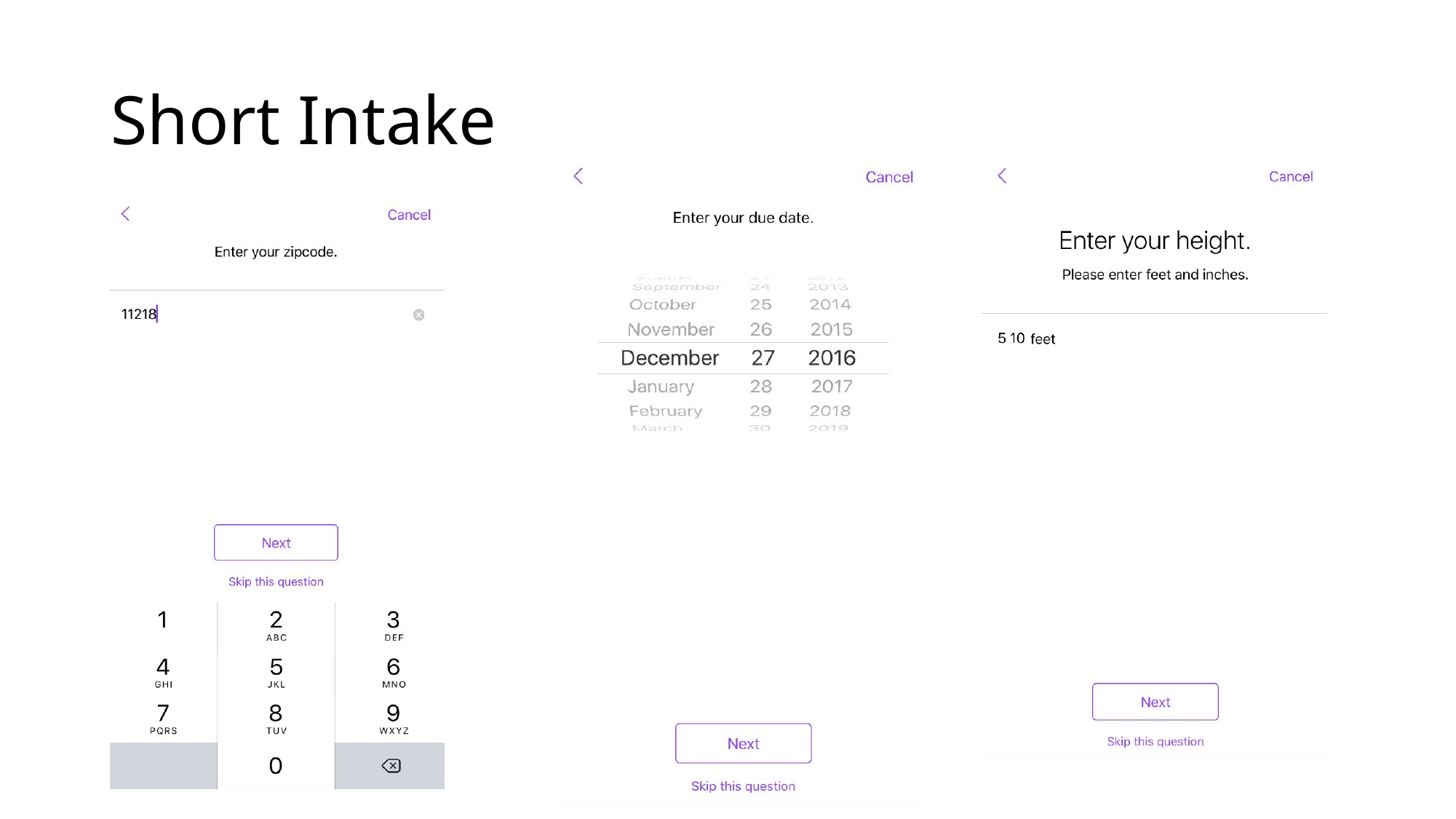

# Short Intake

## Slide 6
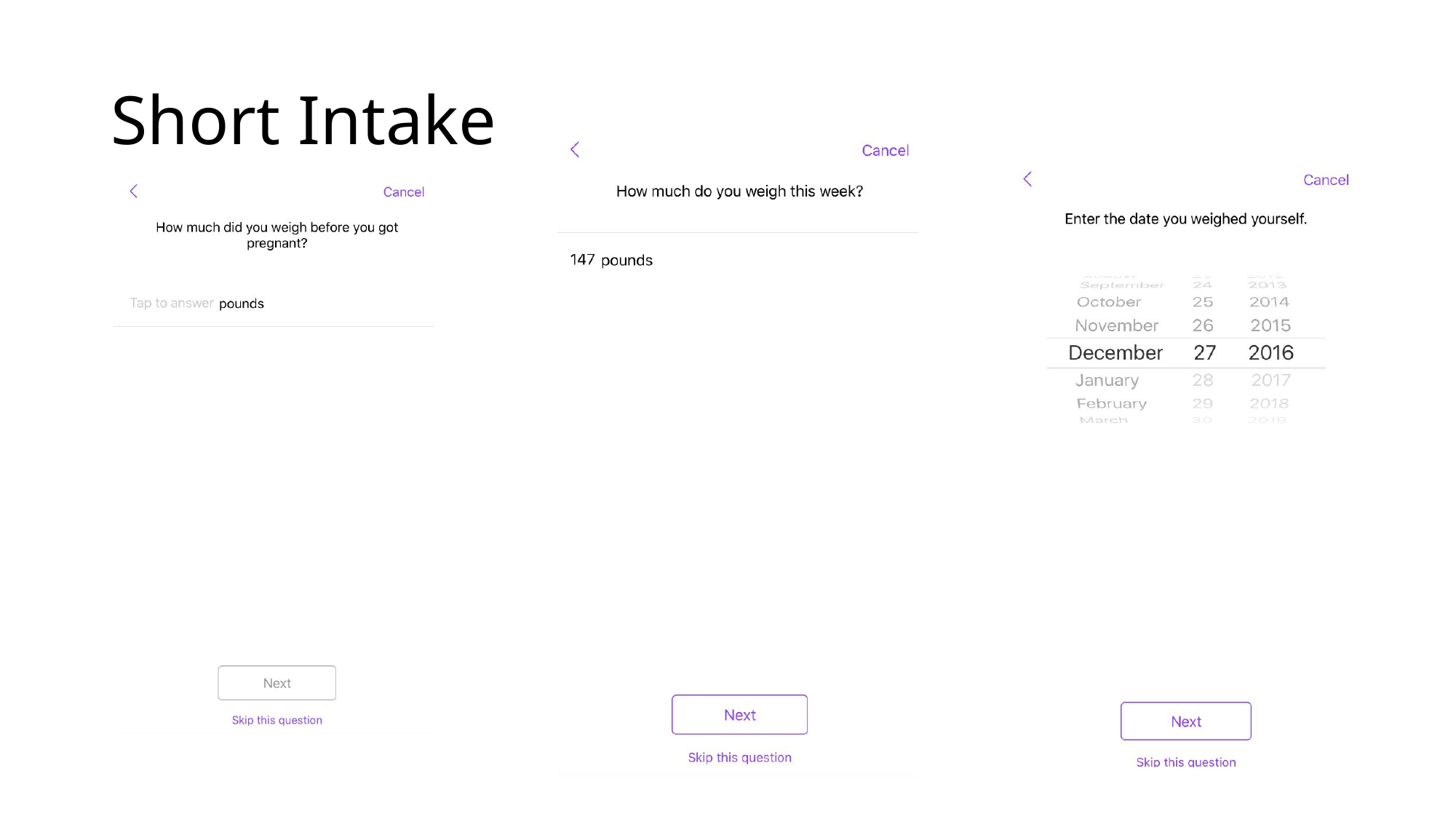

# Short Intake

## Slide 7
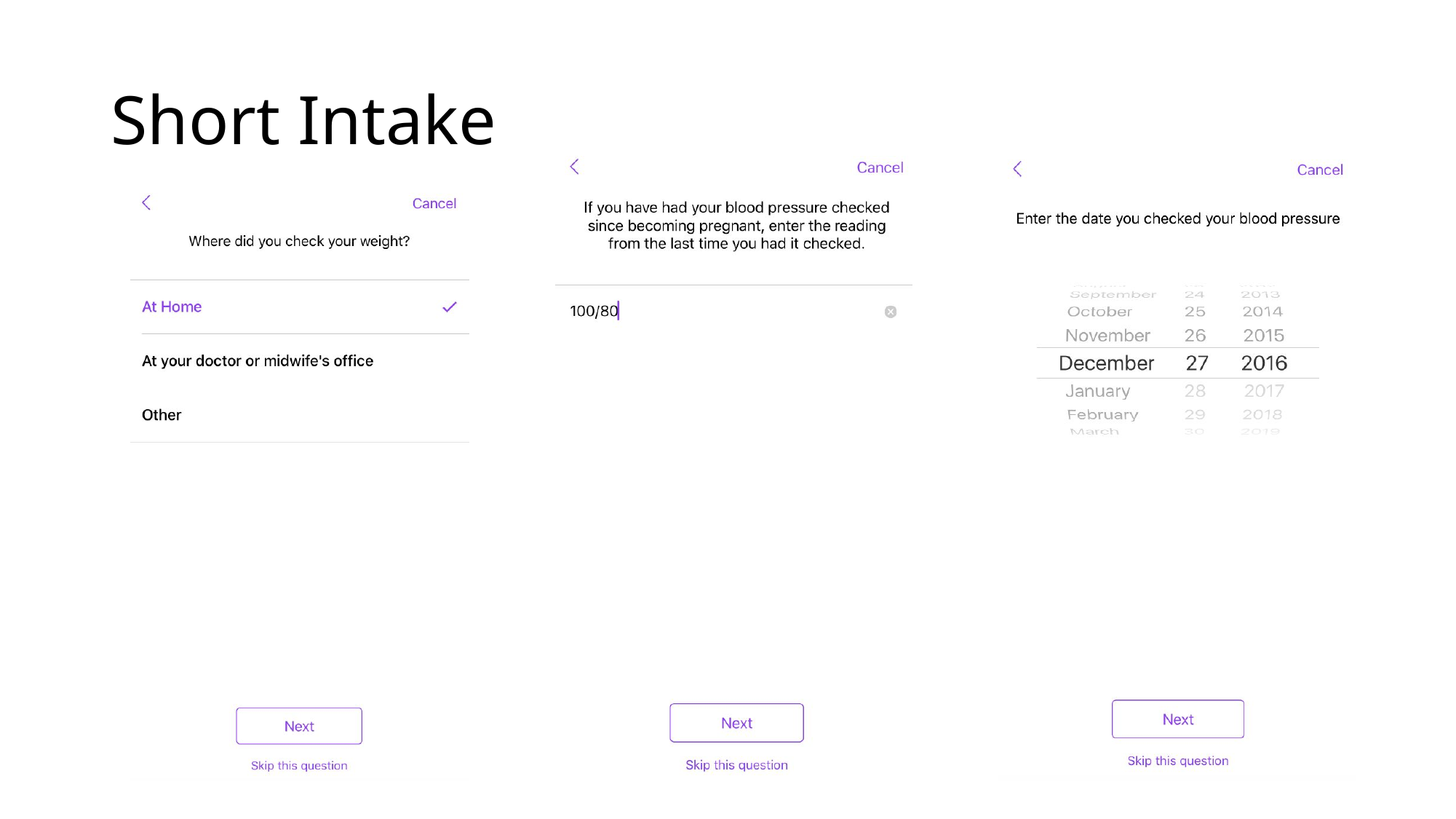

# Short Intake

## Slide 8
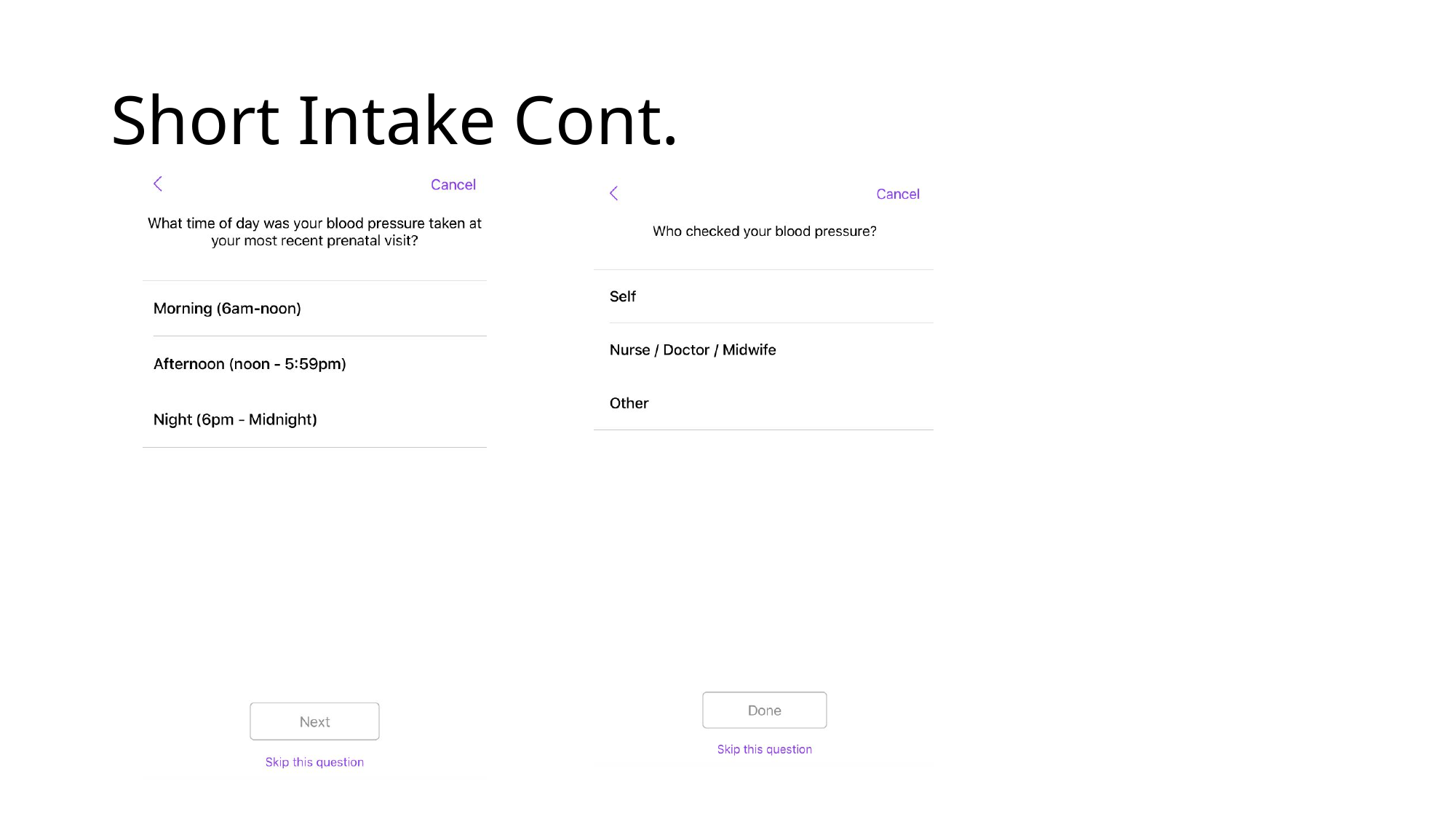

# Short Intake Cont.

## Slide 9
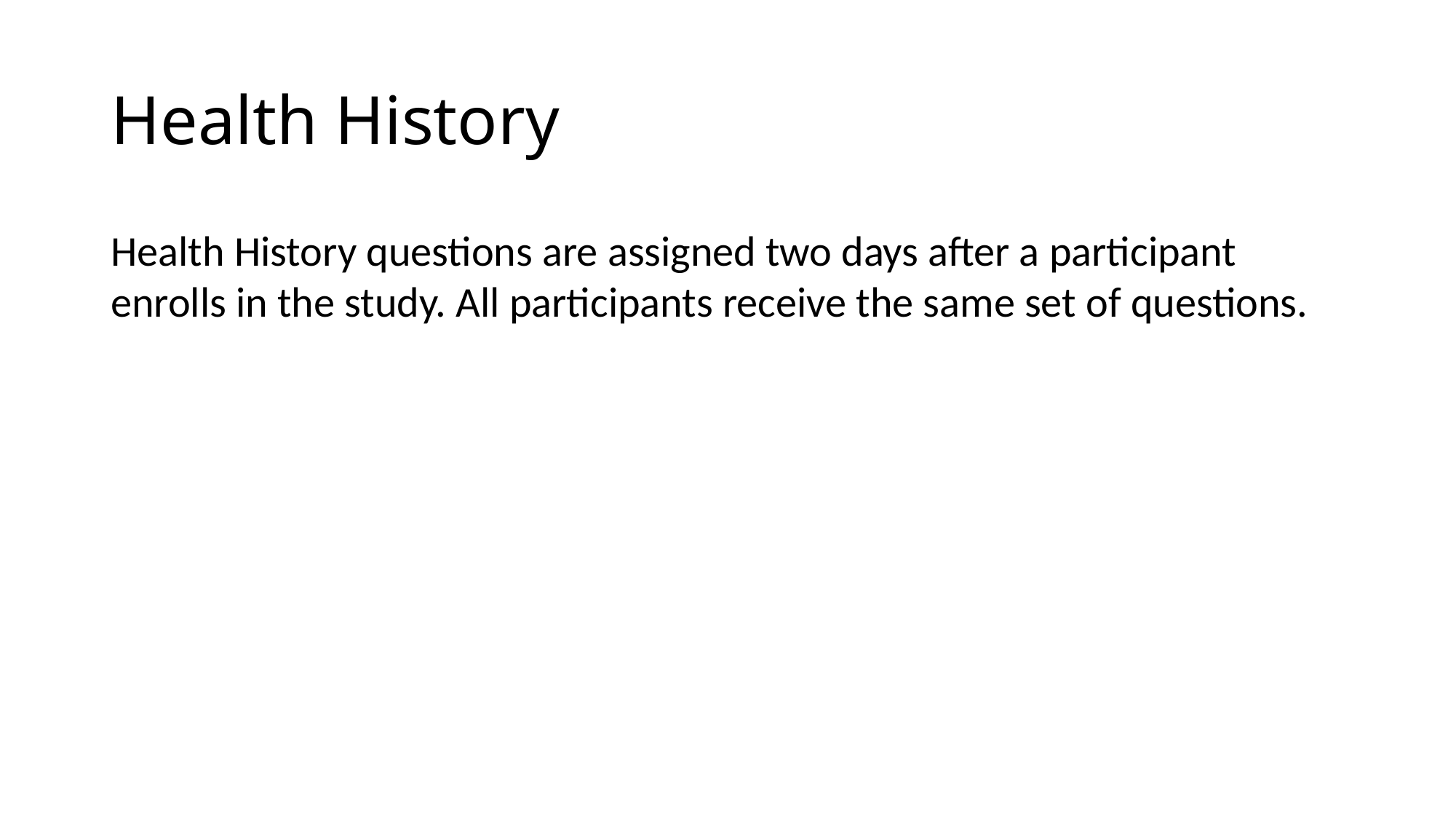

# Health History
Health History questions are assigned two days after a participant enrolls in the study. All participants receive the same set of questions.

## Slide 10
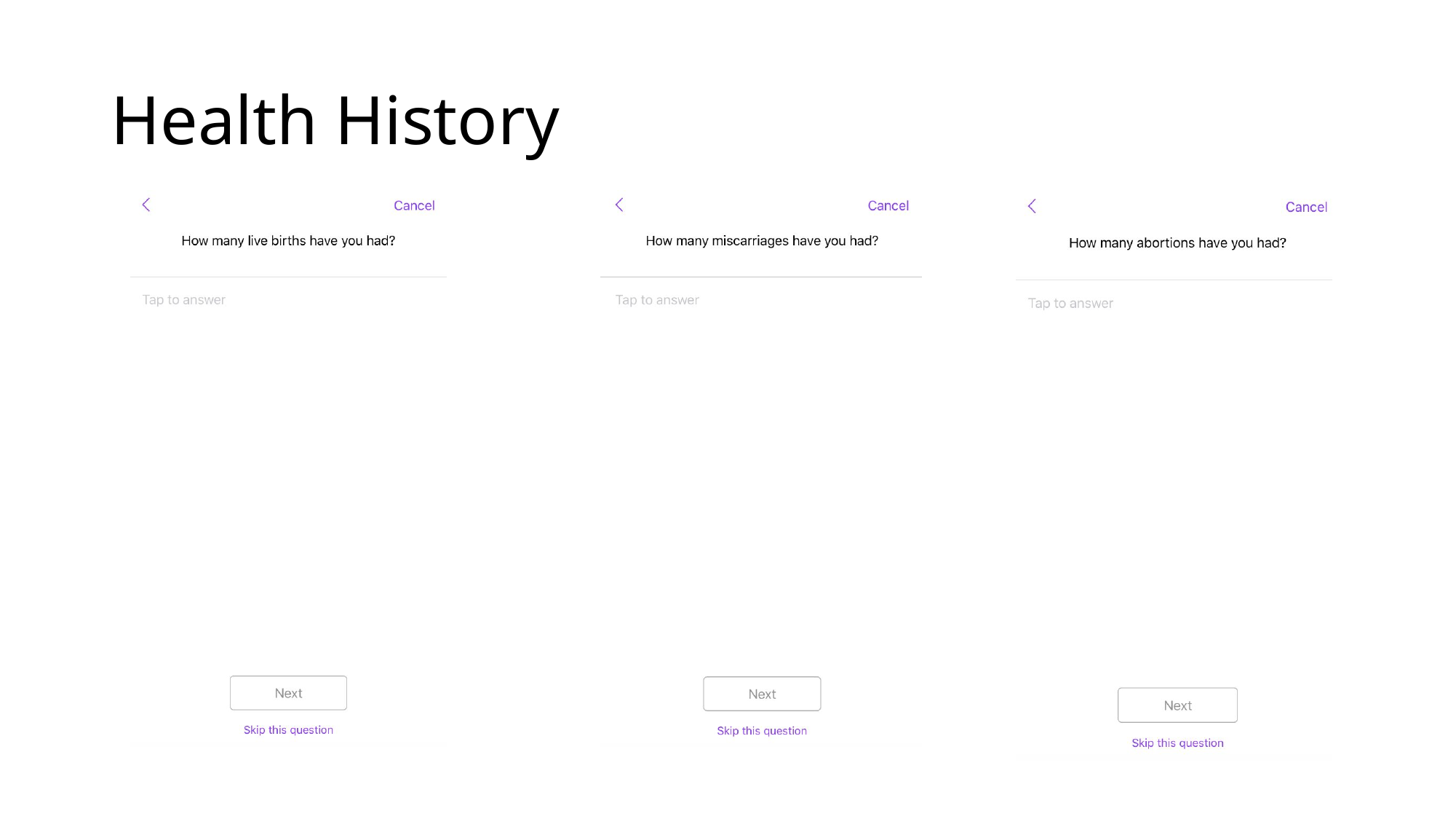

# Health History

## Slide 11
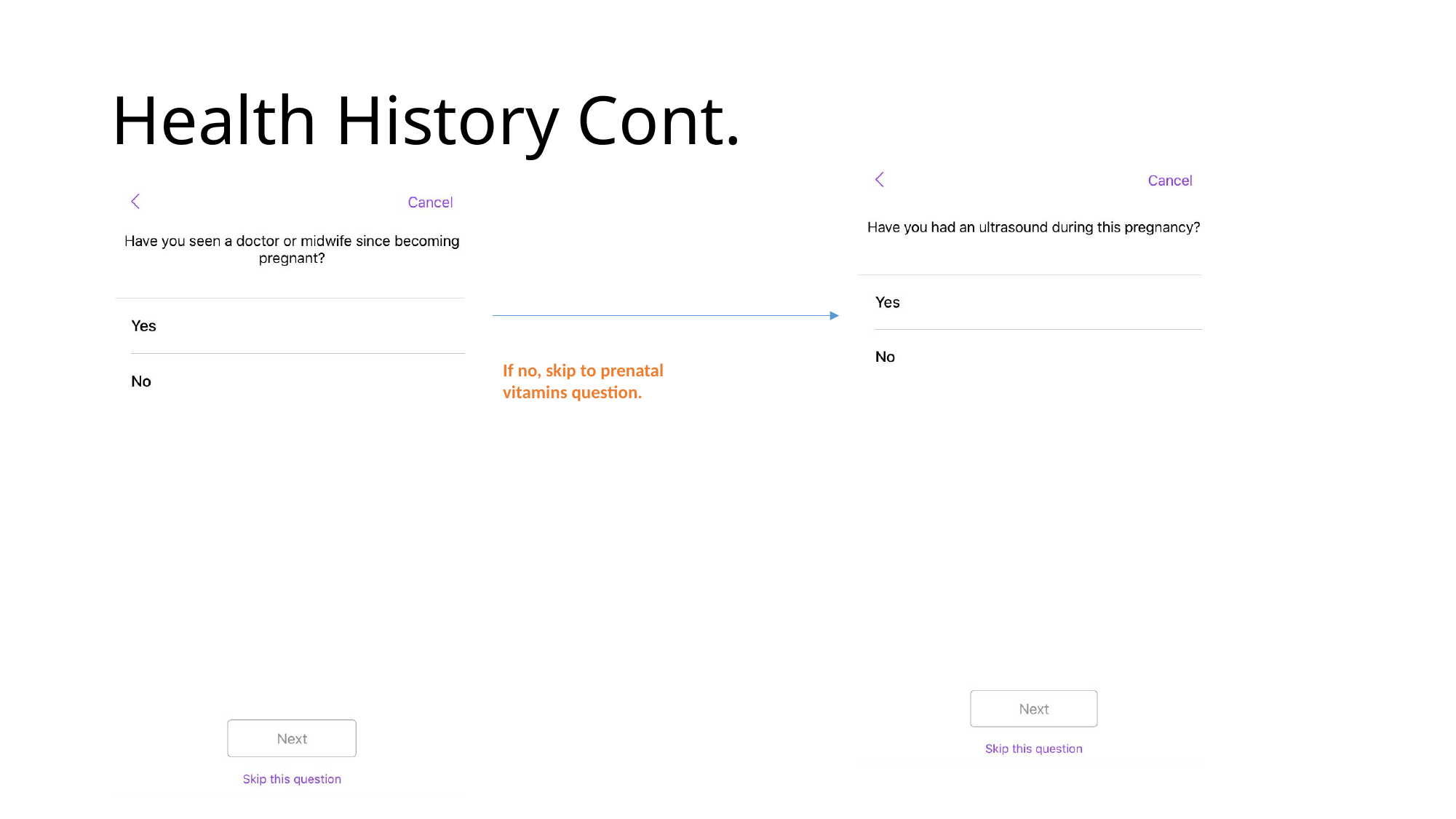

# Health History Cont.
If no, skip to prenatal vitamins question.

## Slide 12
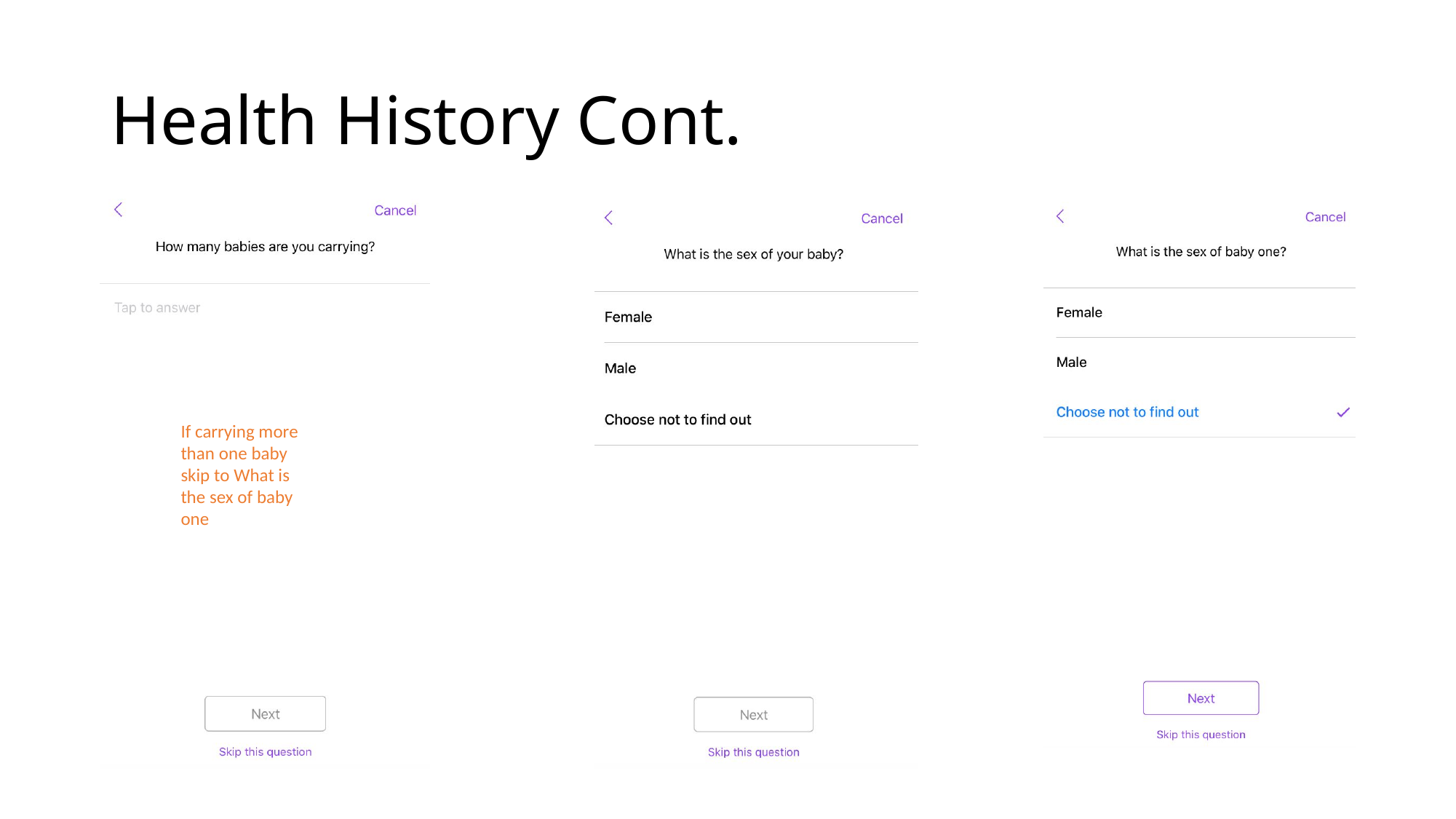

# Health History Cont.
If carrying more than one baby skip to What is the sex of baby one

## Slide 13
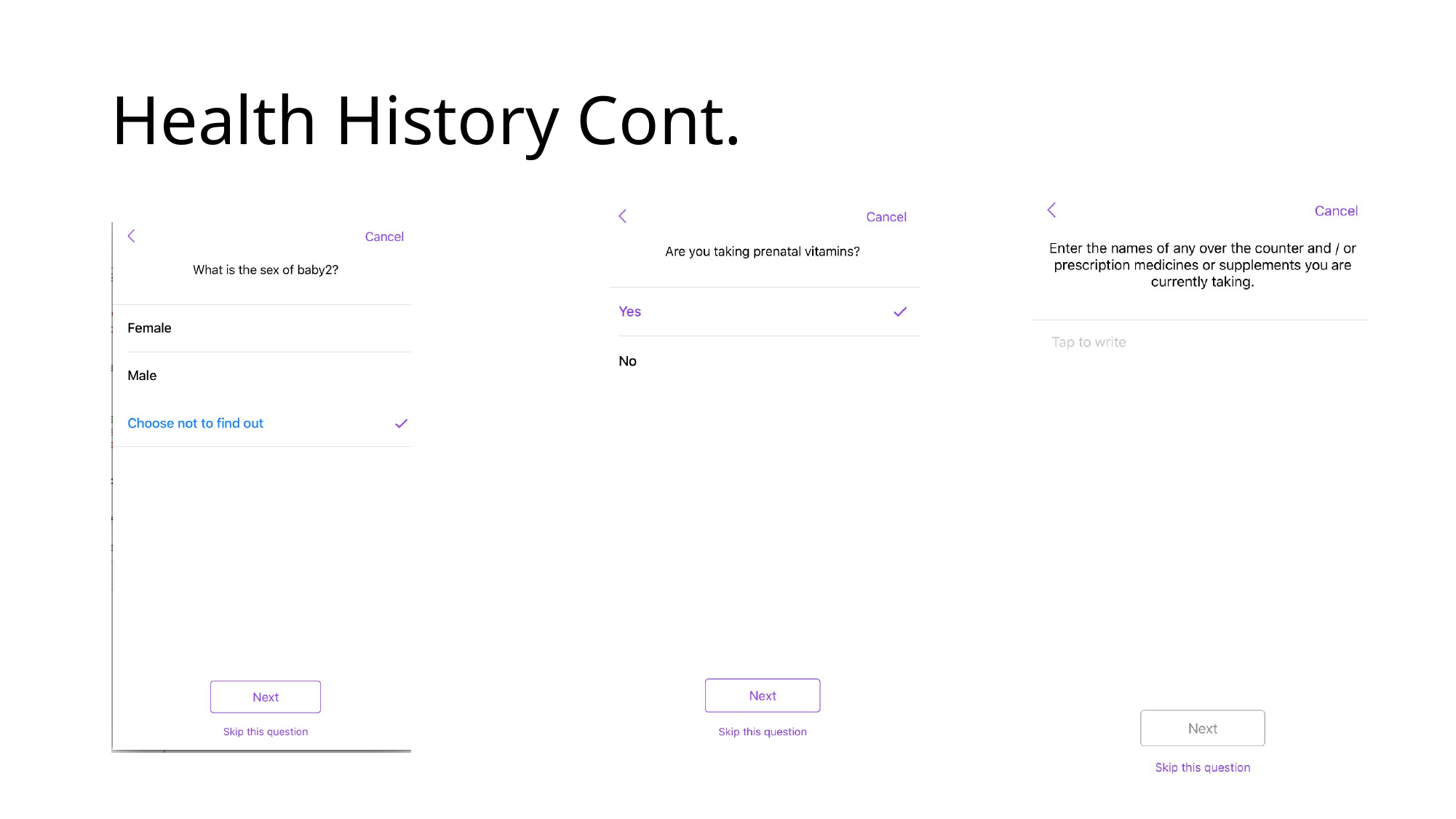

# Health History Cont.

## Slide 14
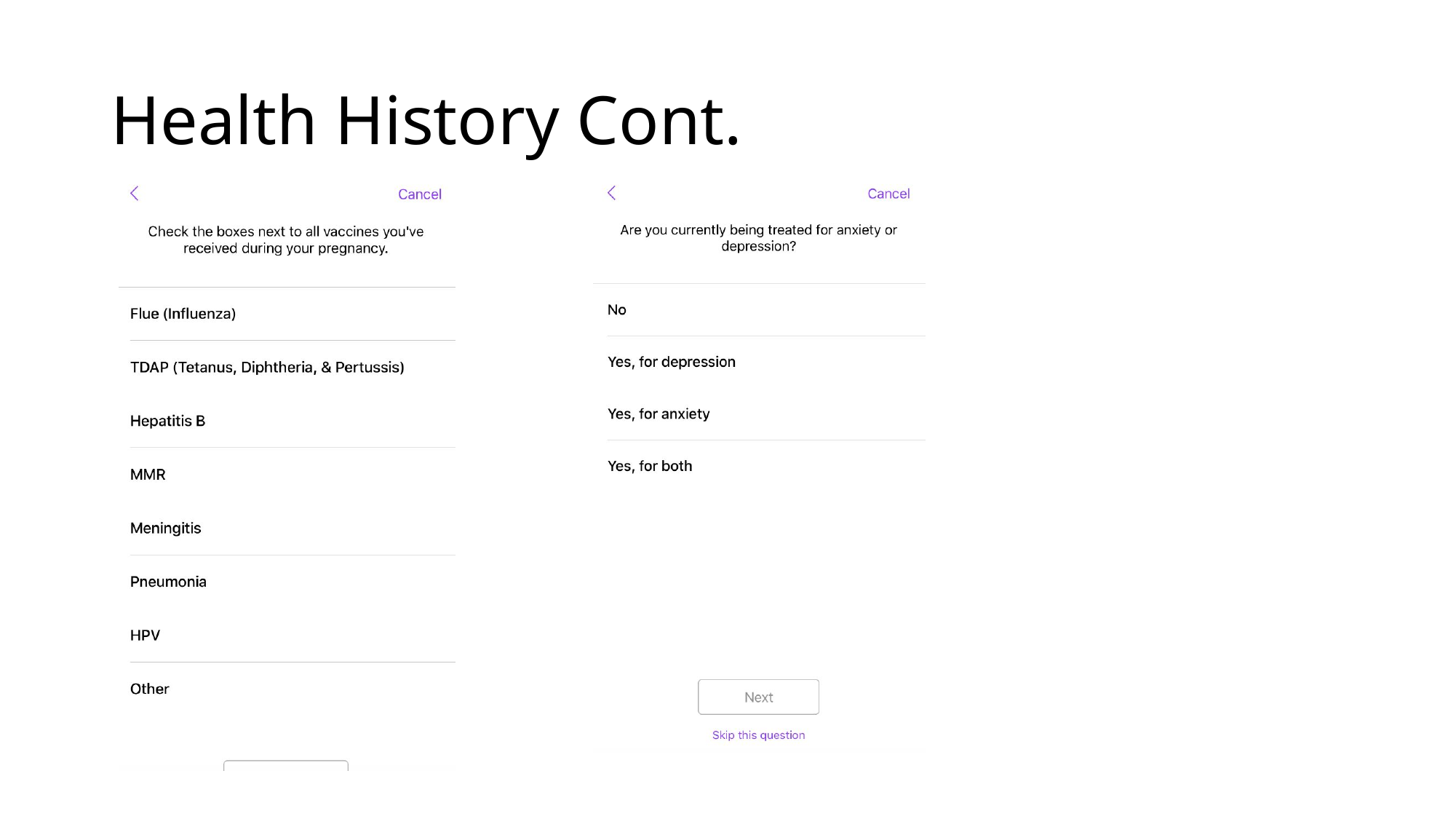

# Health History Cont.

## Slide 15
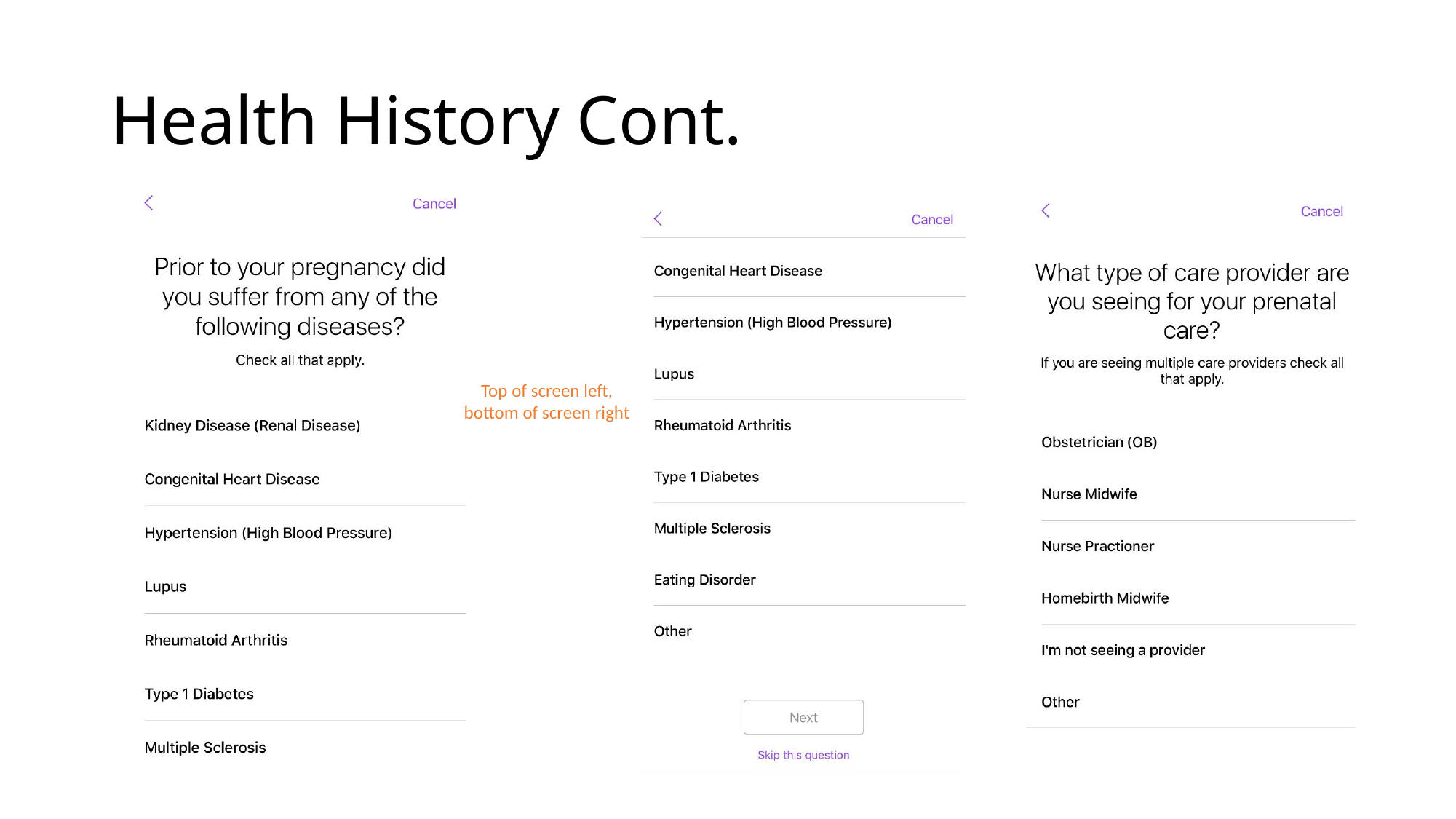

# Health History Cont.
Top of screen left, bottom of screen right

## Slide 16
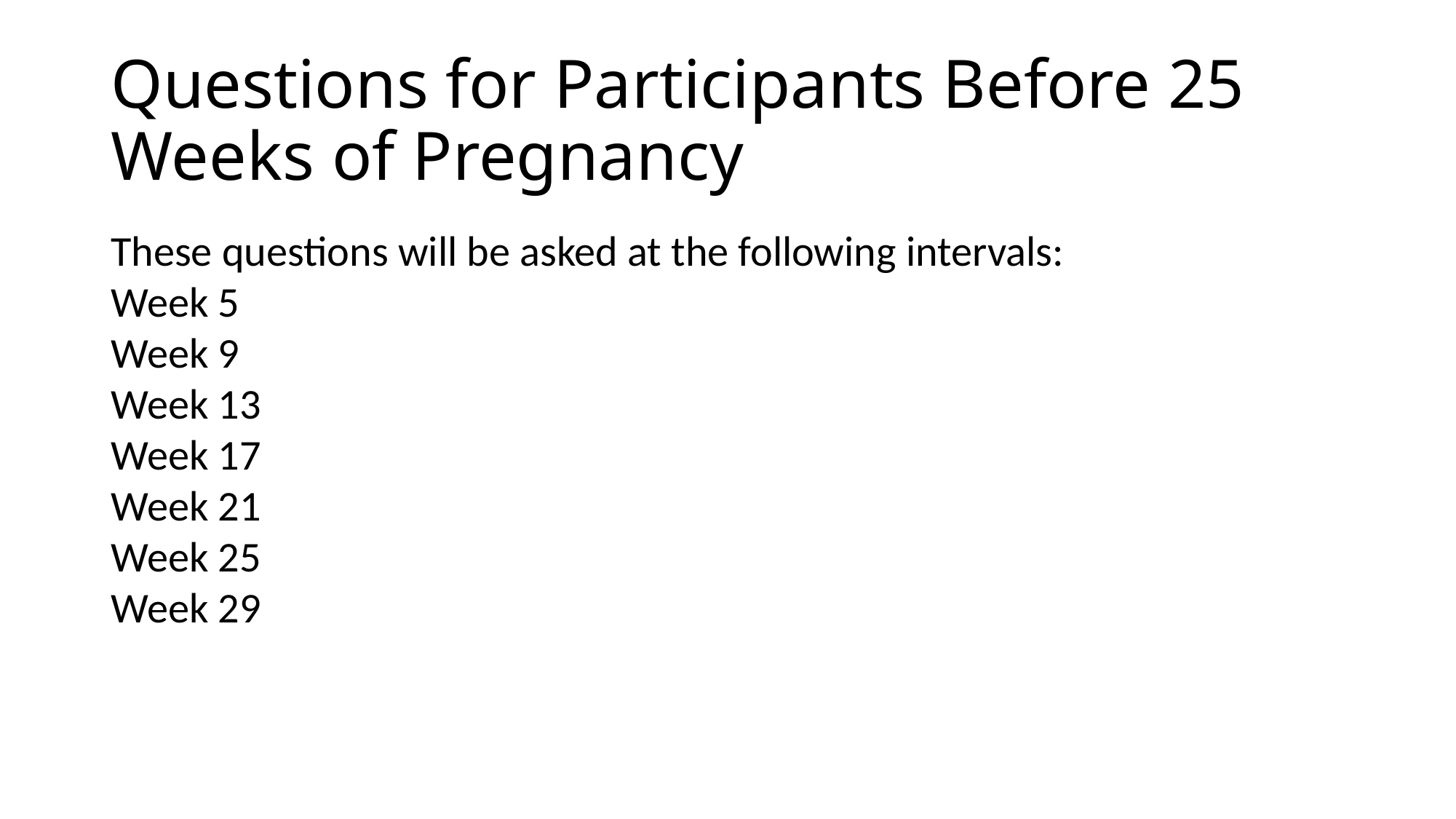

# Questions for Participants Before 25 Weeks of Pregnancy
These questions will be asked at the following intervals:
Week 5
Week 9
Week 13
Week 17
Week 21
Week 25
Week 29

## Slide 17
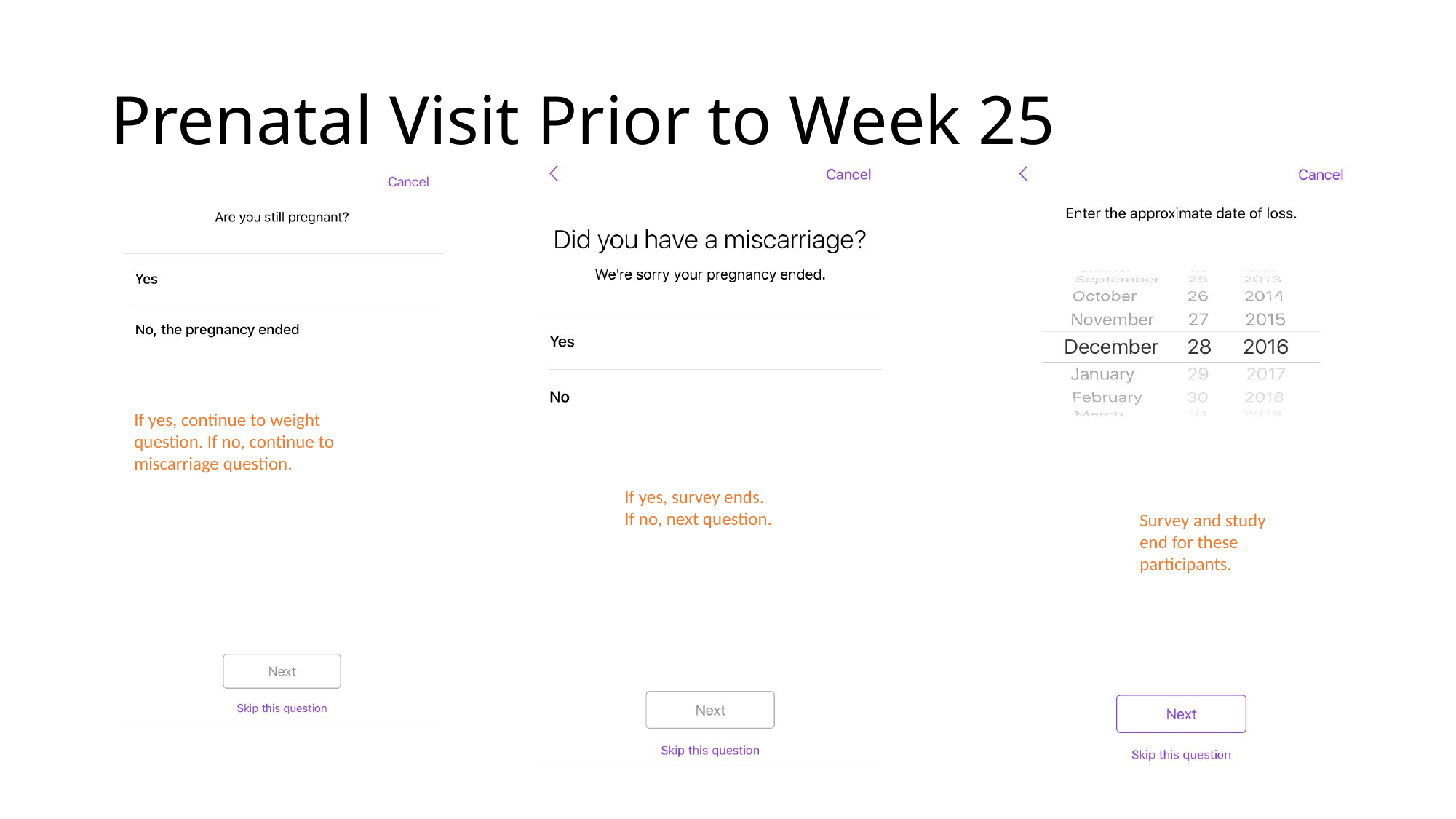

# Prenatal Visit Prior to Week 25
If yes, continue to weight question. If no, continue to miscarriage question.
If yes, survey ends.
If no, next question.
Survey and study end for these participants.

## Slide 18
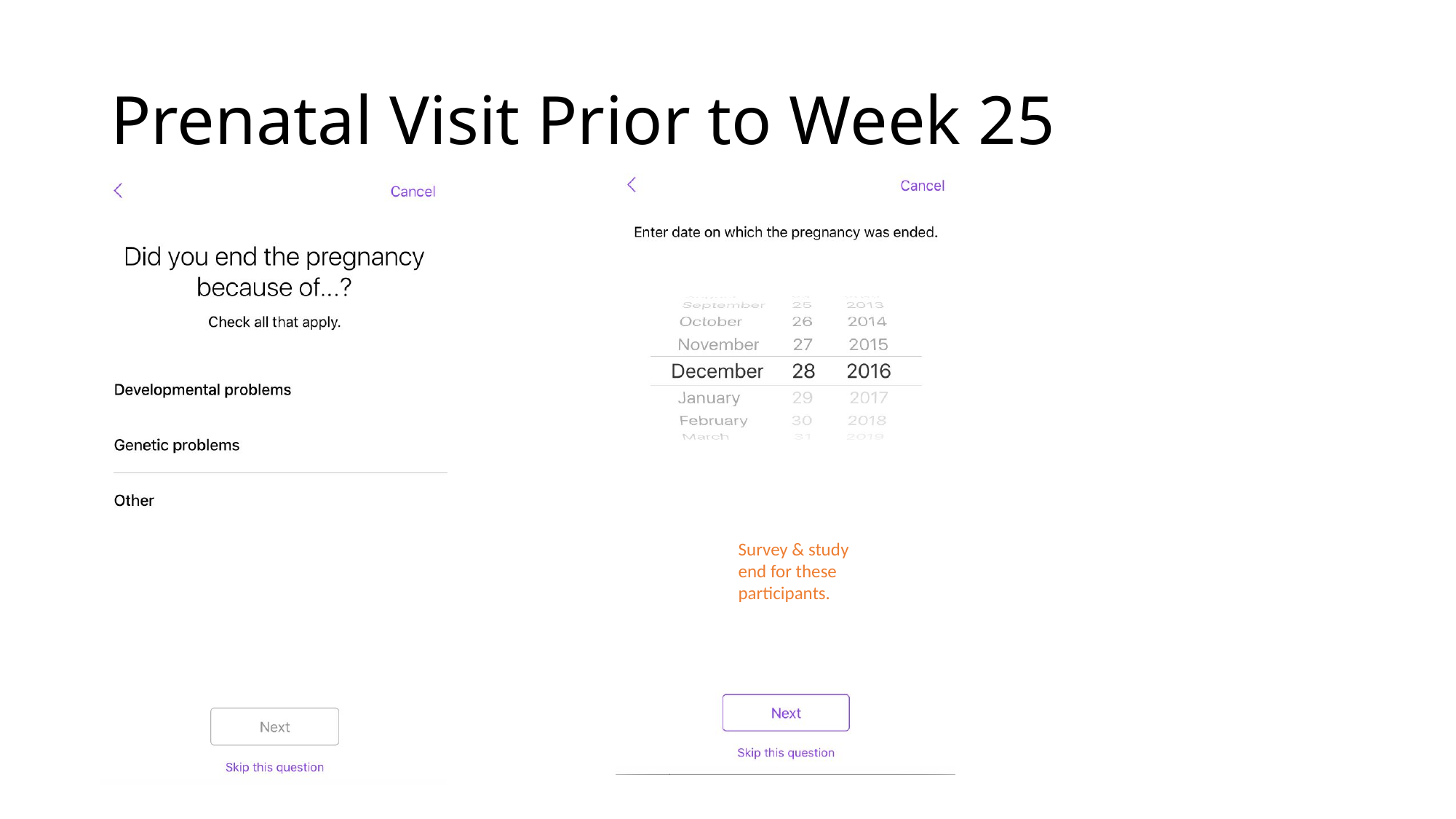

# Prenatal Visit Prior to Week 25
Survey & study end for these participants.

## Slide 19
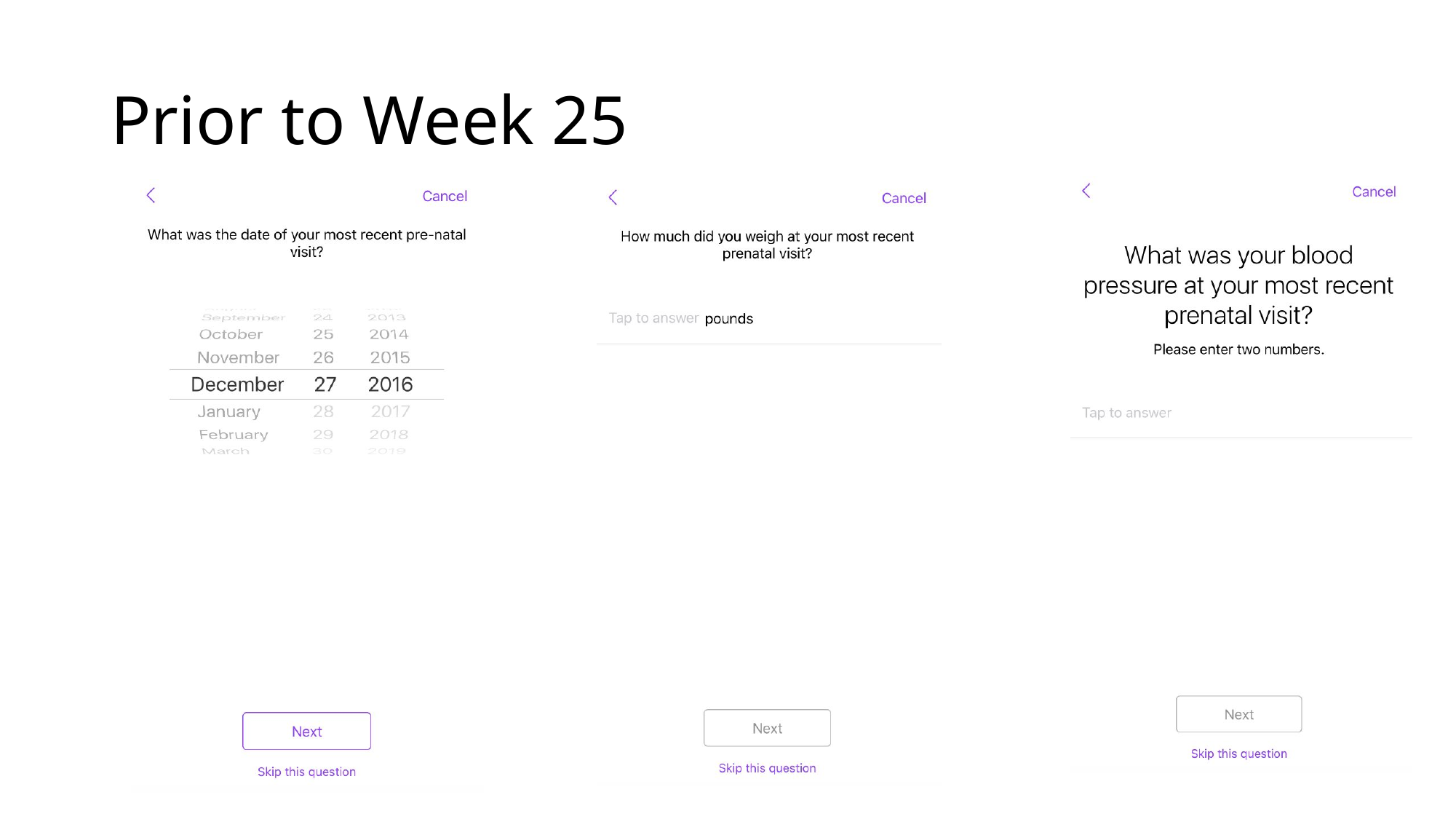

# Prior to Week 25

## Slide 20
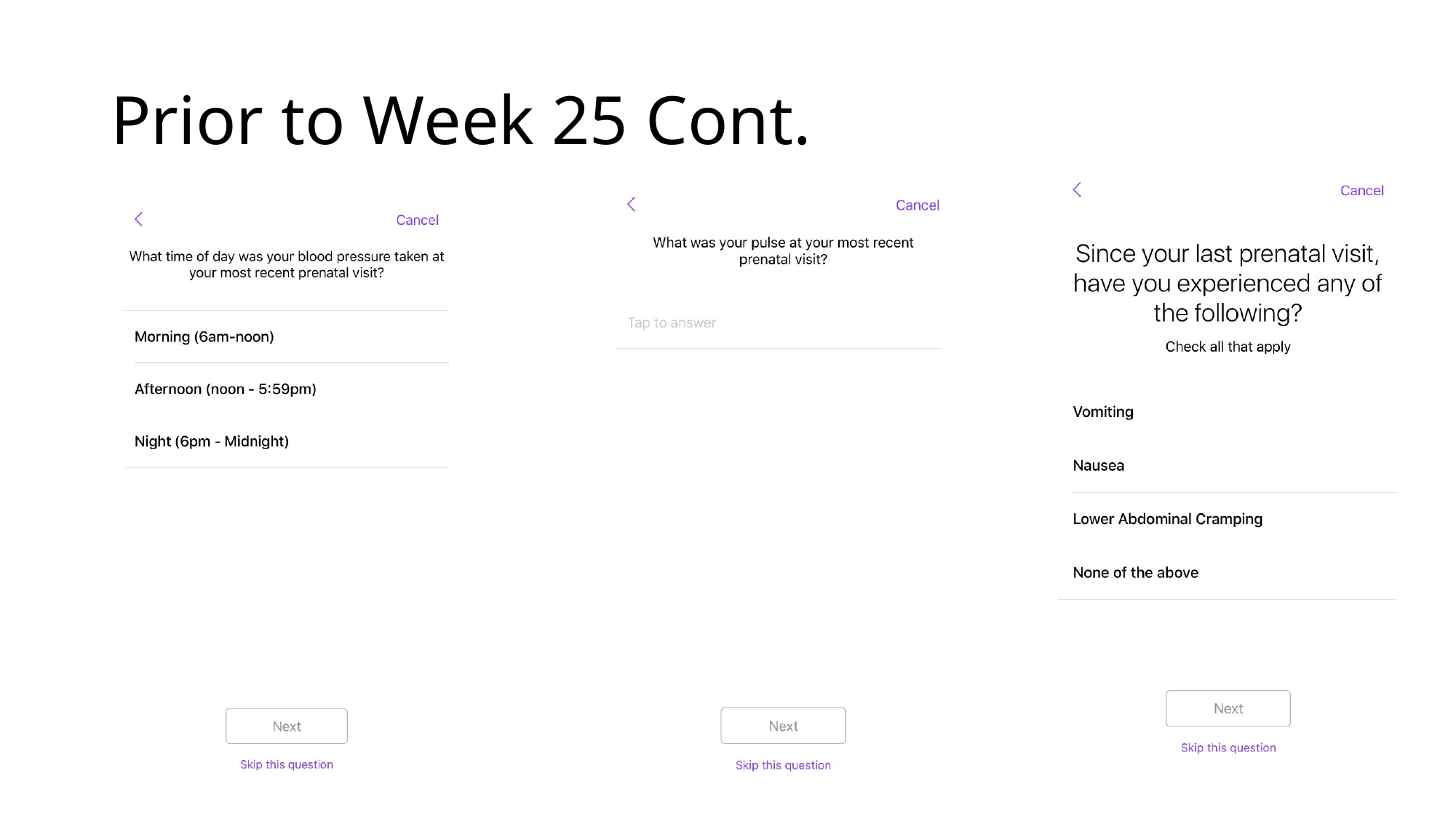

# Prior to Week 25 Cont.

## Slide 21
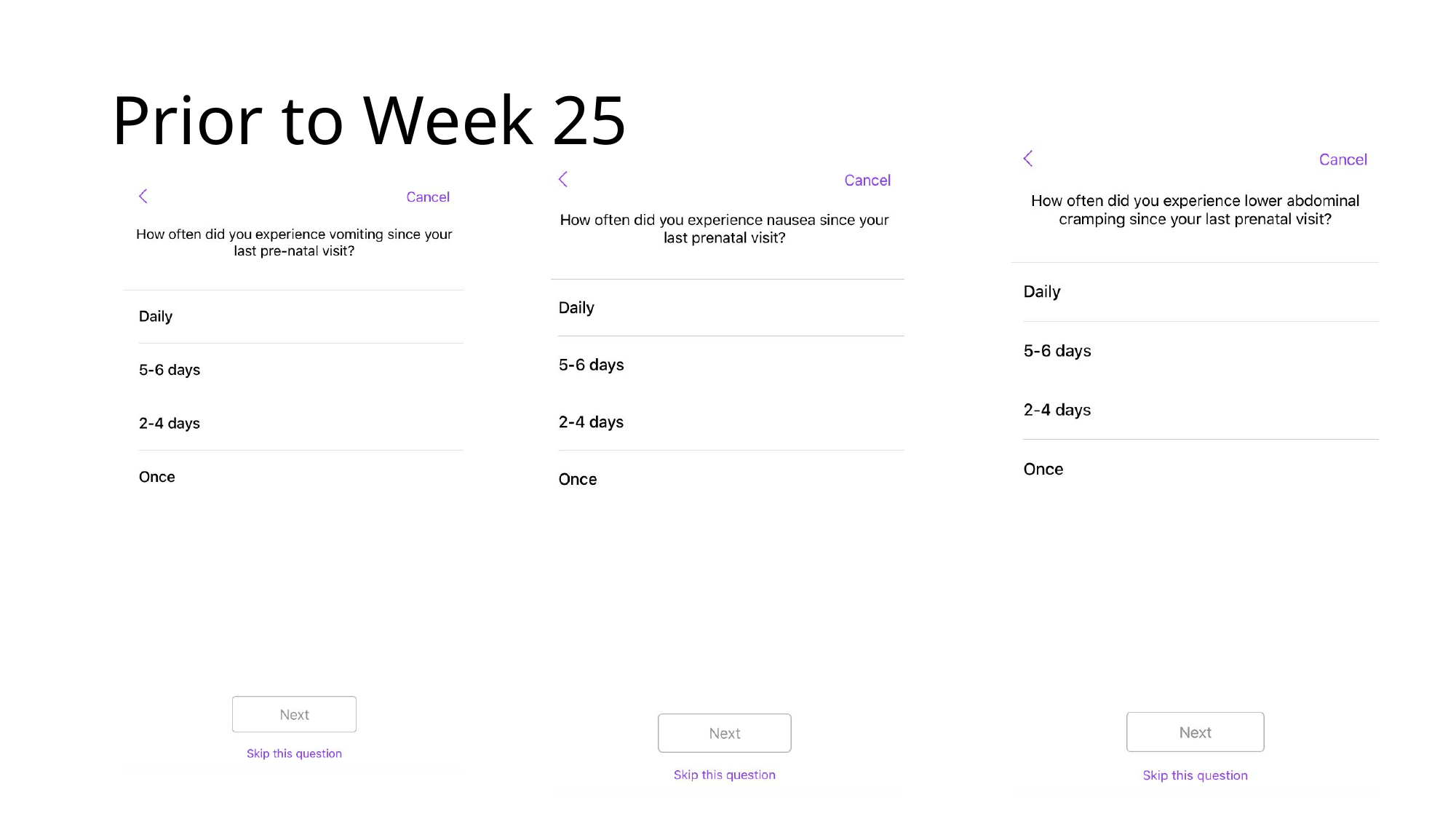

# Prior to Week 25

## Slide 22
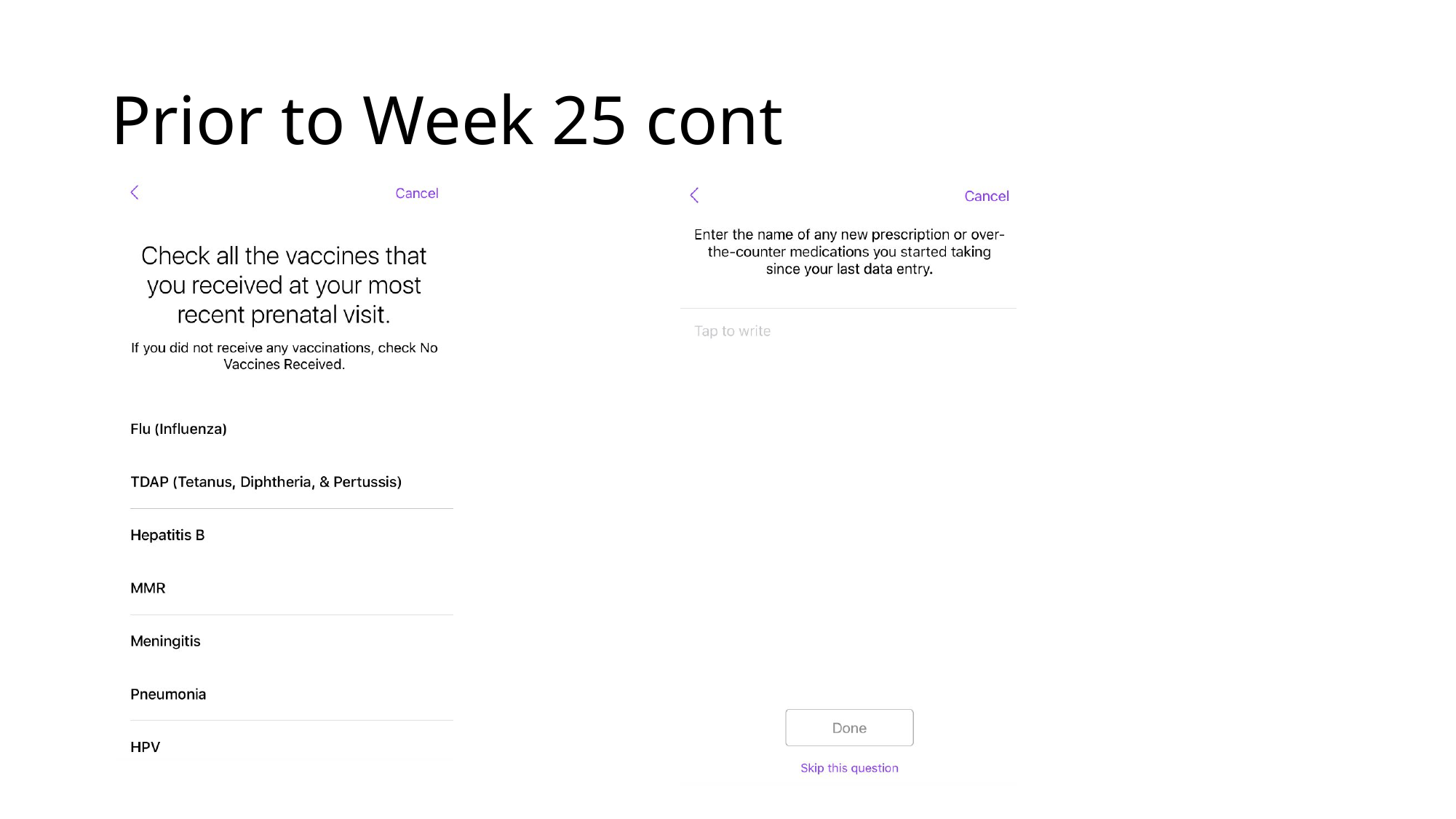

# Prior to Week 25 cont

## Slide 23
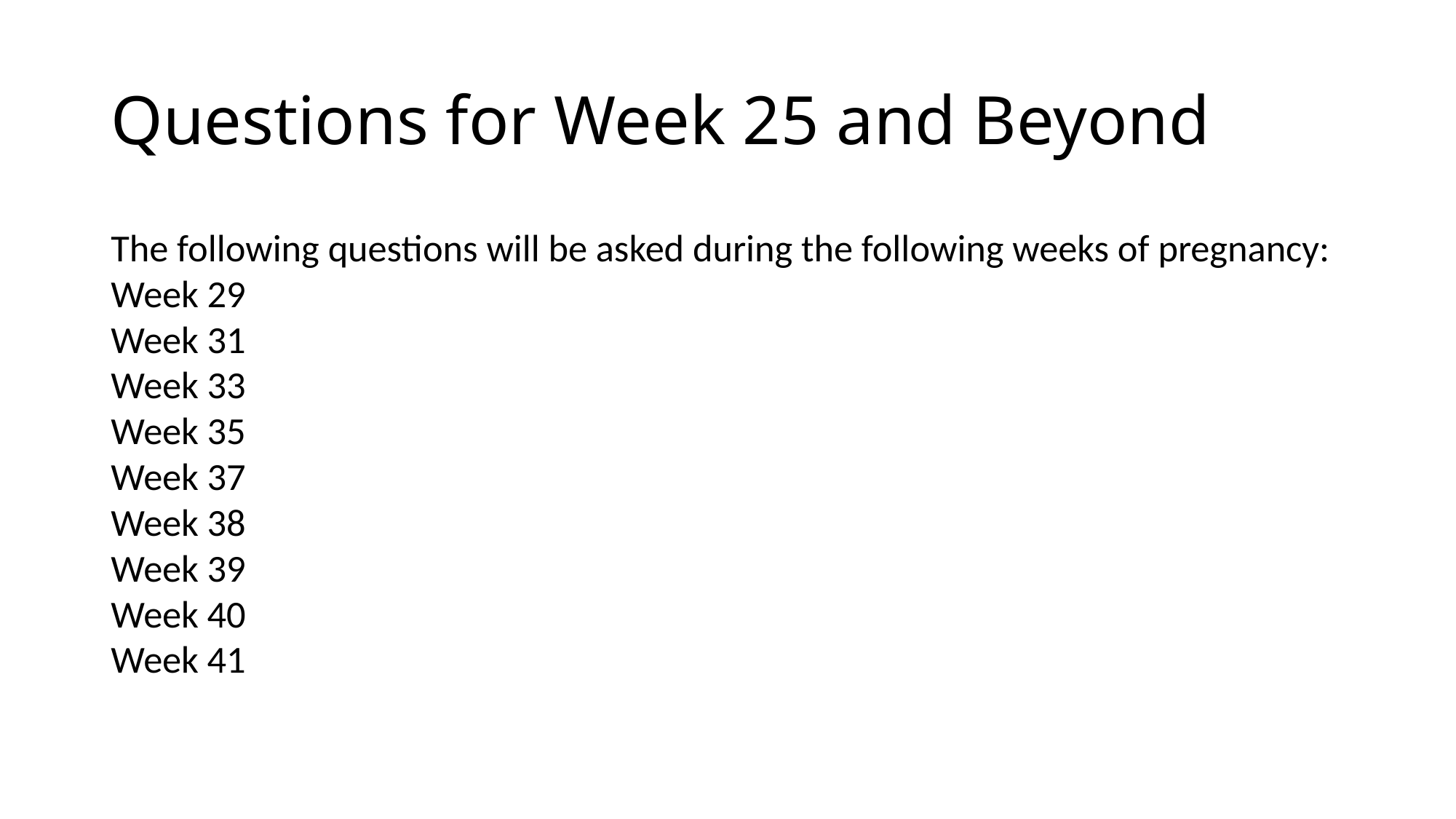

# Questions for Week 25 and Beyond
The following questions will be asked during the following weeks of pregnancy:
Week 29
Week 31
Week 33
Week 35
Week 37
Week 38
Week 39
Week 40
Week 41

## Slide 24
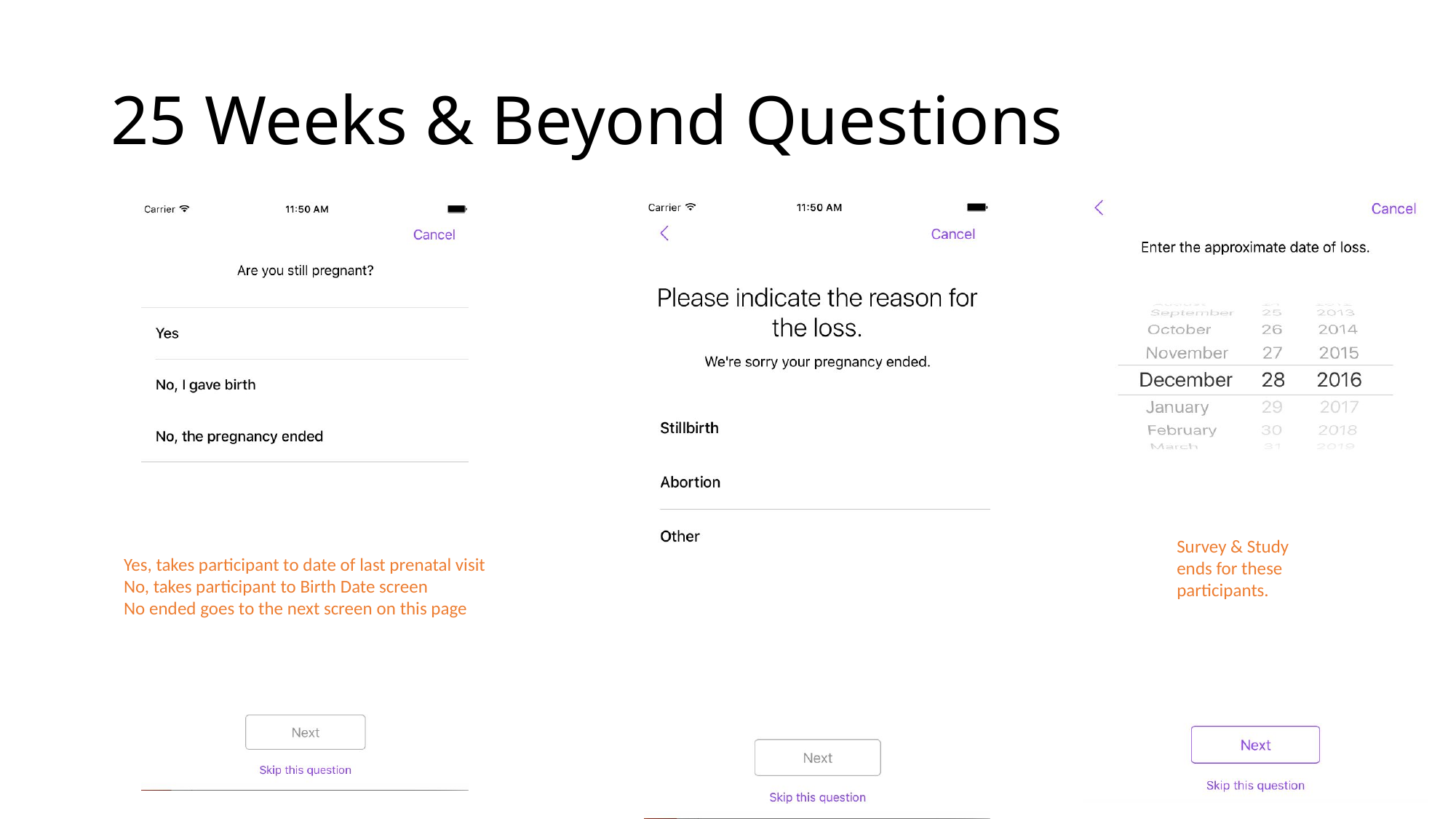

# 25 Weeks & Beyond Questions
Survey & Study ends for these participants.
Yes, takes participant to date of last prenatal visit
No, takes participant to Birth Date screen
No ended goes to the next screen on this page

## Slide 25
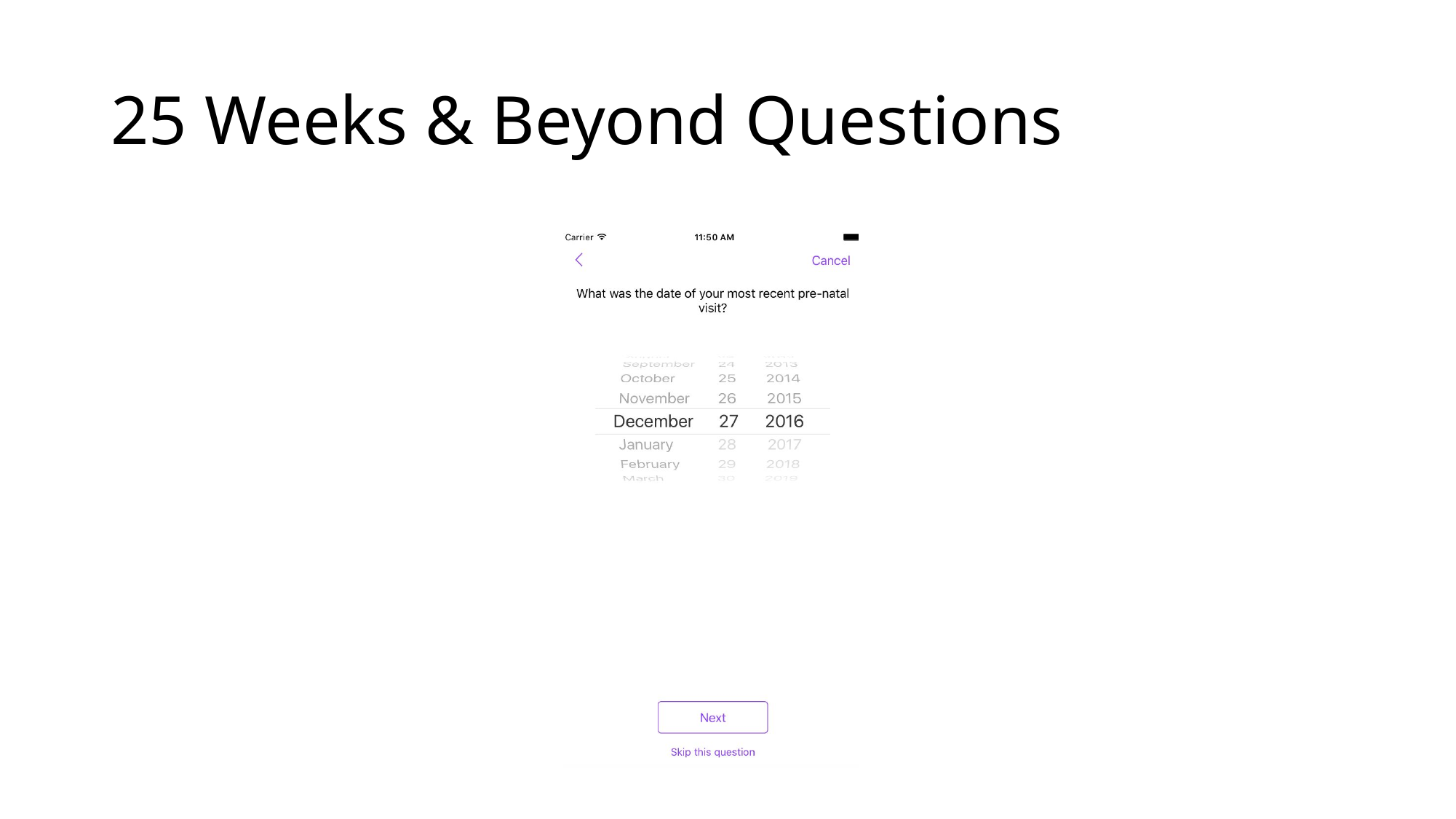

# 25 Weeks & Beyond Questions

## Slide 26
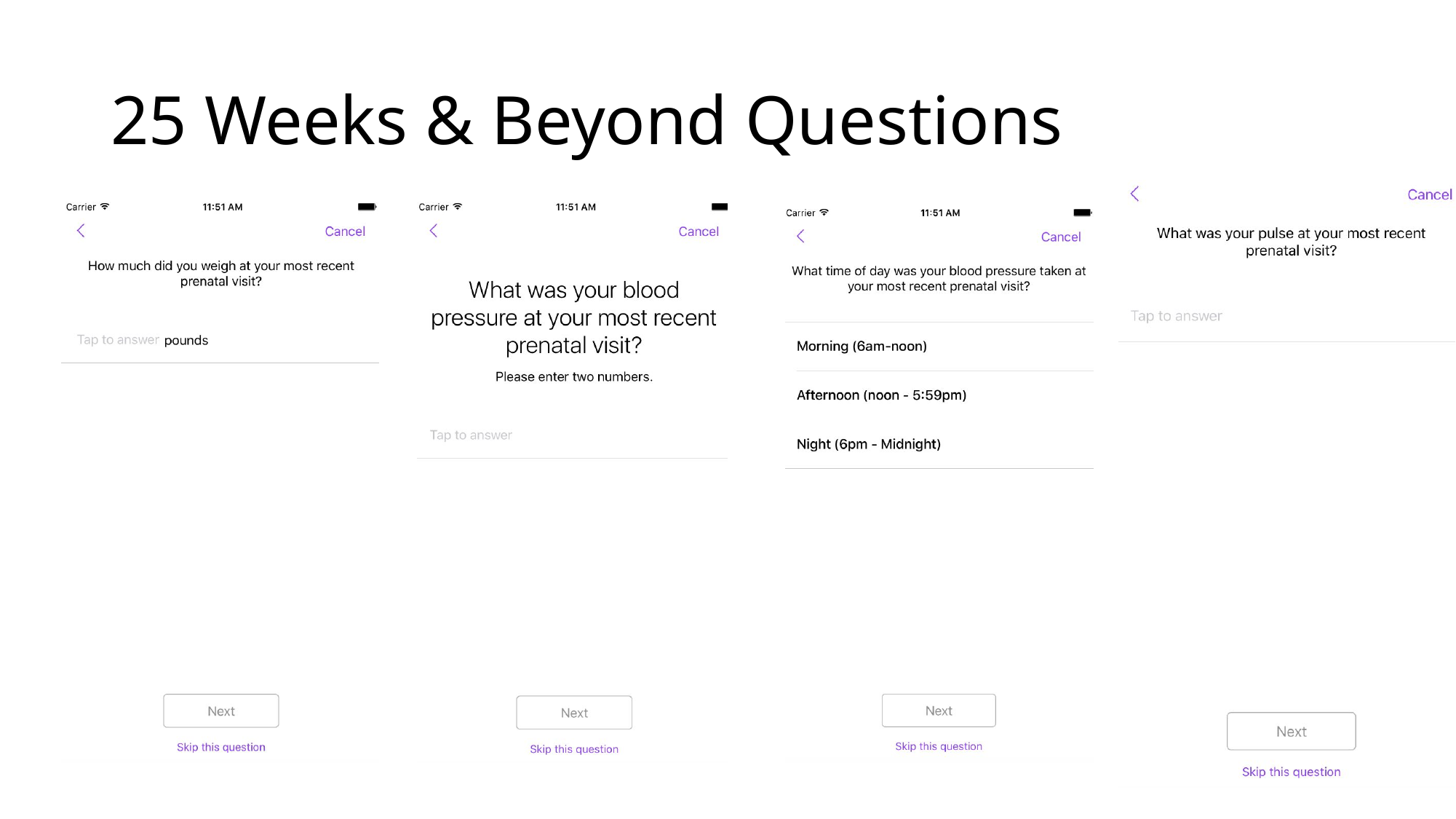

# 25 Weeks & Beyond Questions

## Slide 27
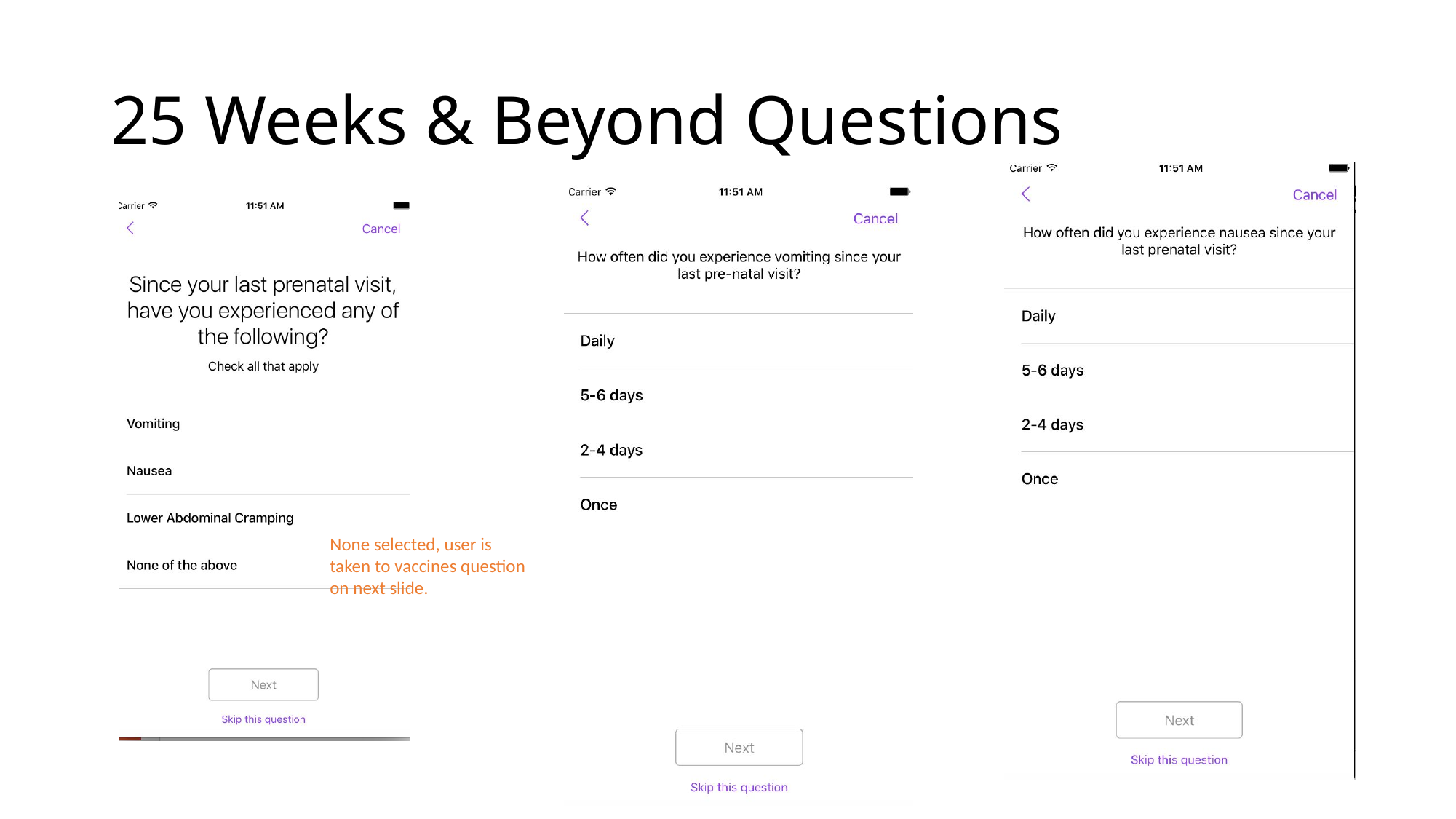

# 25 Weeks & Beyond Questions
None selected, user is taken to vaccines question on next slide.

## Slide 28
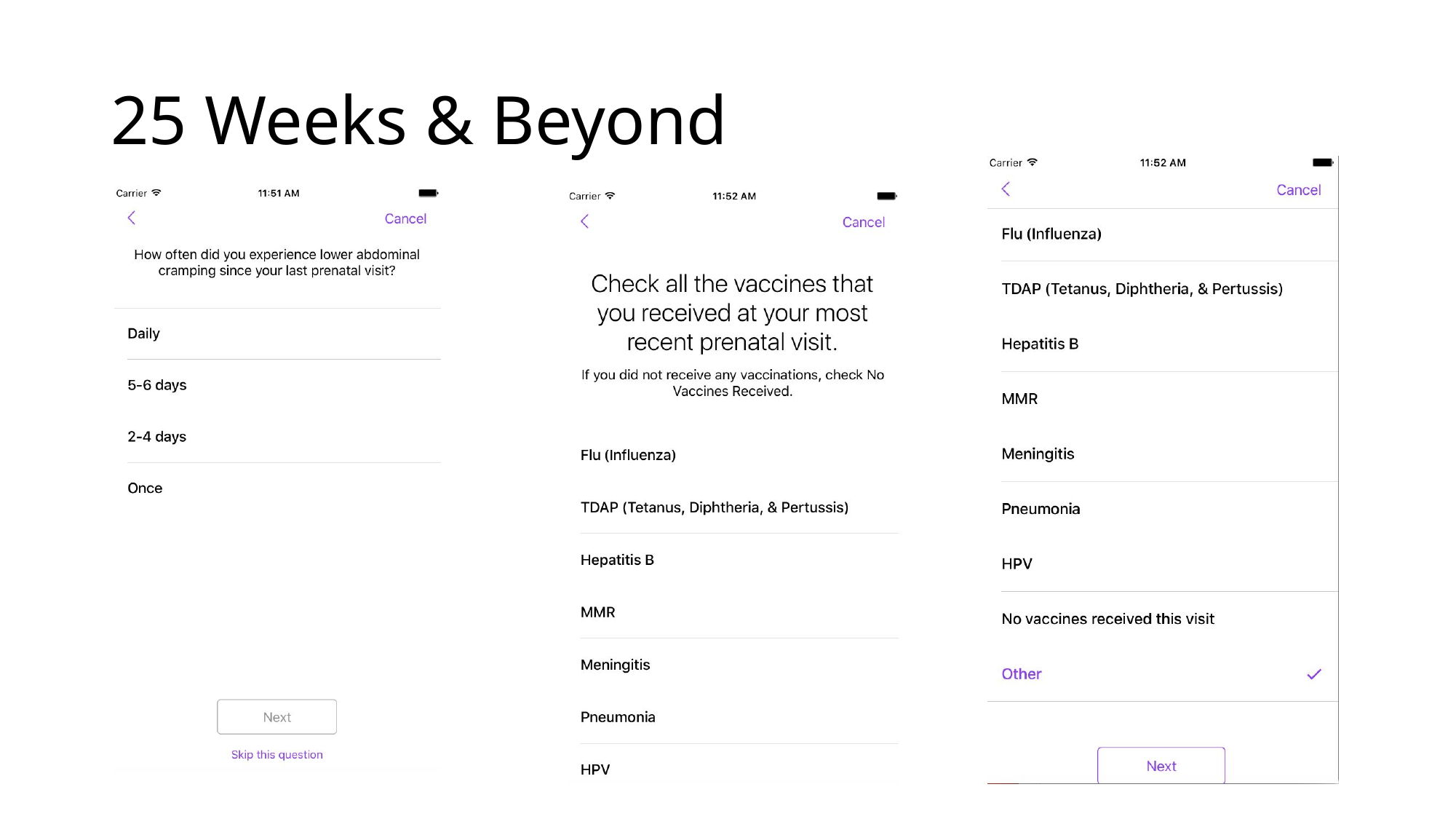

# 25 Weeks & Beyond

## Slide 29
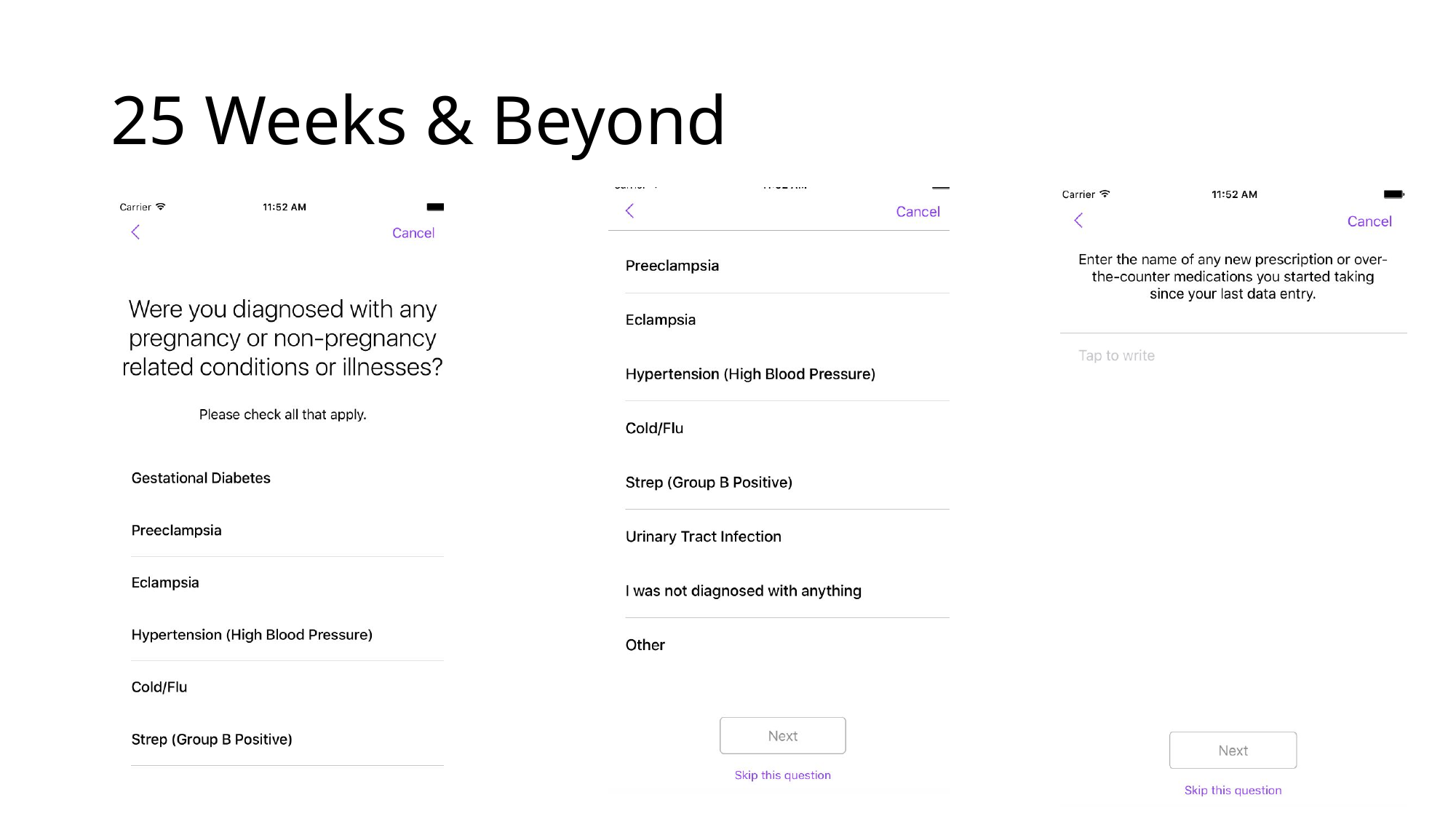

# 25 Weeks & Beyond

## Slide 30
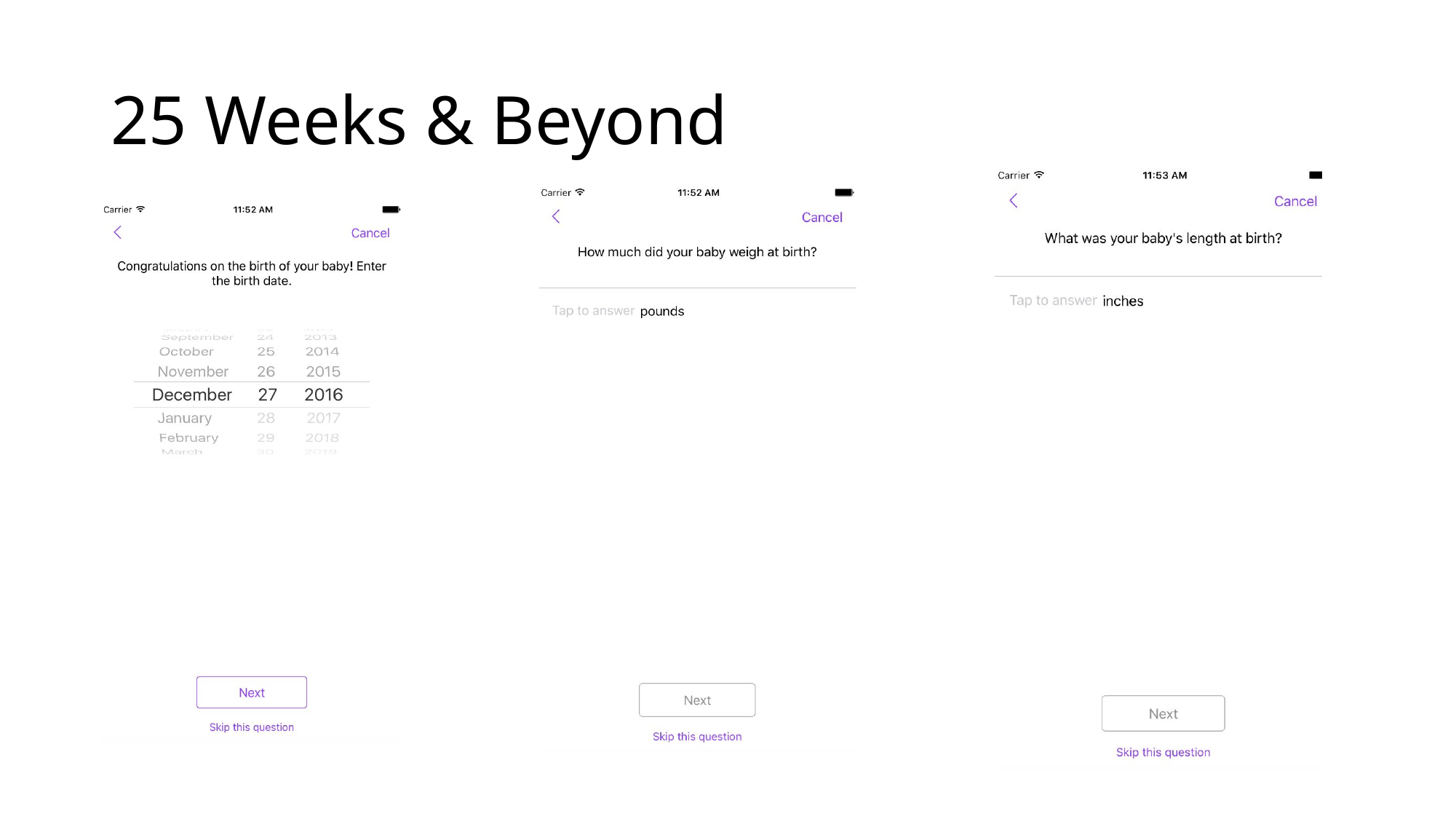

# 25 Weeks & Beyond

## Slide 31
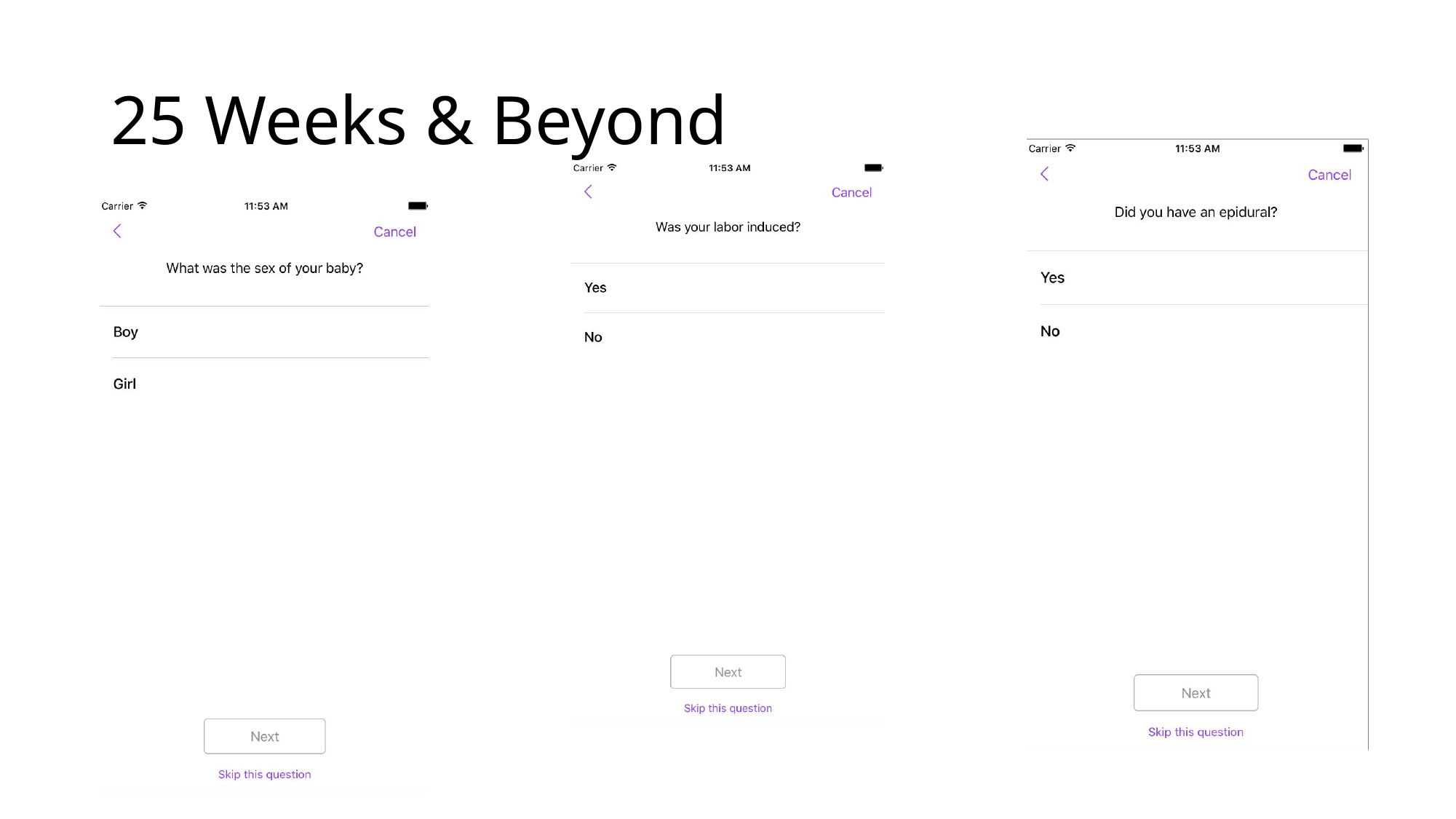

# 25 Weeks & Beyond

## Slide 32
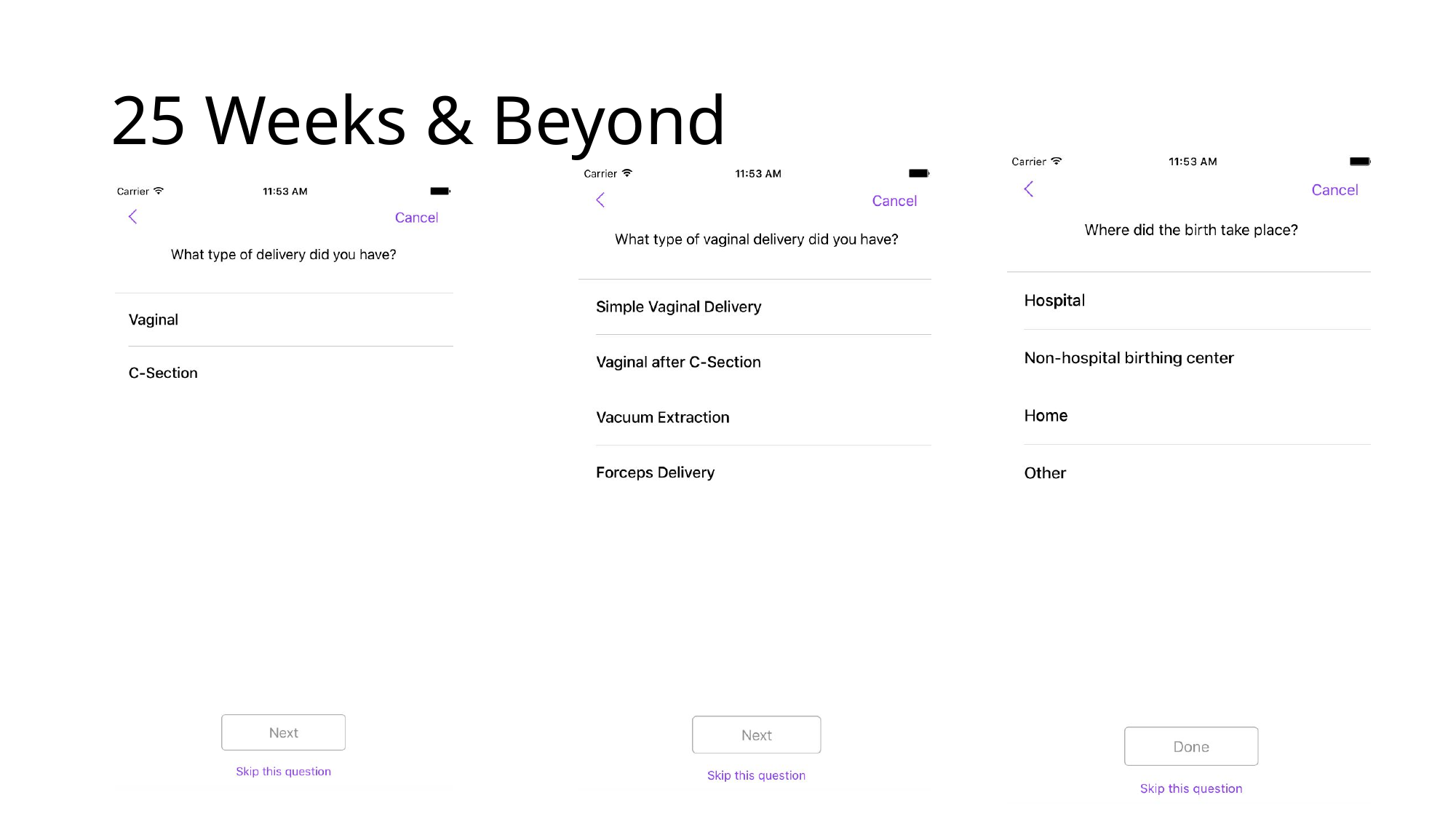

# 25 Weeks & Beyond

## Slide 33
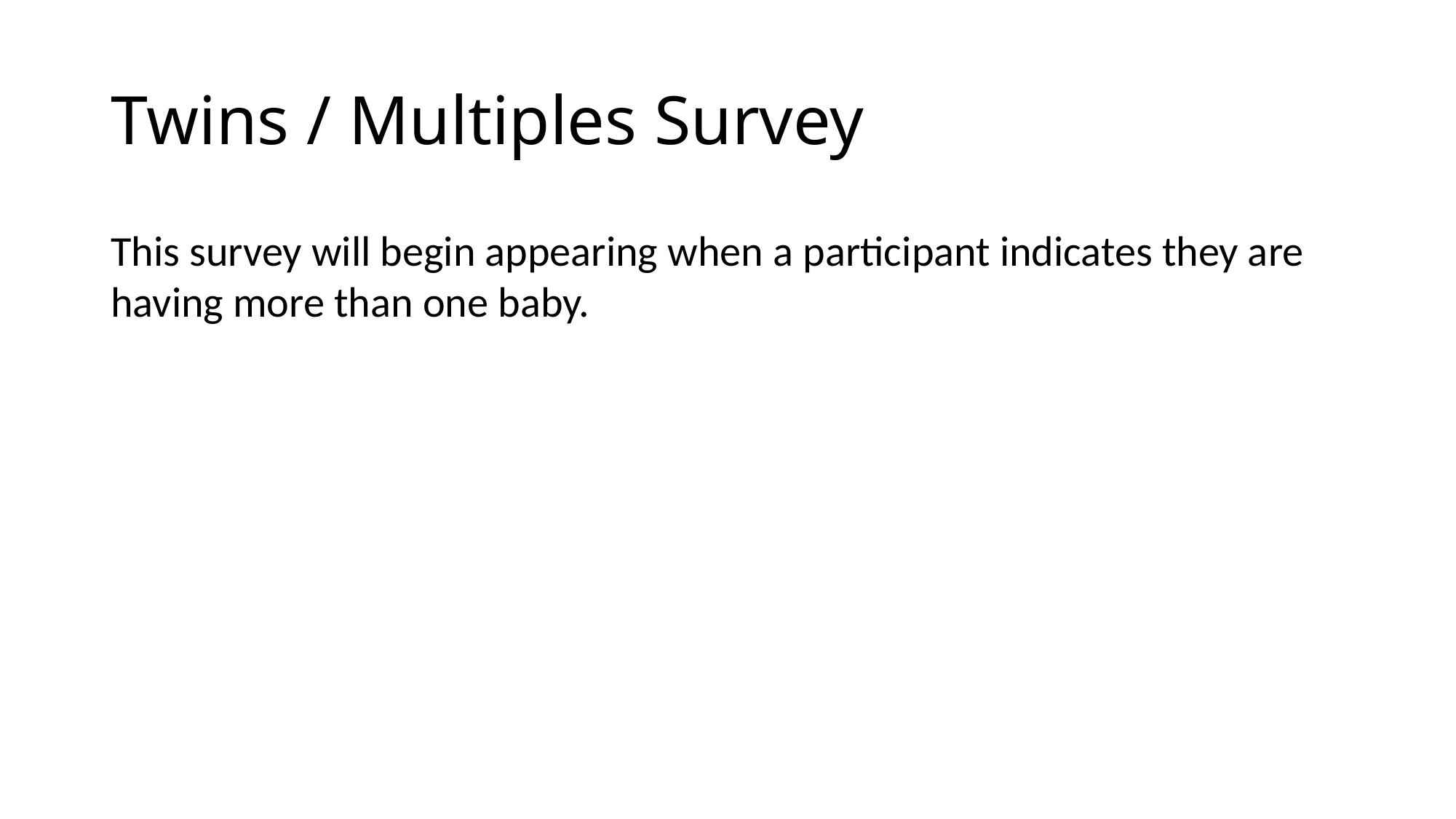

# Twins / Multiples Survey
This survey will begin appearing when a participant indicates they are having more than one baby.

## Slide 34
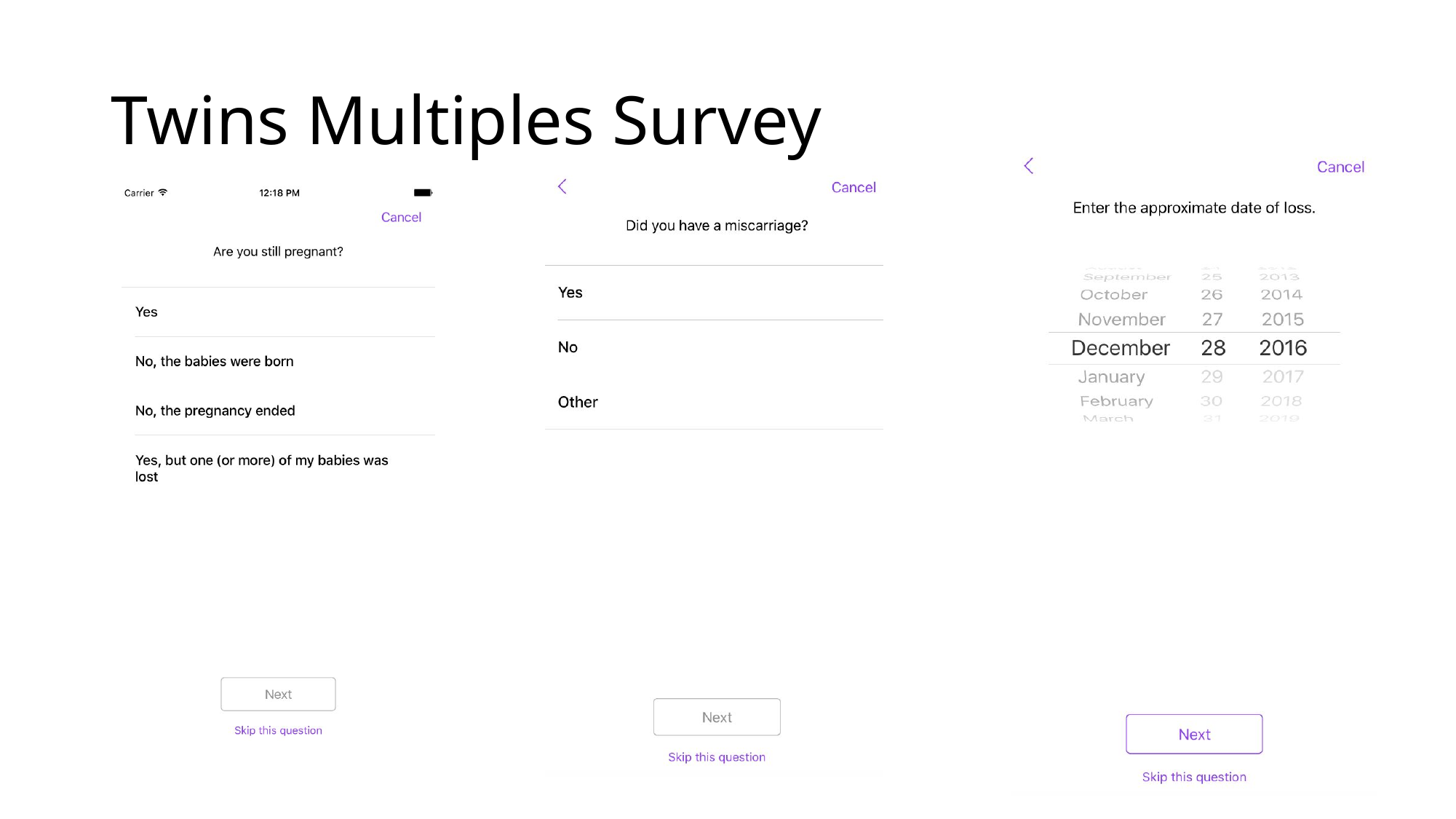

# Twins Multiples Survey

## Slide 35
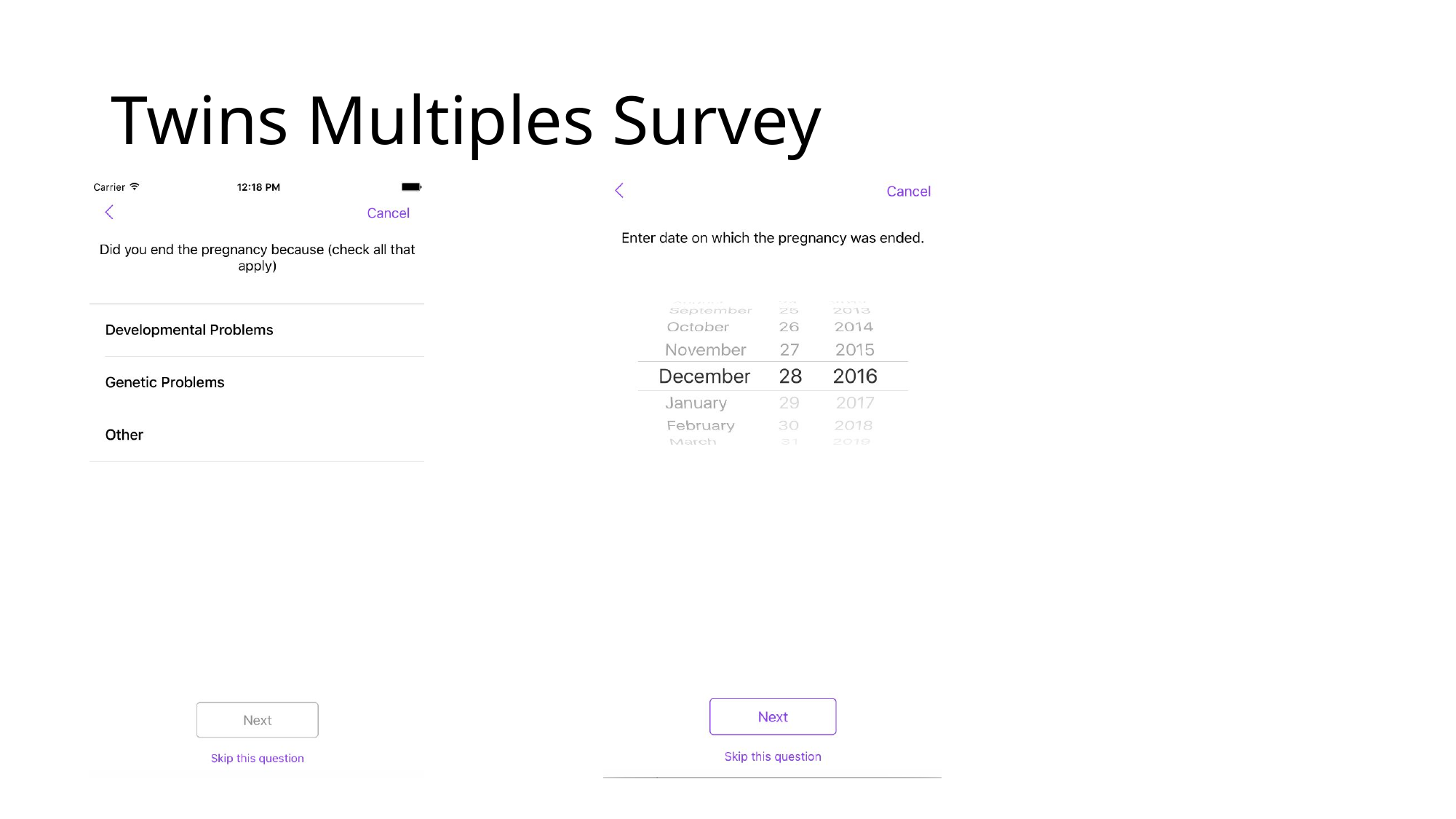

# Twins Multiples Survey

## Slide 36
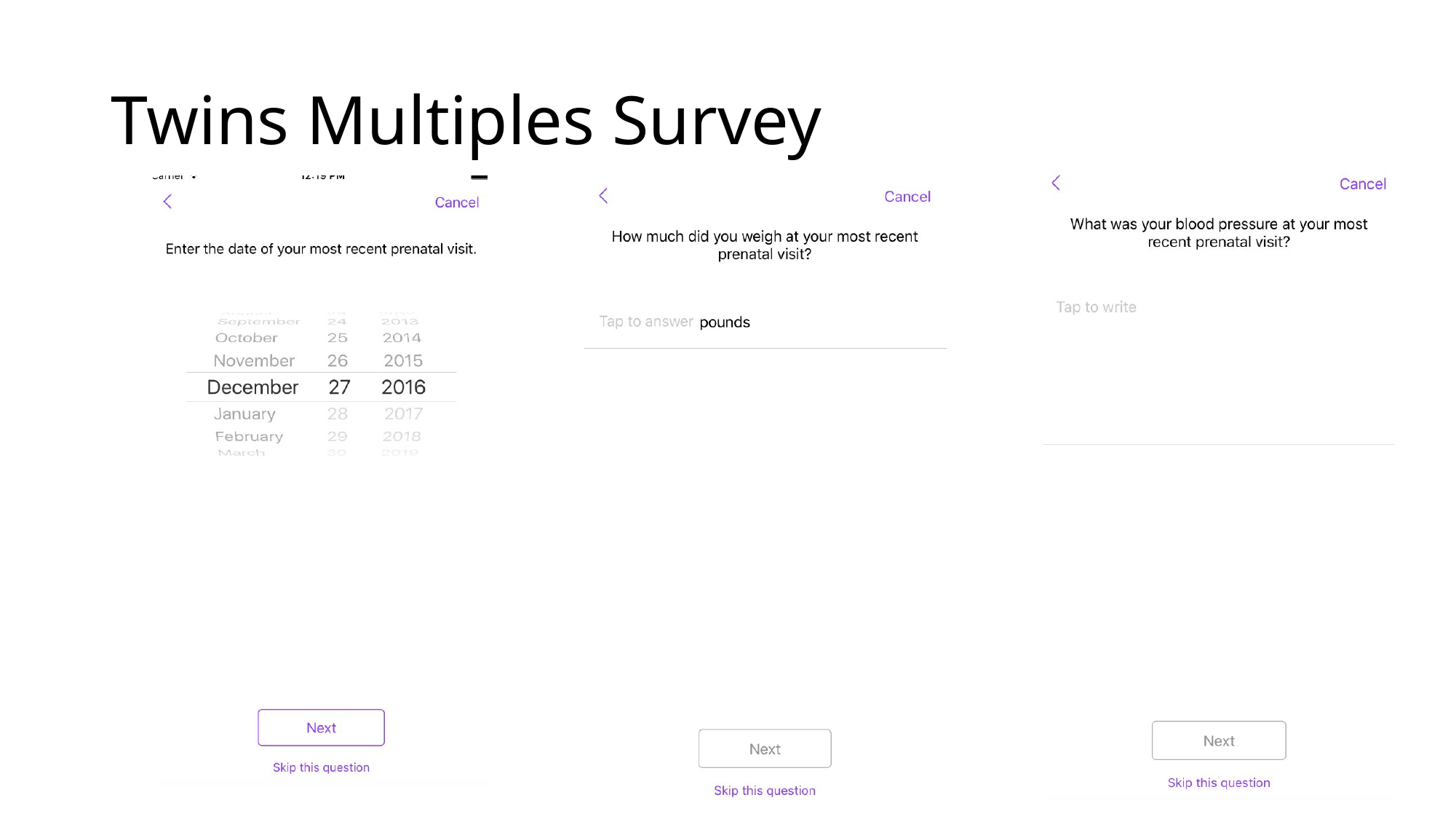

# Twins Multiples Survey

## Slide 37
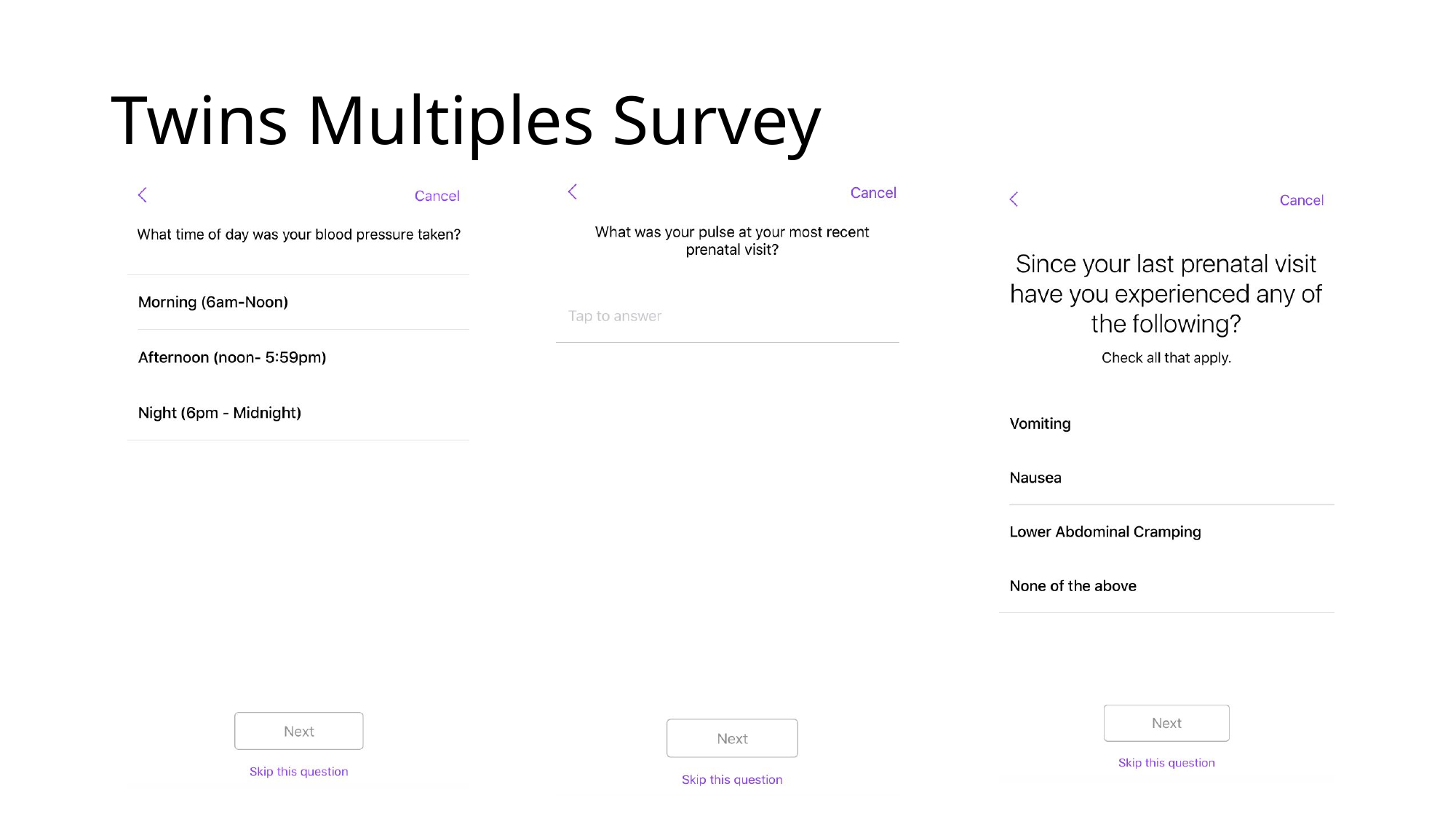

# Twins Multiples Survey

## Slide 38
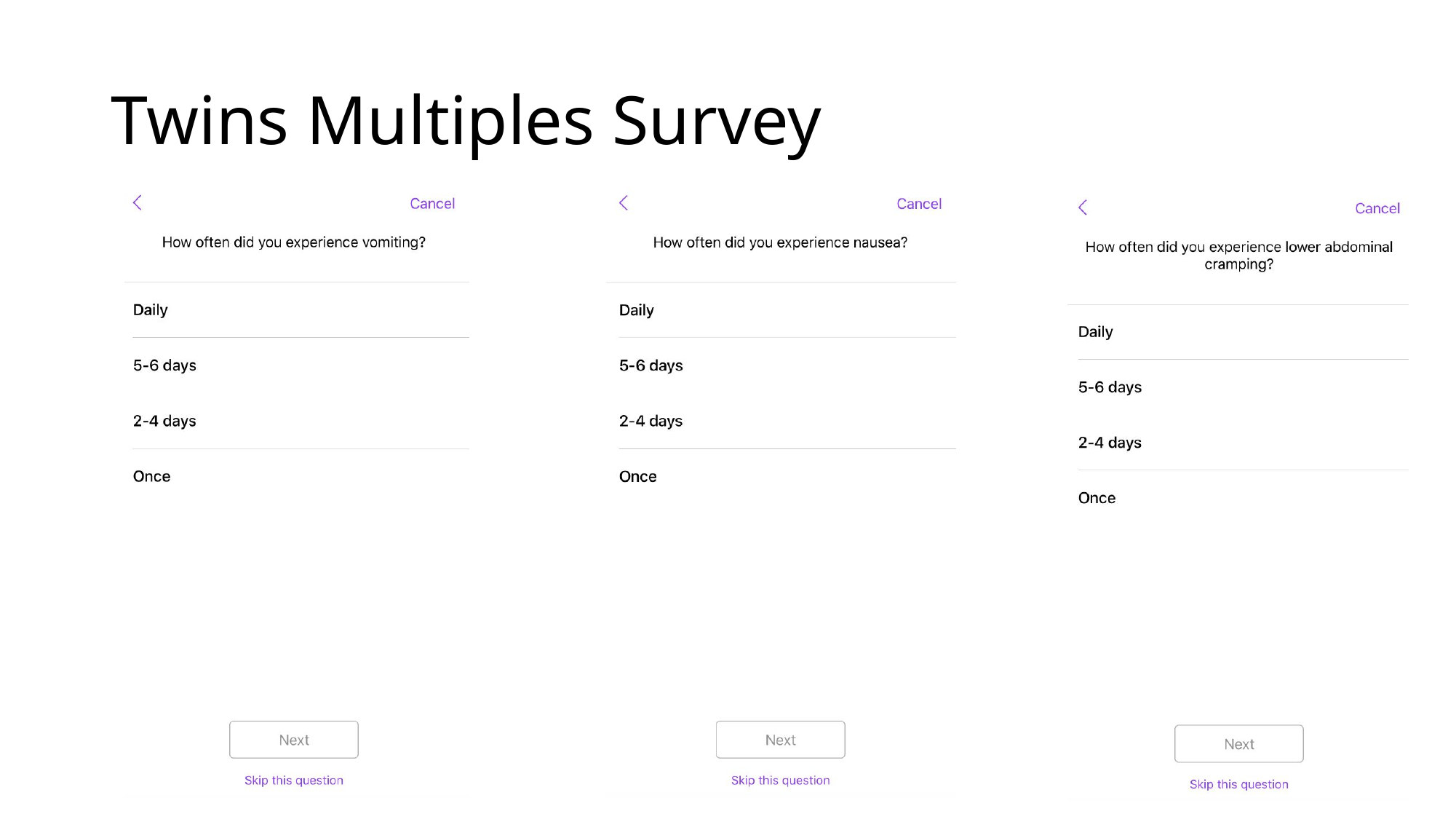

# Twins Multiples Survey

## Slide 39
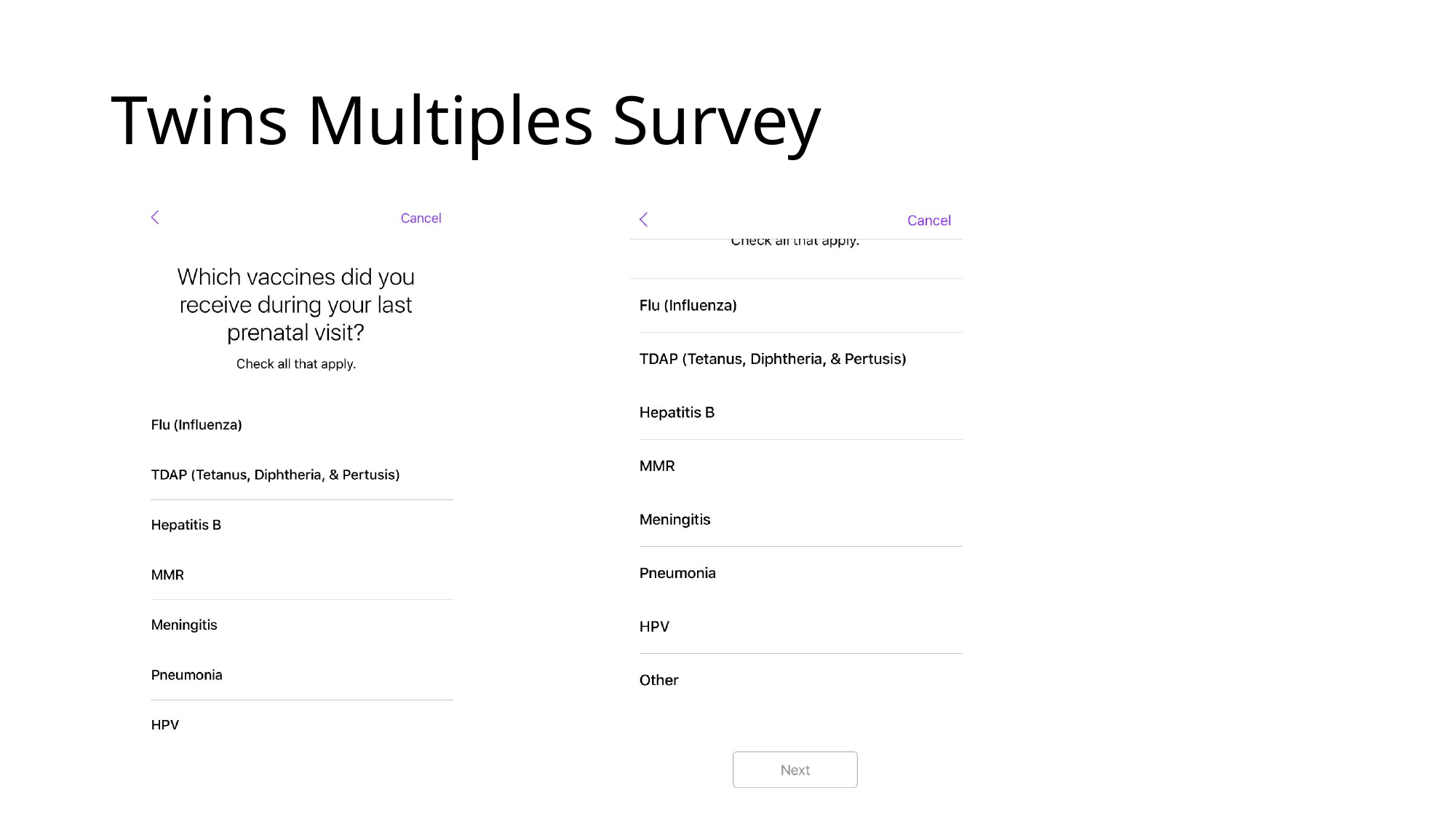

# Twins Multiples Survey

## Slide 40
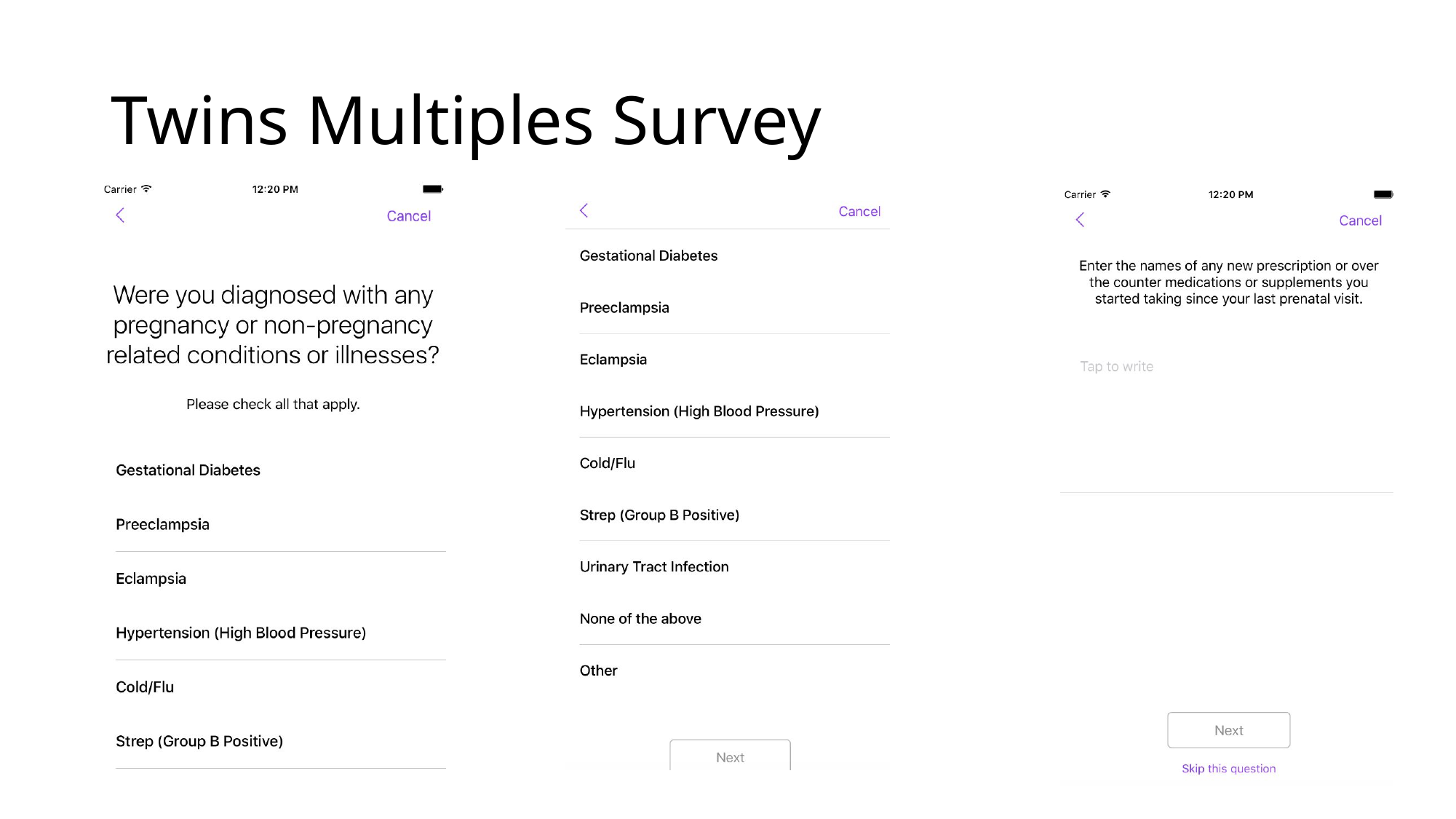

# Twins Multiples Survey

## Slide 41
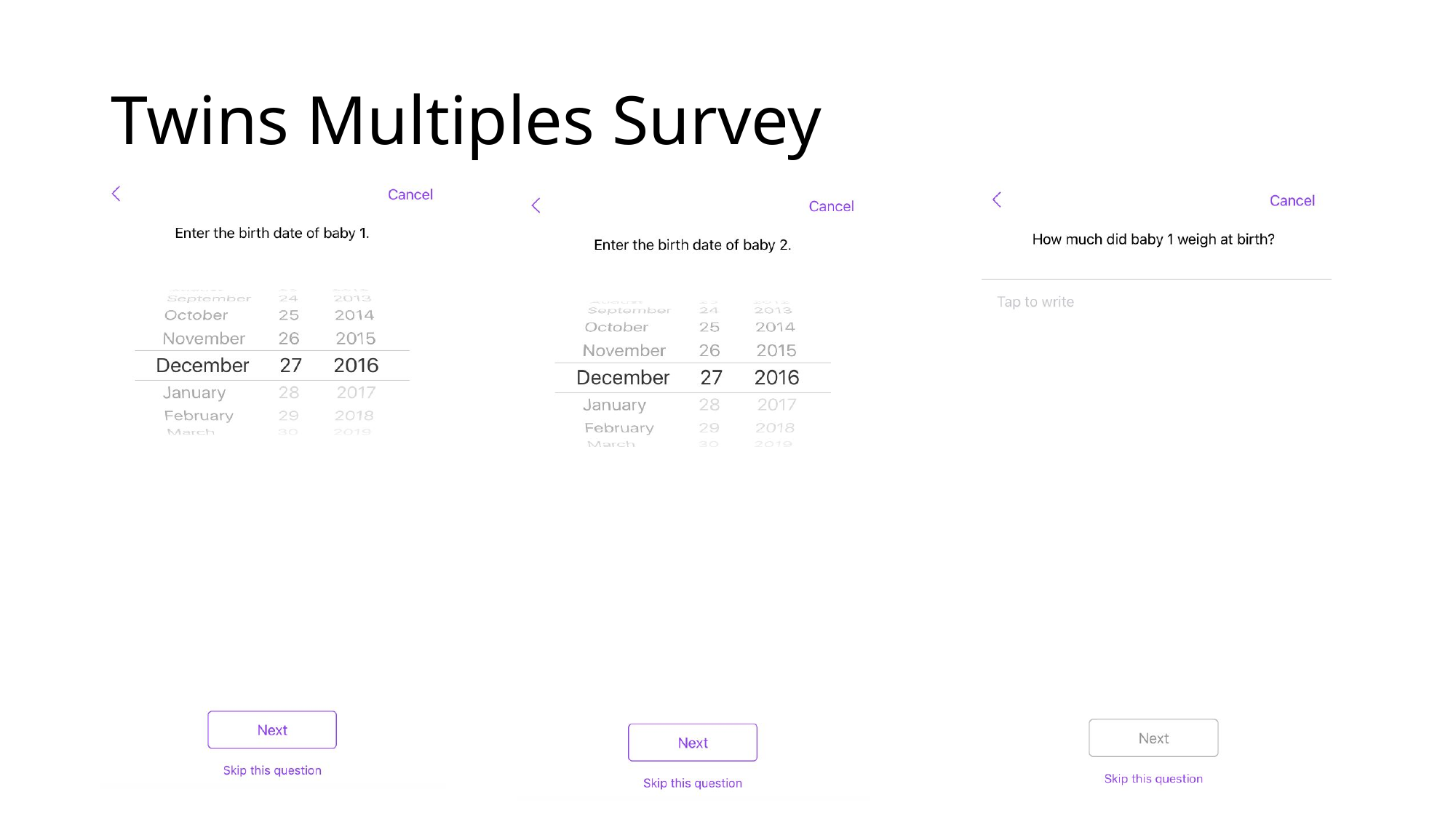

# Twins Multiples Survey

## Slide 42
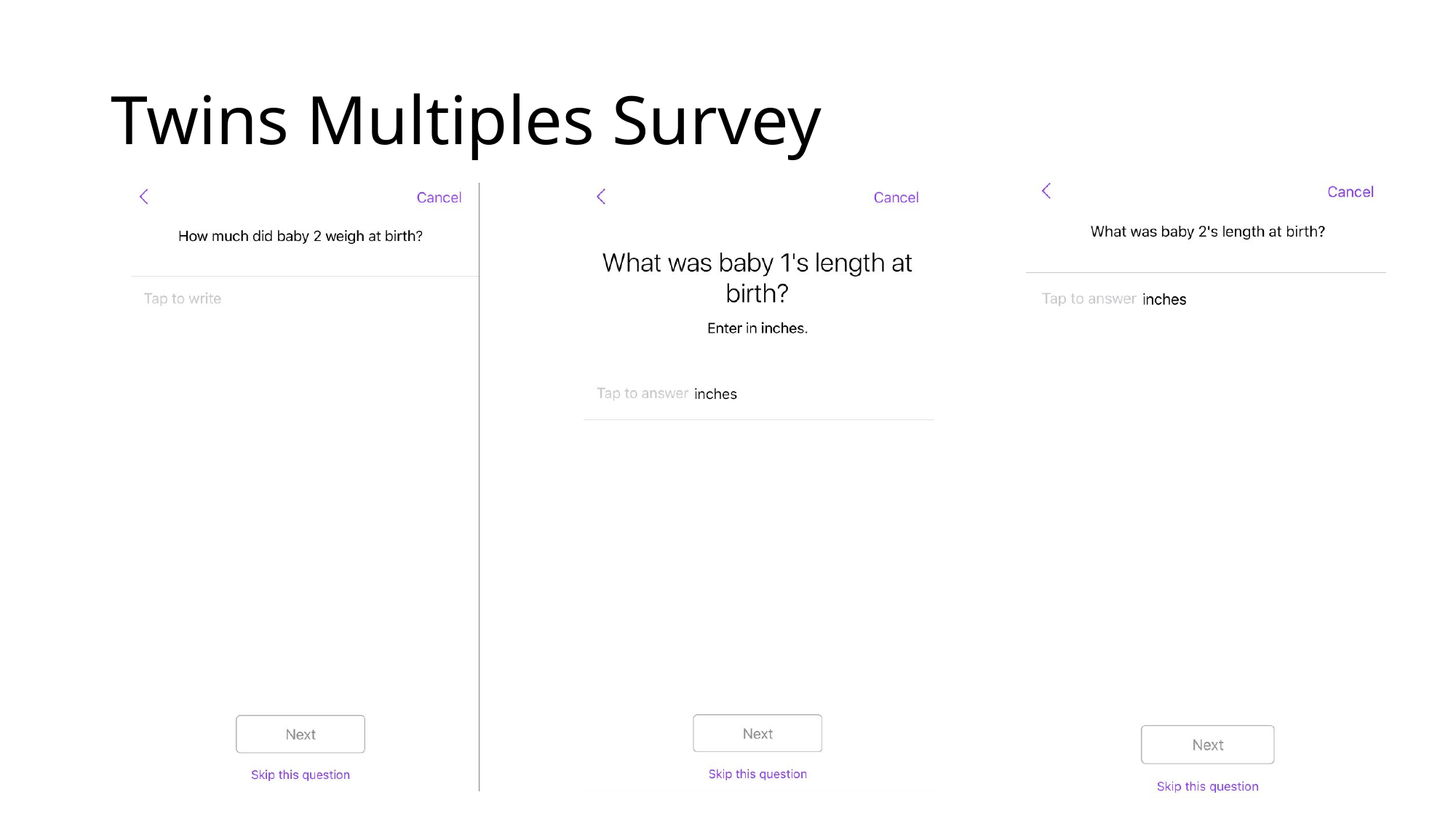

# Twins Multiples Survey

## Slide 43
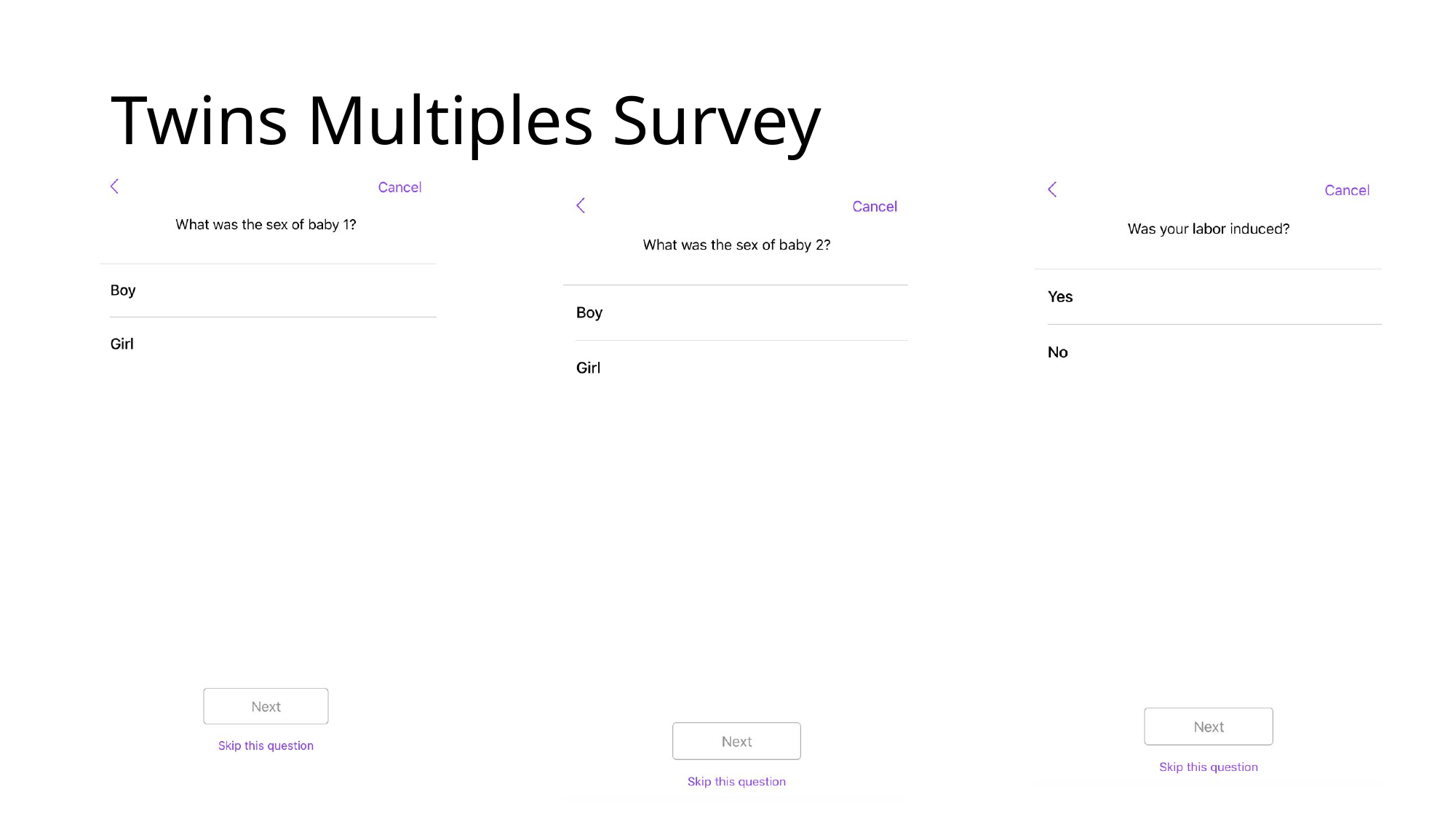

# Twins Multiples Survey

## Slide 44
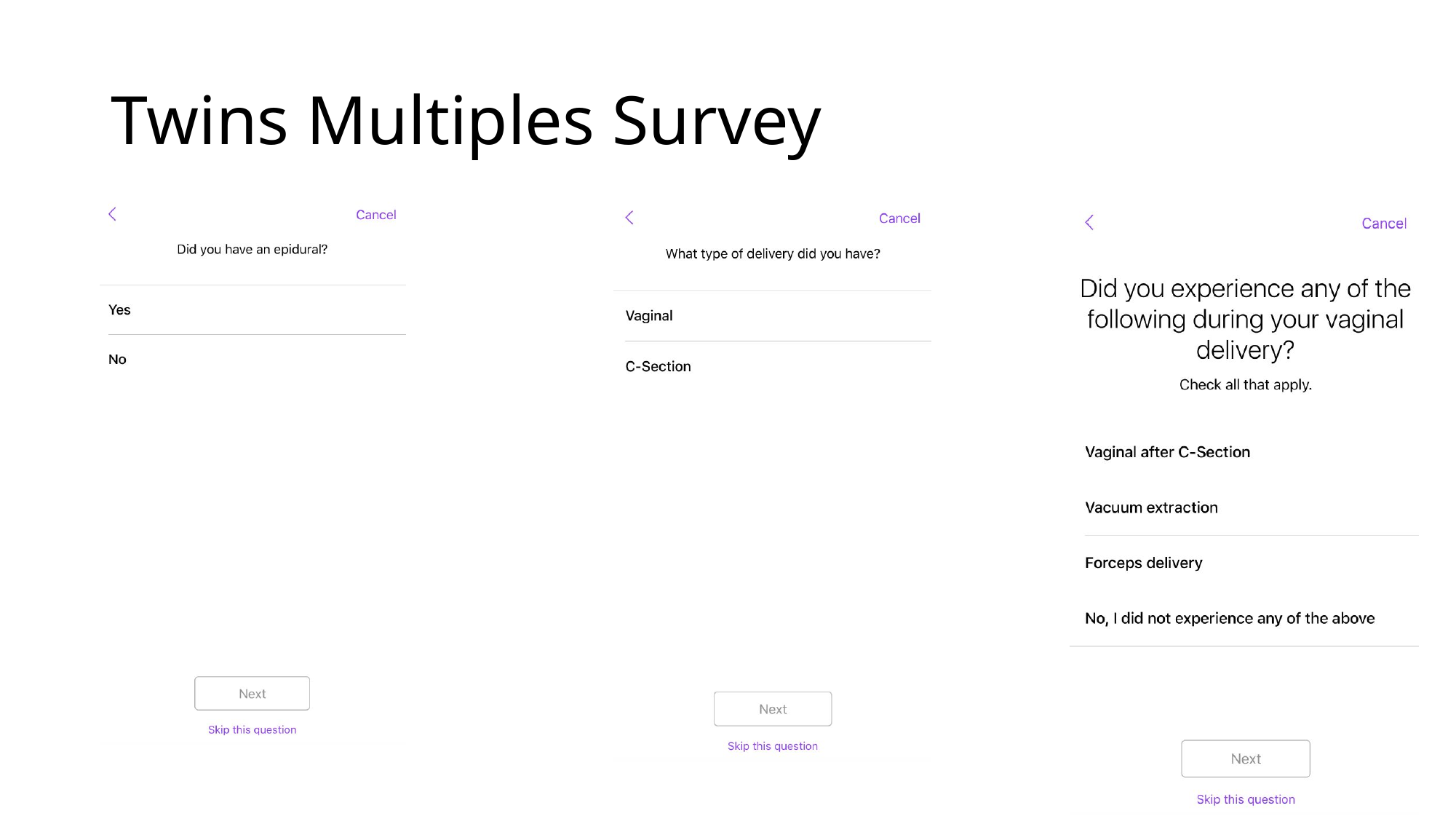

# Twins Multiples Survey

## Slide 45
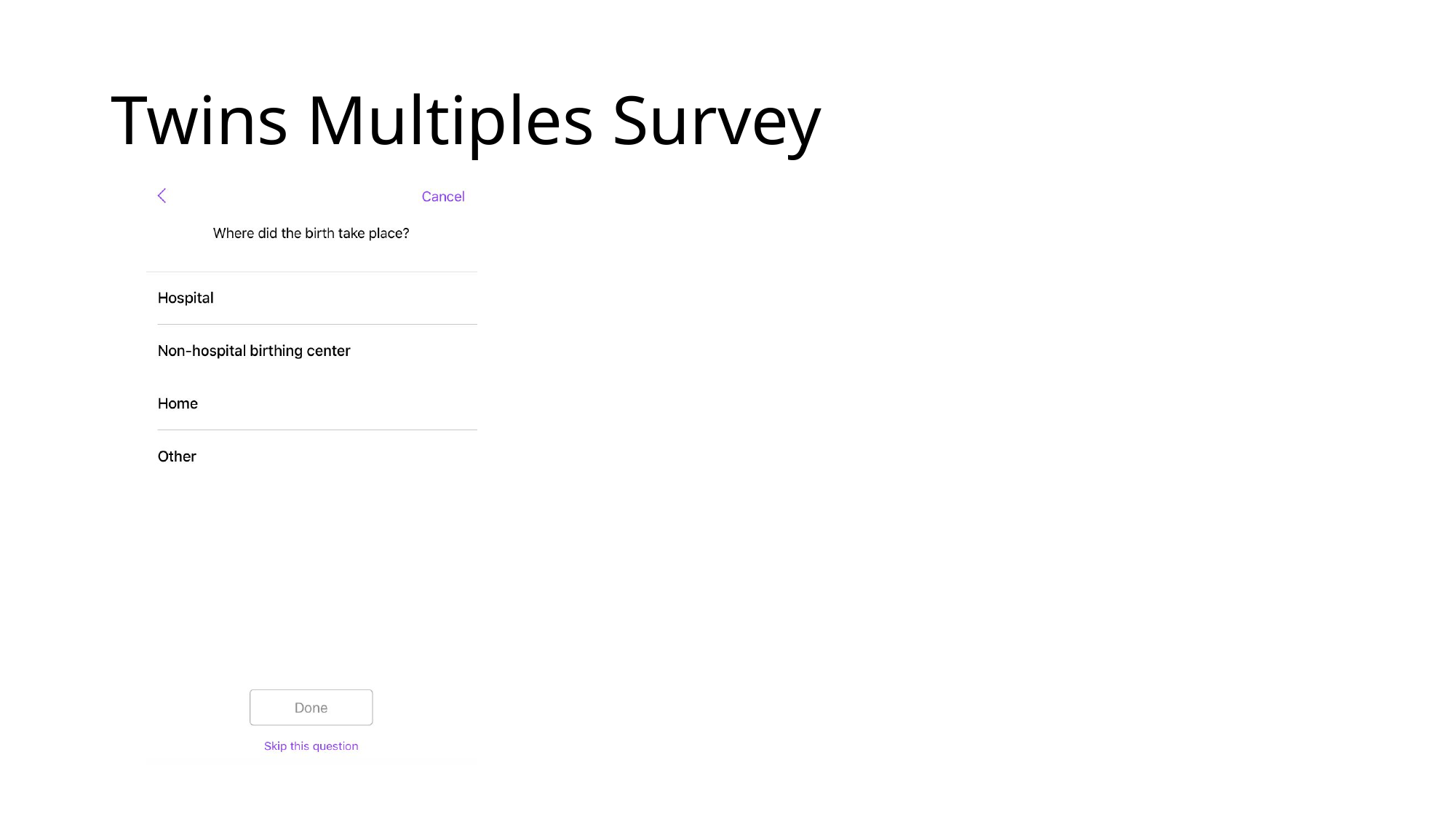

# Twins Multiples Survey

## Slide 46
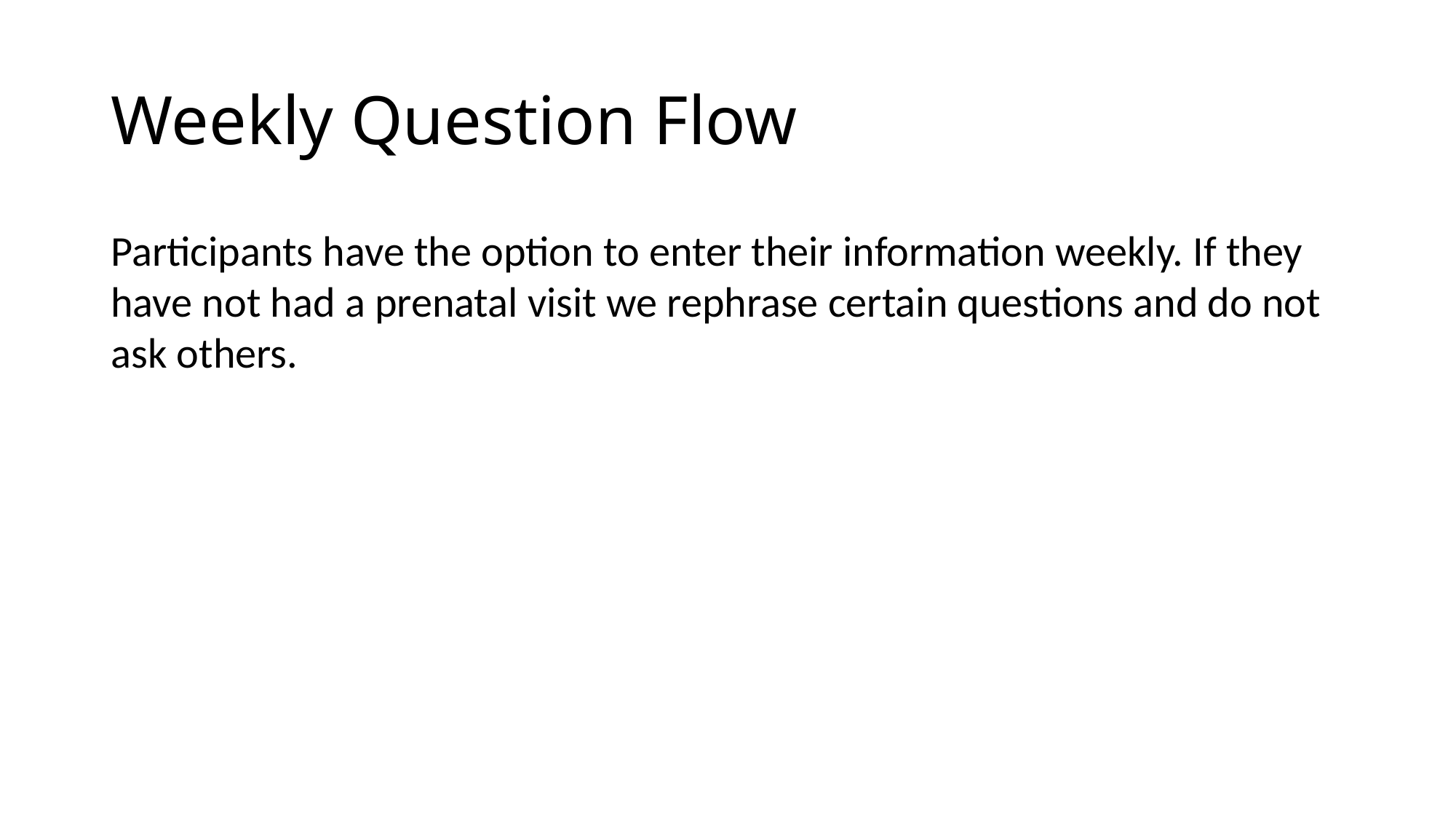

# Weekly Question Flow
Participants have the option to enter their information weekly. If they have not had a prenatal visit we rephrase certain questions and do not ask others.

## Slide 47
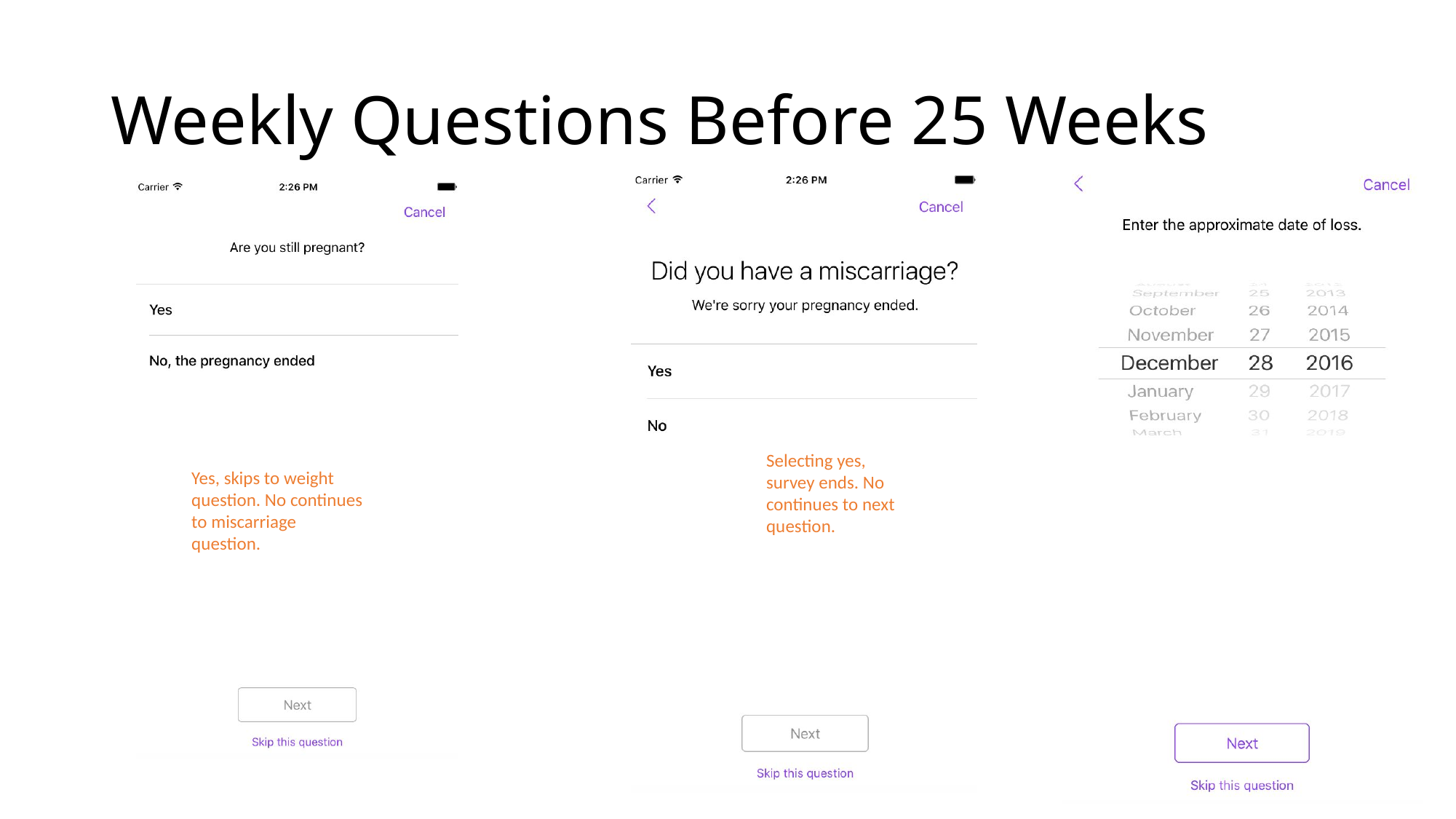

# Weekly Questions Before 25 Weeks
Selecting yes, survey ends. No continues to next question.
Yes, skips to weight question. No continues to miscarriage question.

## Slide 48
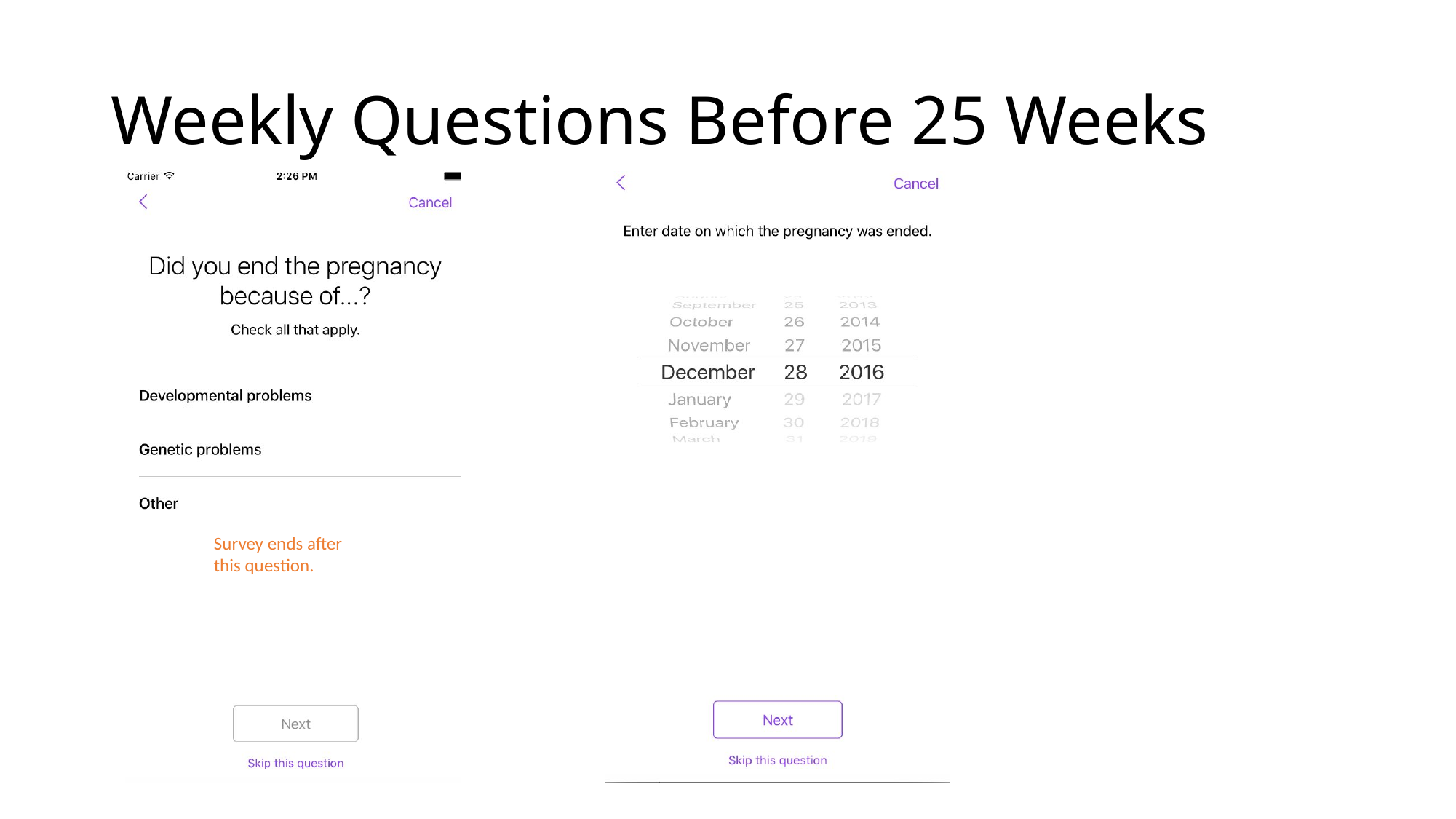

# Weekly Questions Before 25 Weeks
Survey ends after this question.

## Slide 49
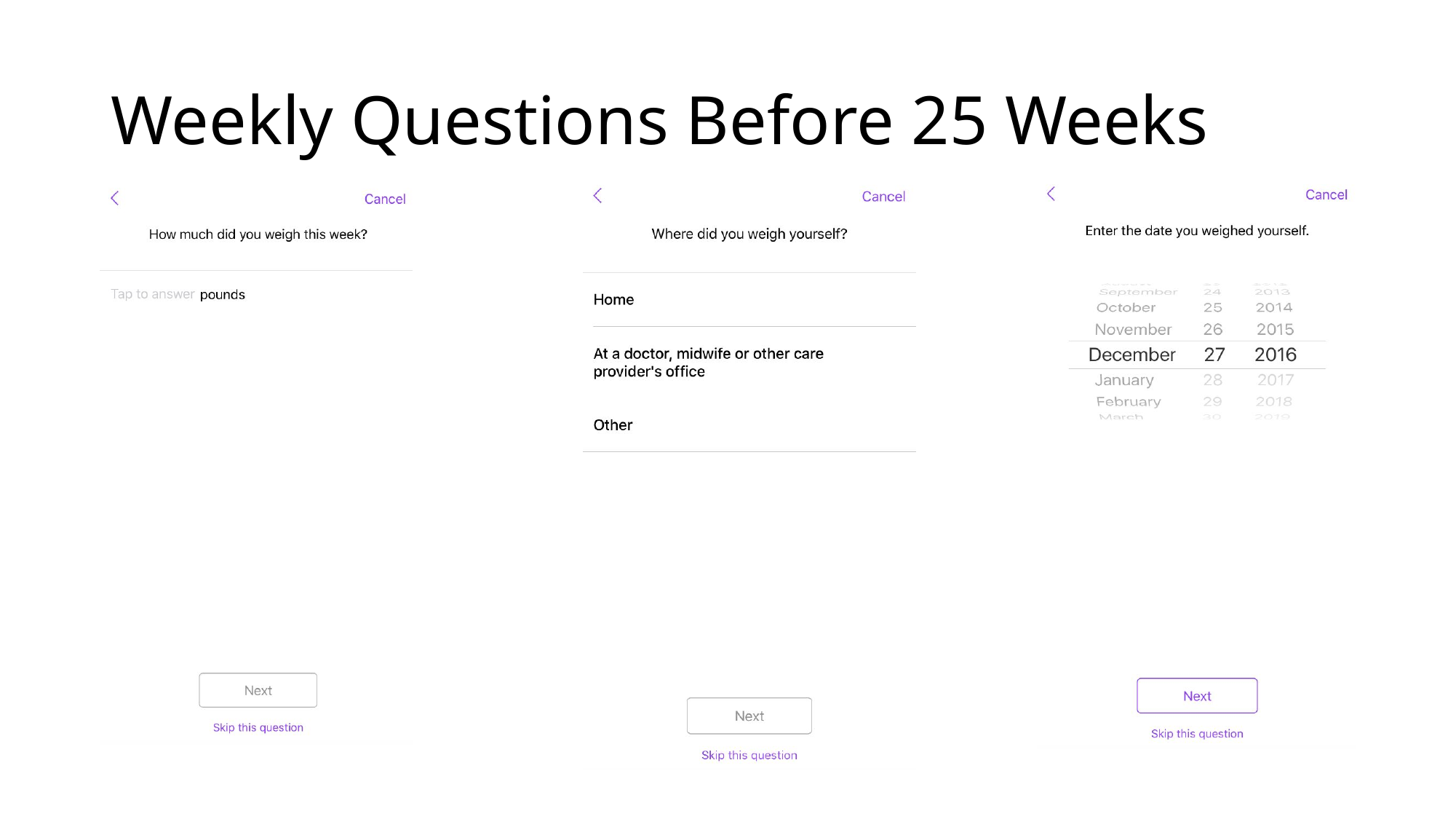

# Weekly Questions Before 25 Weeks

## Slide 50
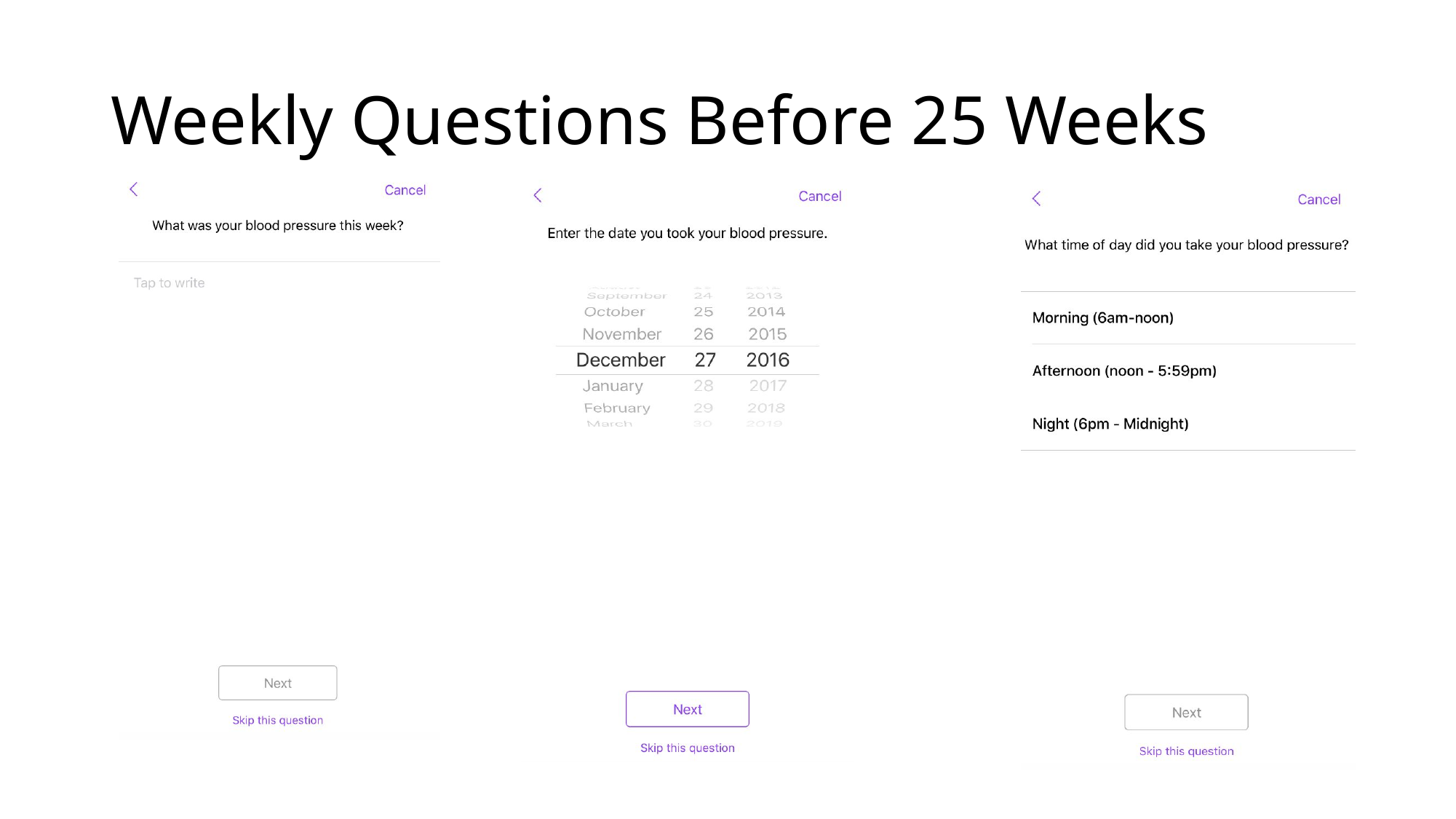

# Weekly Questions Before 25 Weeks

## Slide 51
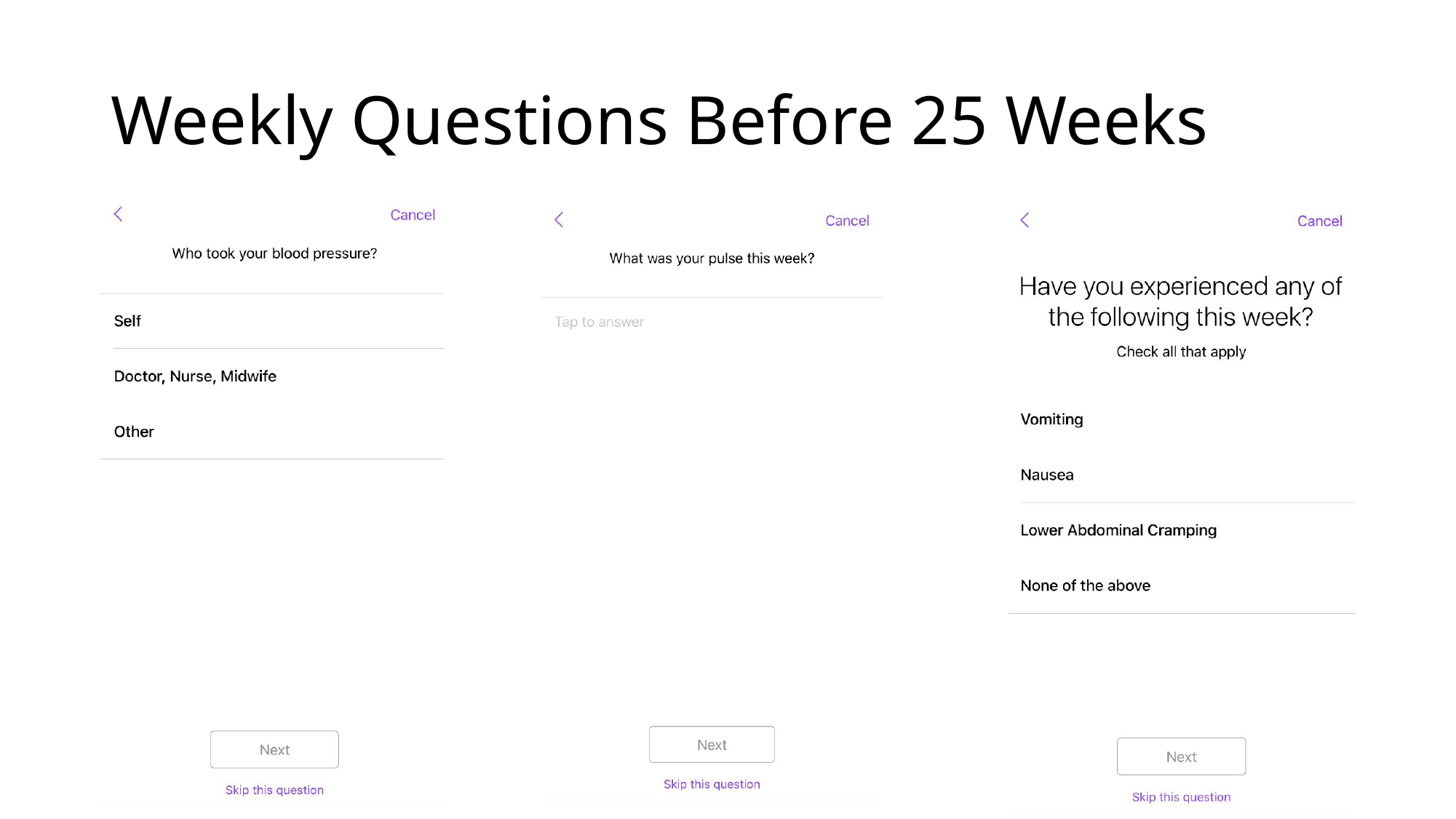

# Weekly Questions Before 25 Weeks

## Slide 52
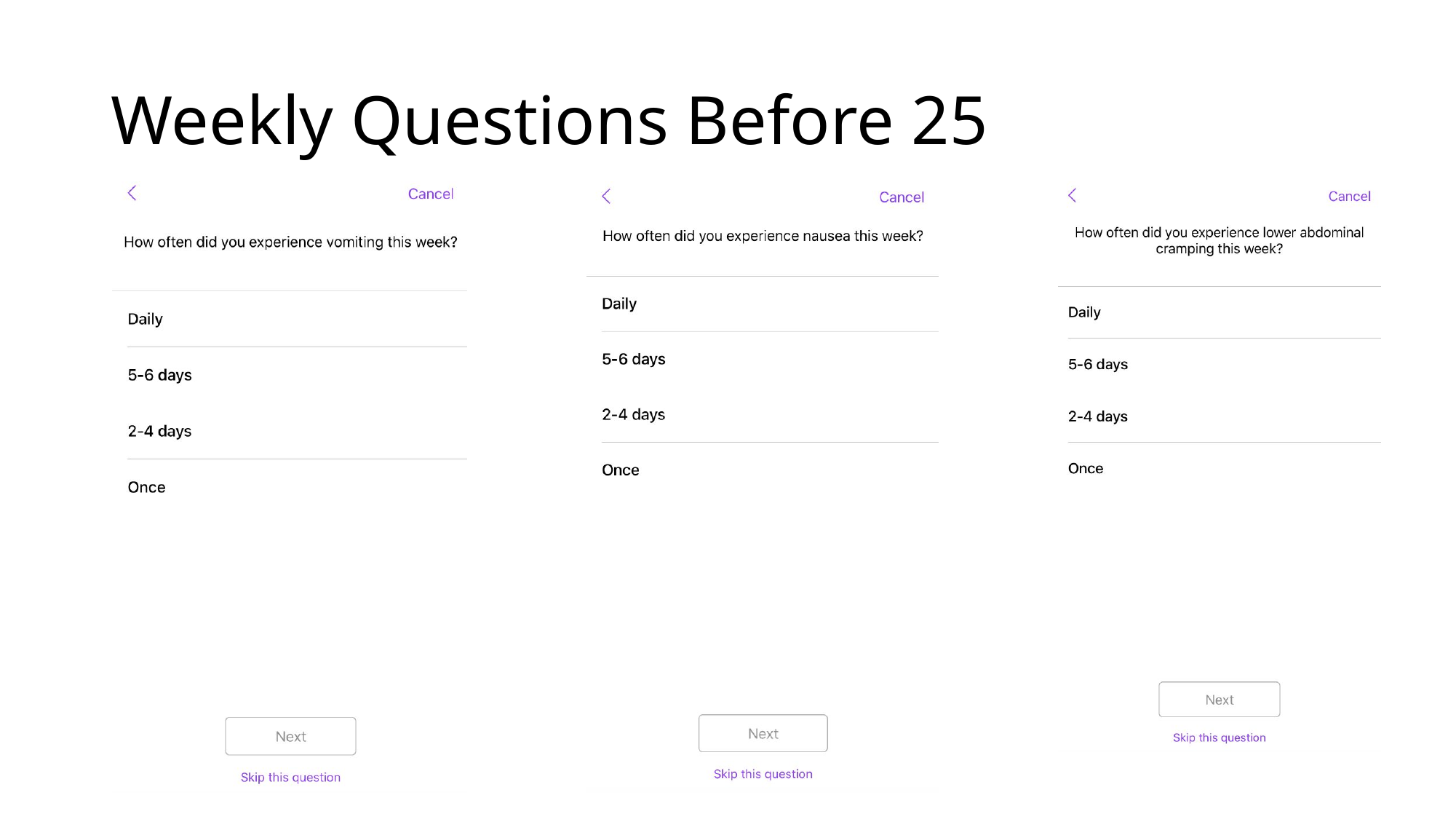

# Weekly Questions Before 25

## Slide 53
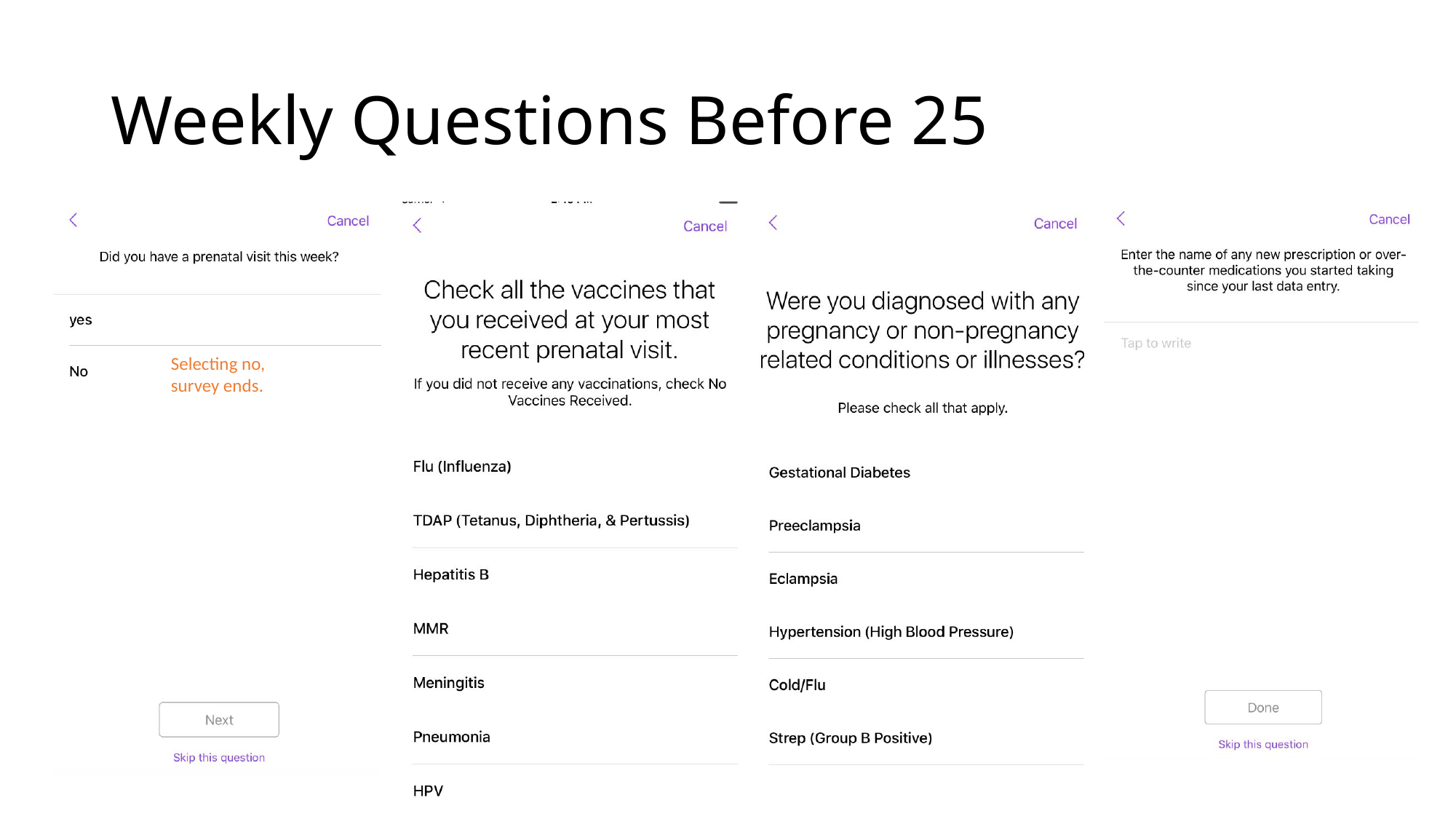

# Weekly Questions Before 25
Selecting no, survey ends.

## Slide 54
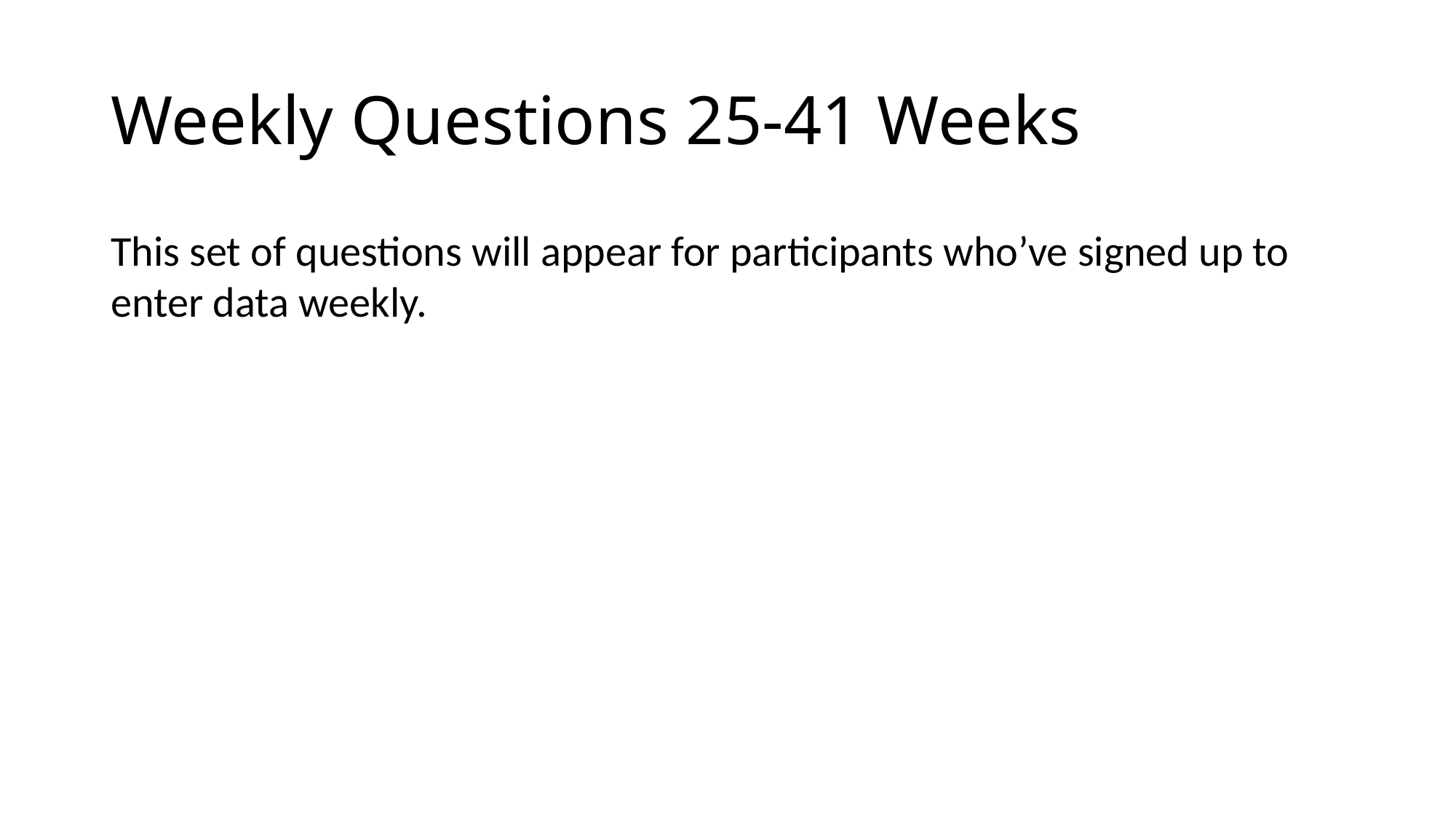

# Weekly Questions 25-41 Weeks
This set of questions will appear for participants who’ve signed up to enter data weekly.

## Slide 55
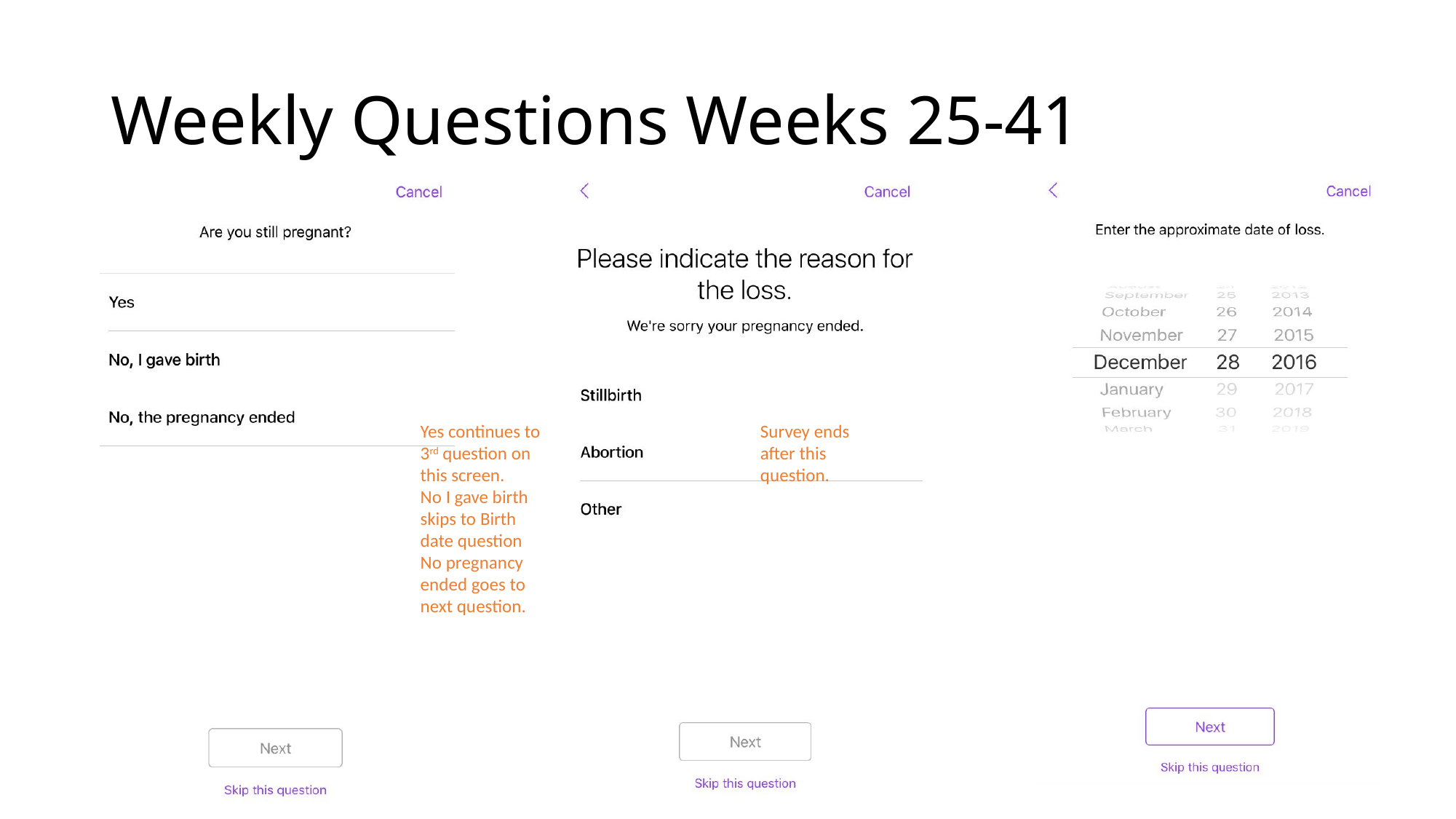

# Weekly Questions Weeks 25-41
Yes continues to 3rd question on this screen.
No I gave birth skips to Birth date question
No pregnancy ended goes to next question.
Survey ends after this question.

## Slide 56
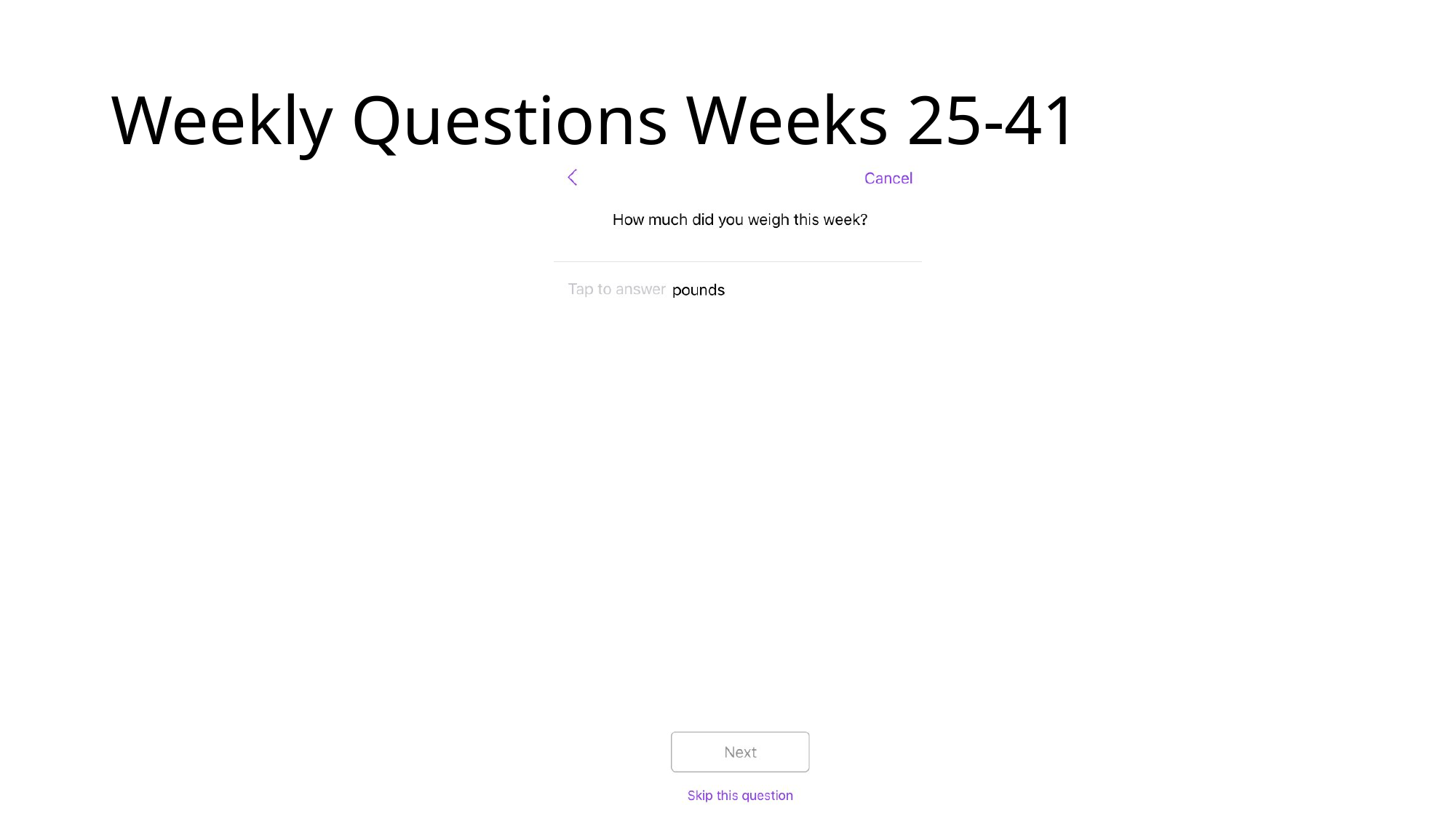

# Weekly Questions Weeks 25-41

## Slide 57
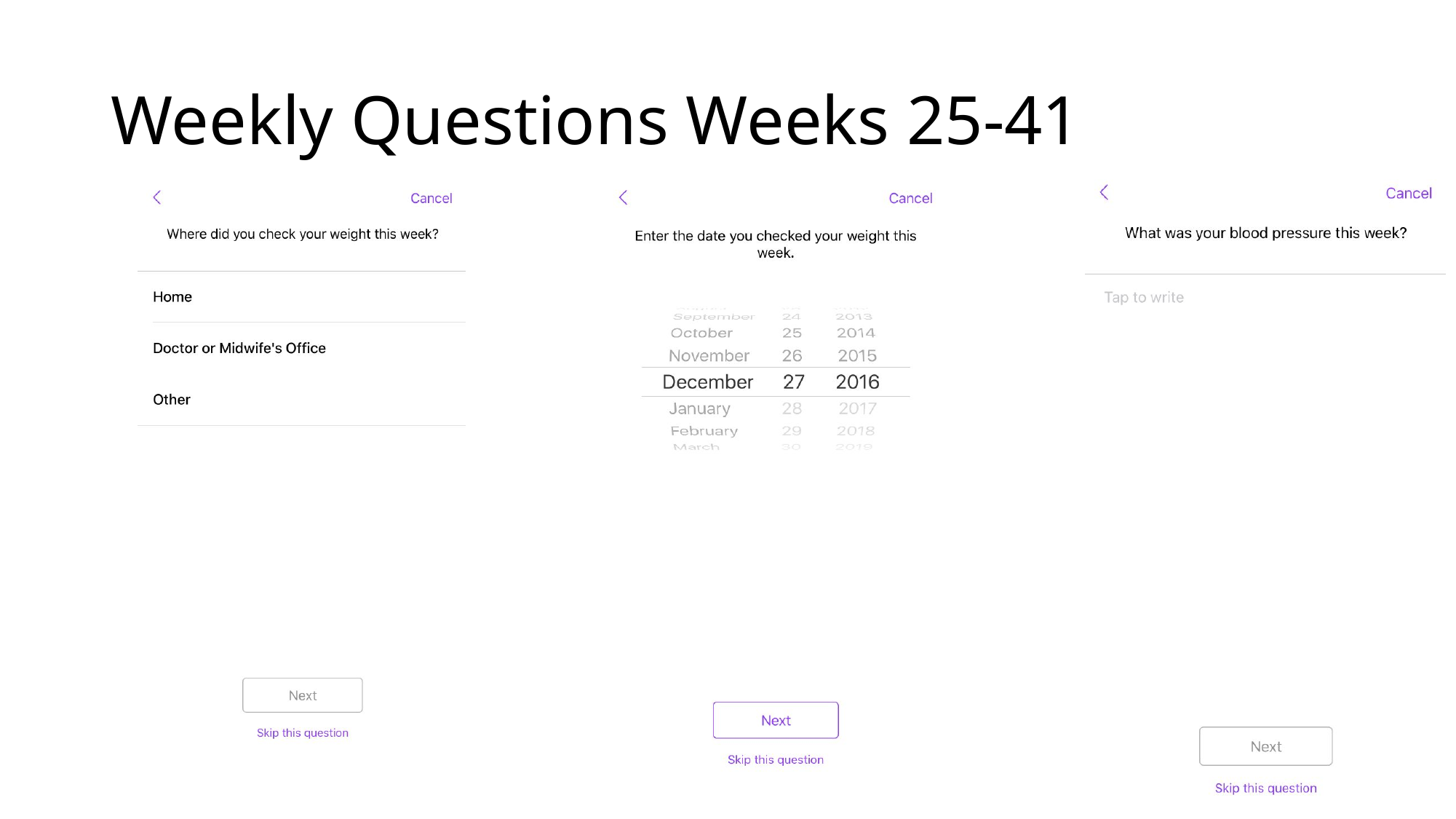

# Weekly Questions Weeks 25-41

## Slide 58
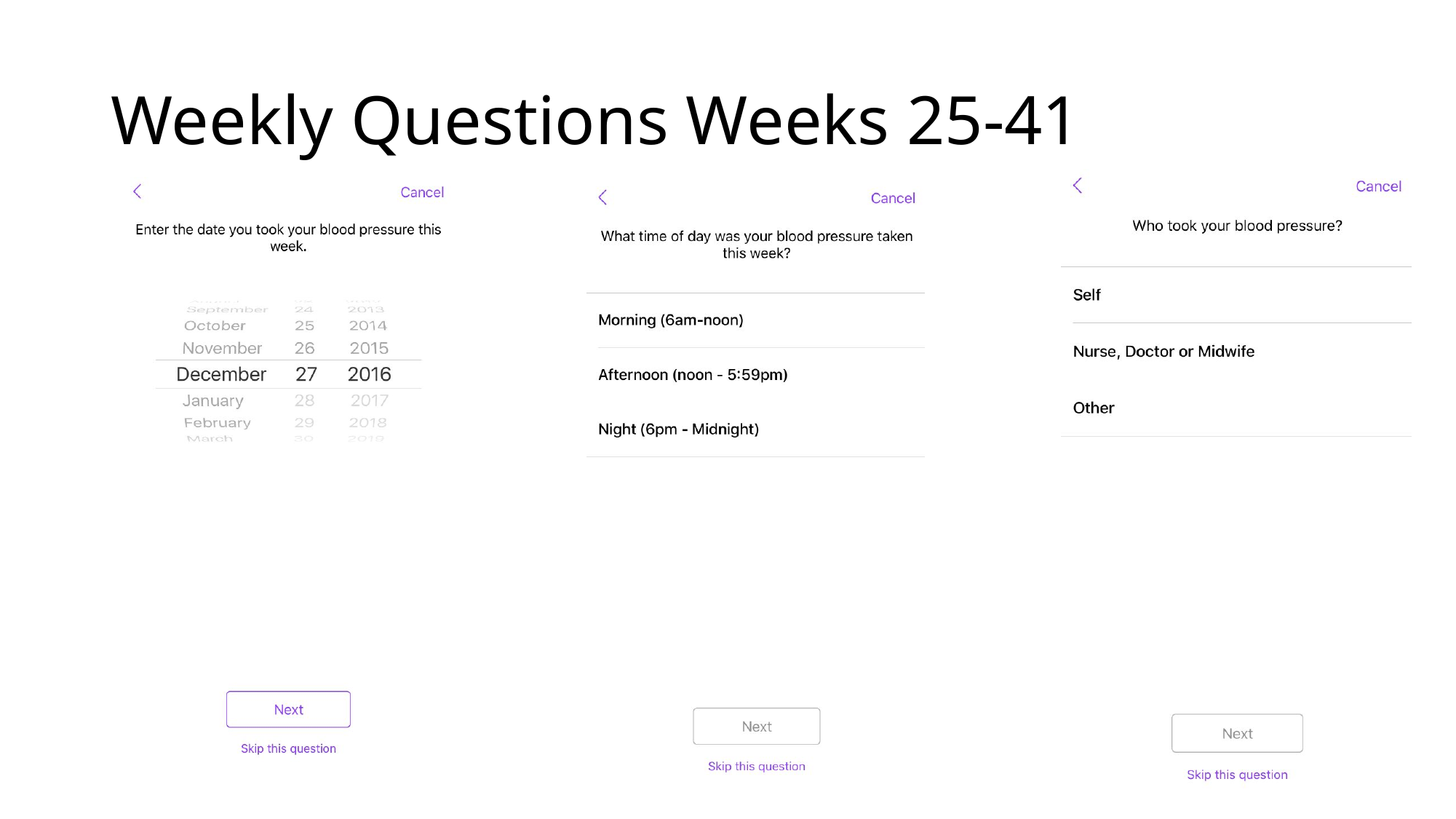

# Weekly Questions Weeks 25-41

## Slide 59
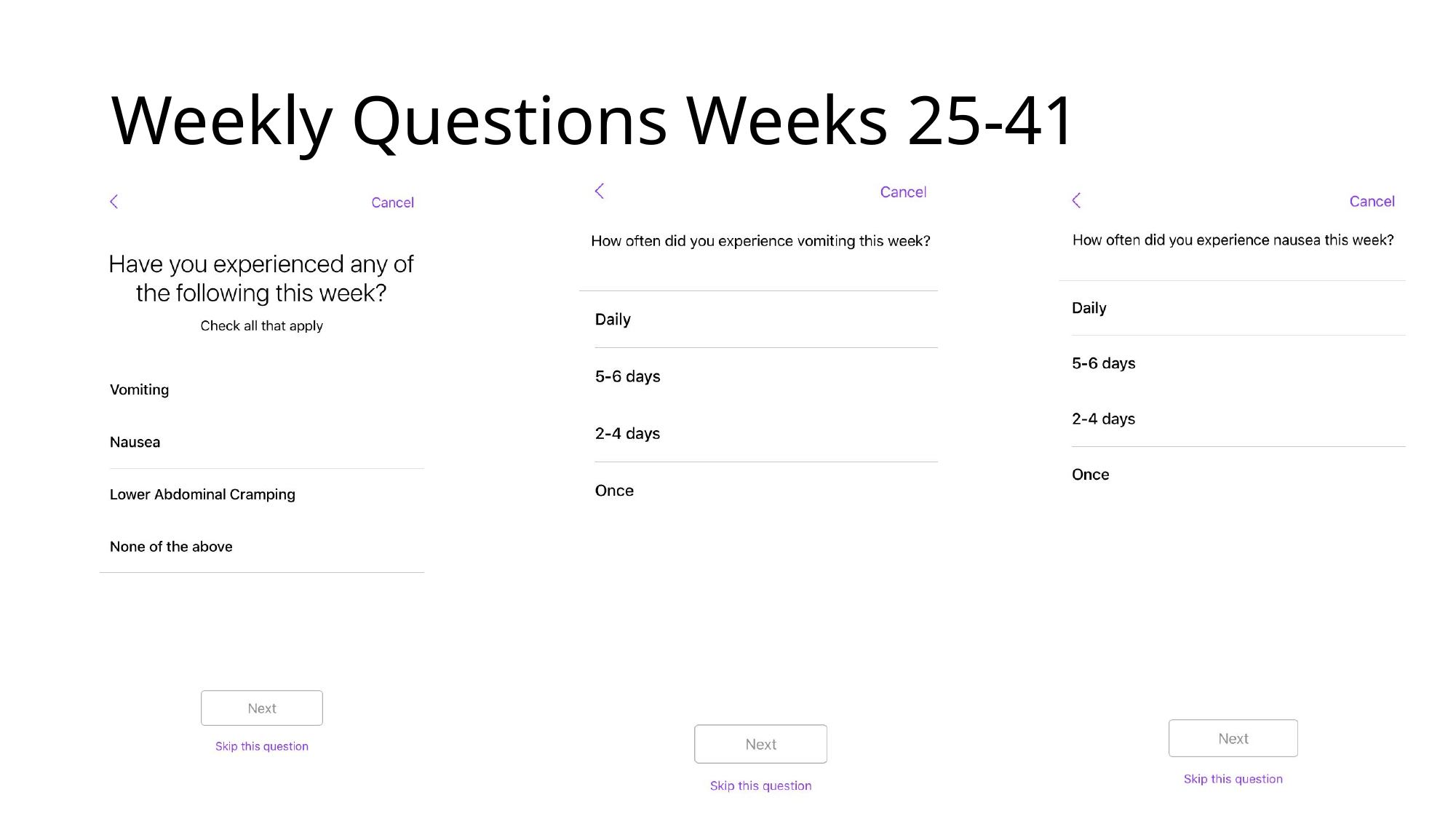

# Weekly Questions Weeks 25-41

## Slide 60
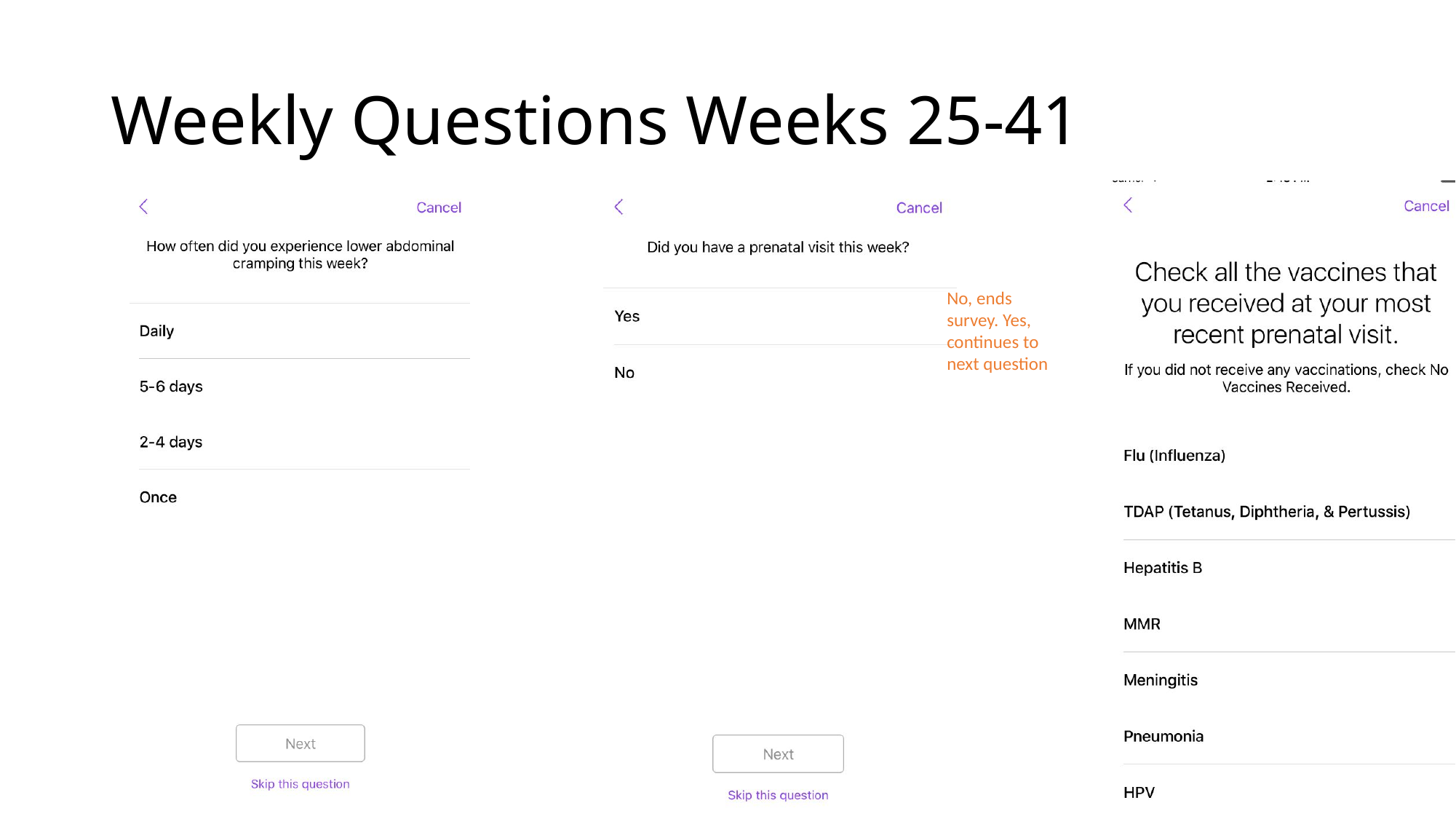

# Weekly Questions Weeks 25-41
No, ends survey. Yes, continues to next question

## Slide 61
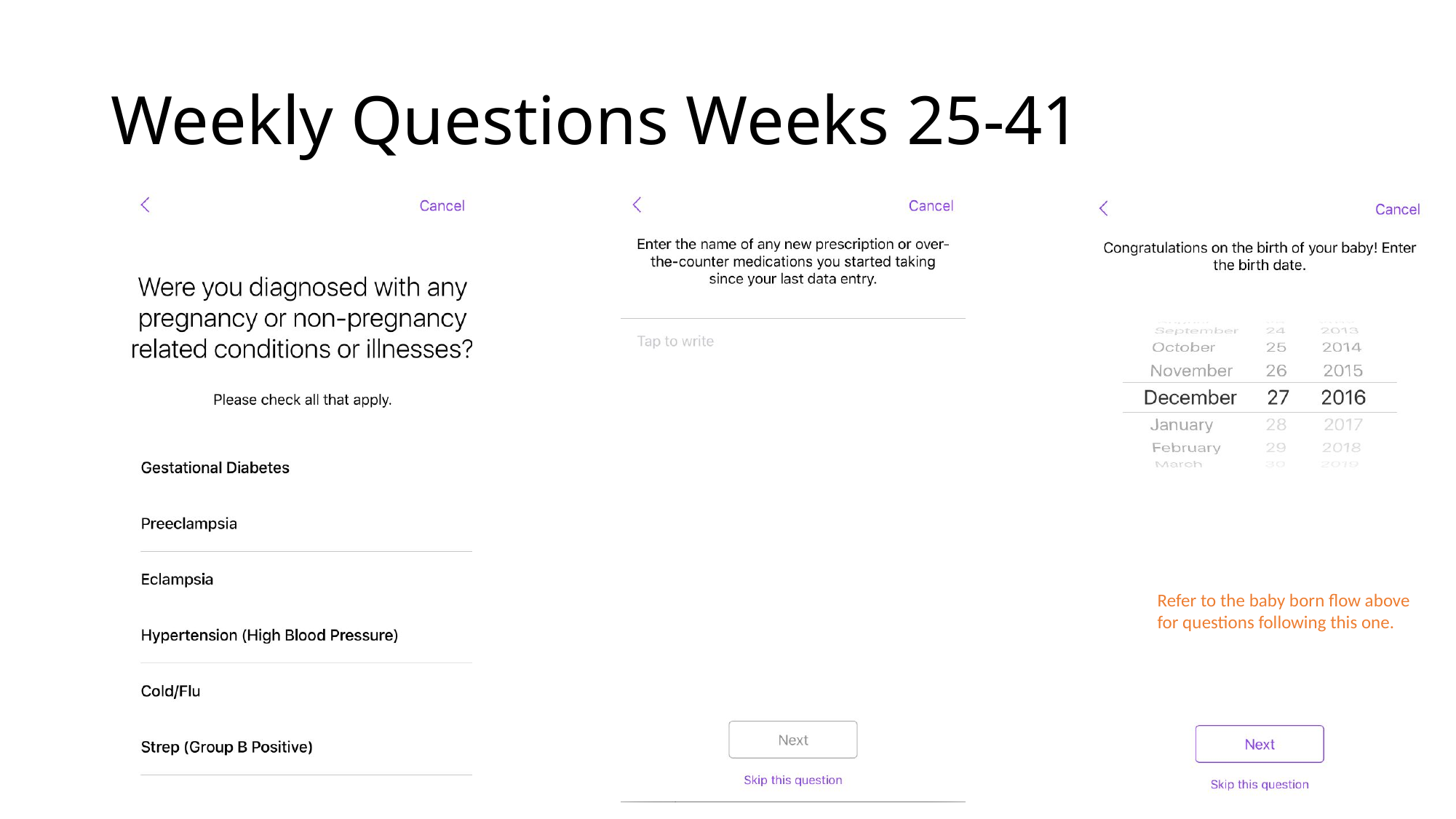

# Weekly Questions Weeks 25-41
Refer to the baby born flow above for questions following this one.

## Slide 62
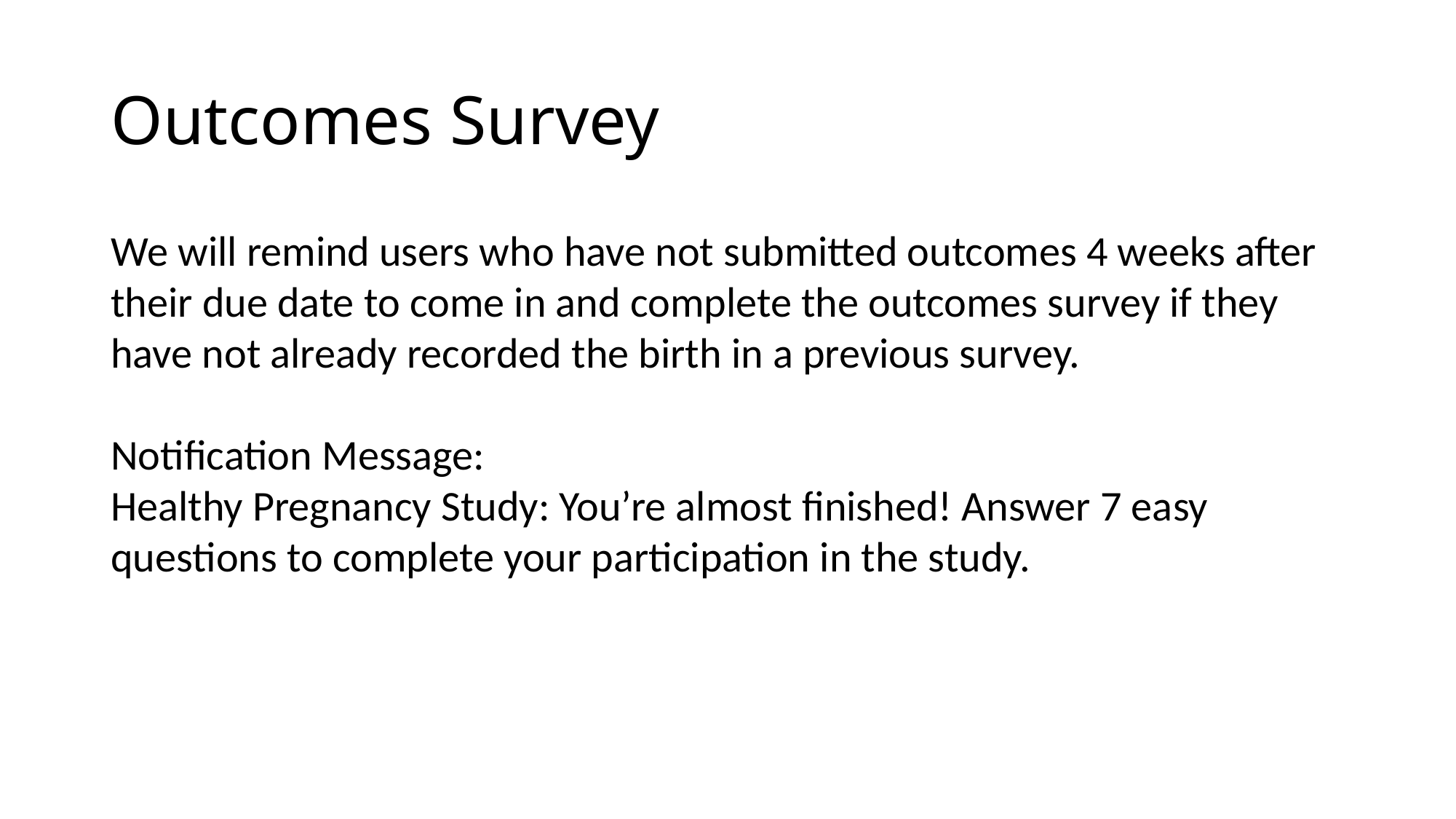

# Outcomes Survey
We will remind users who have not submitted outcomes 4 weeks after their due date to come in and complete the outcomes survey if they have not already recorded the birth in a previous survey.
Notification Message:
Healthy Pregnancy Study: You’re almost finished! Answer 7 easy questions to complete your participation in the study.

## Slide 63
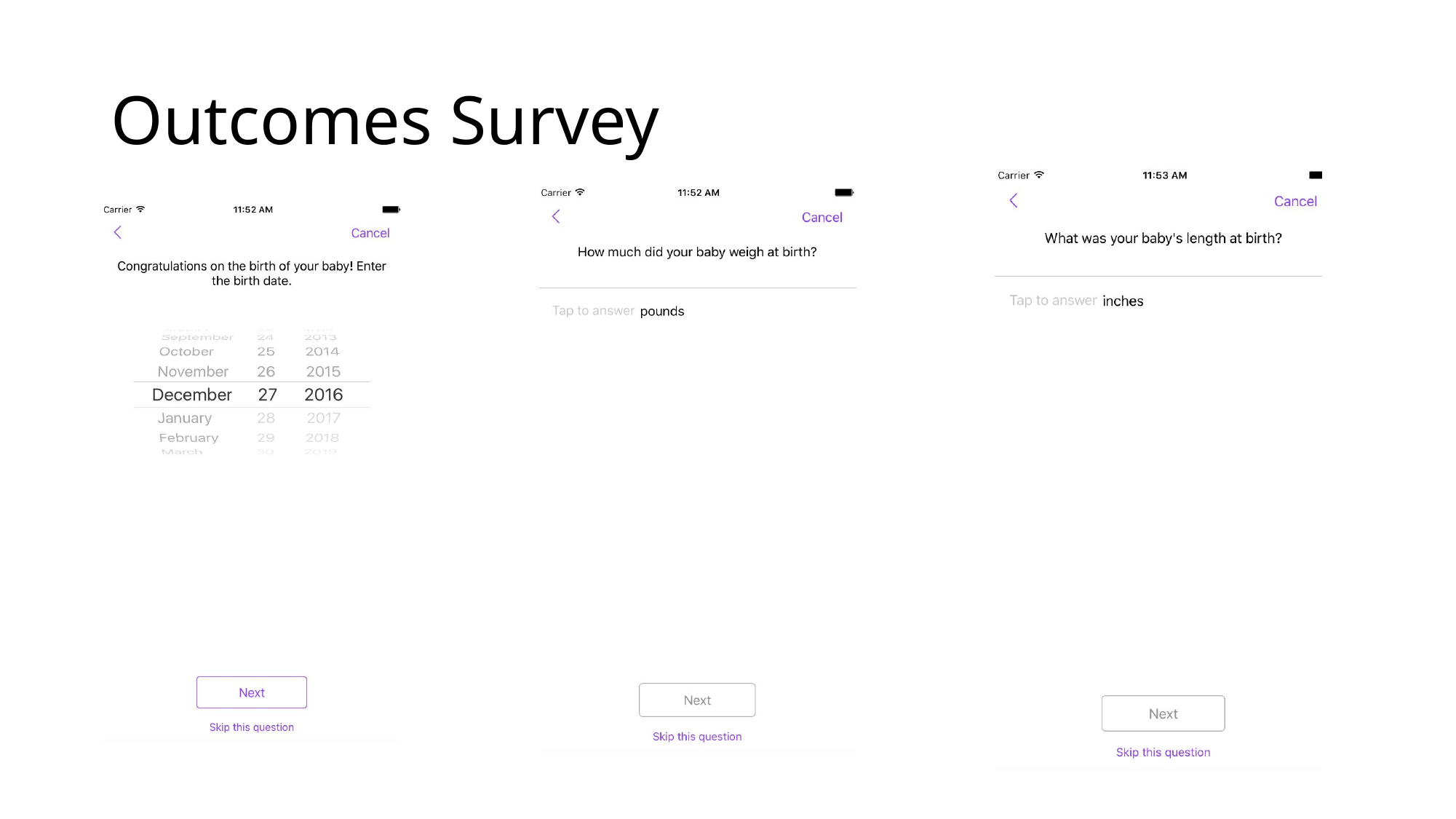

# Outcomes Survey

## Slide 64
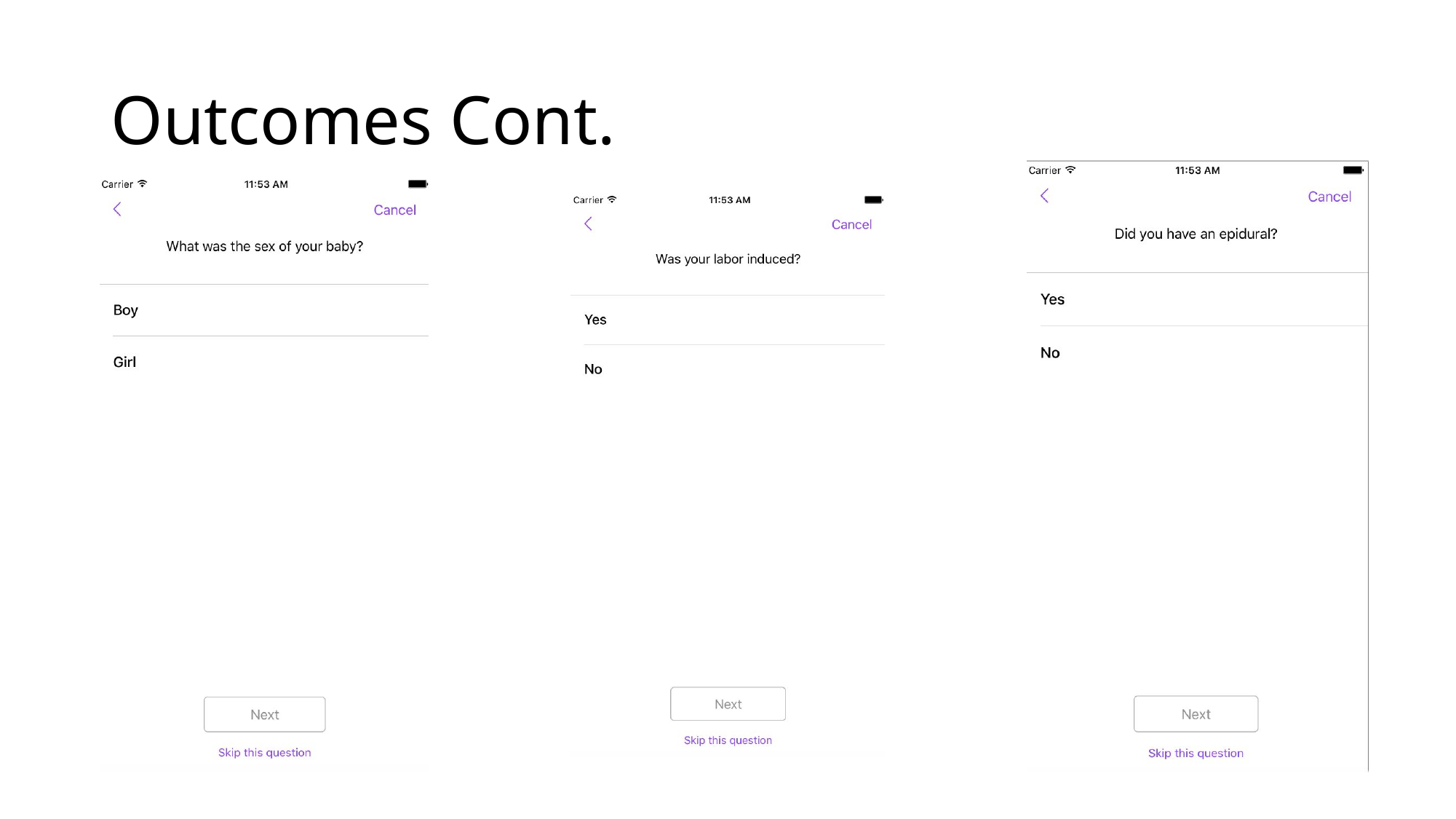

# Outcomes Cont.

## Slide 65
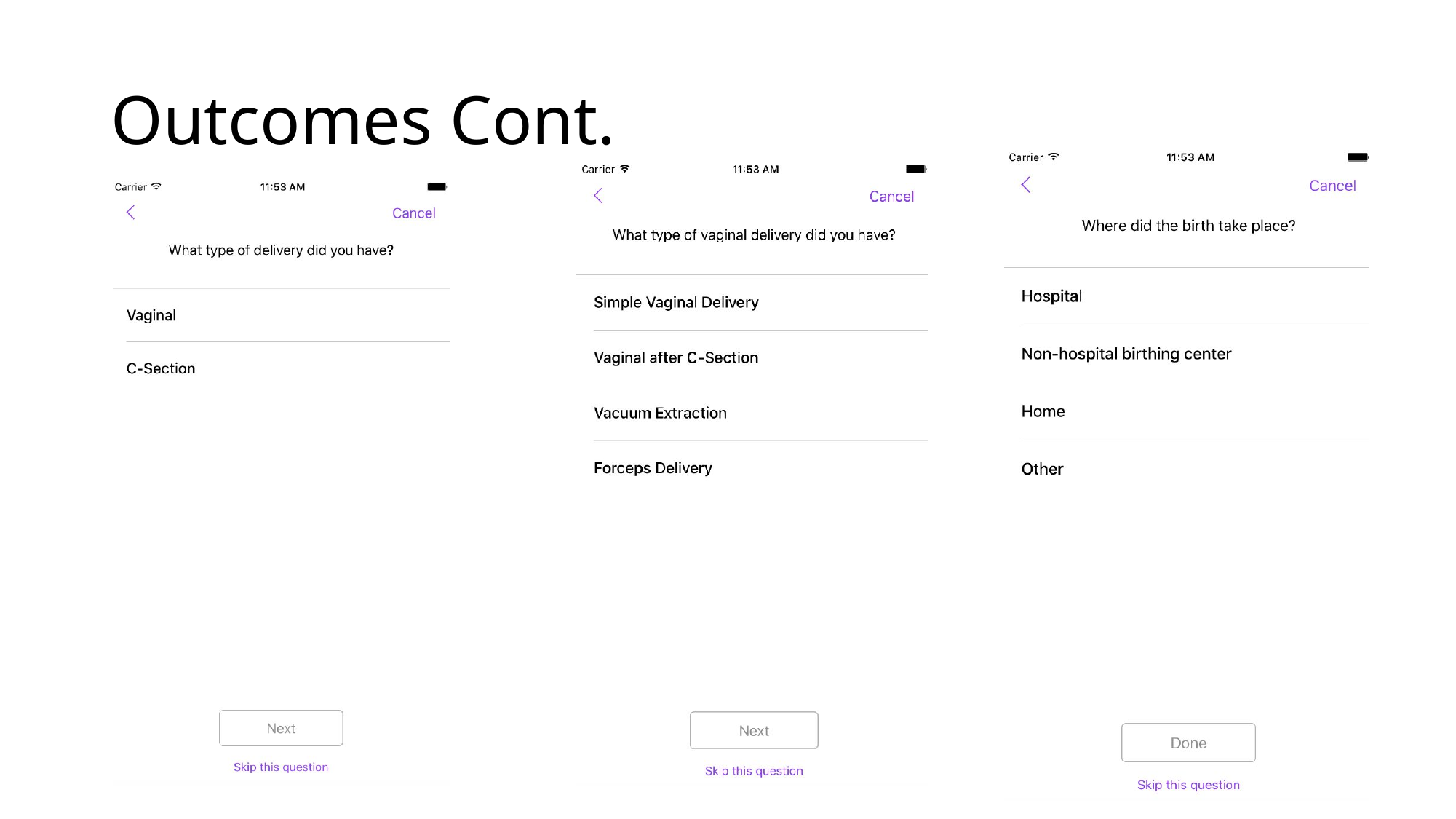

# Outcomes Cont.
